# Association between Metabolites and the Risk of Lung Cancer: A Systematic Literature Review and Meta-Analysis of Observational Studies

**DOI:** 10.3390/metabo10090362

**Published:** 2020-09-05

**Authors:** Kian Boon Lee, Lina Ang, Wai-Ping Yau, Wei Jie Seow

**Affiliations:** 1 Department of Pharmacy, Faculty of Science, National University of Singapore, Singapore 117543, Singapore; lee_kian_boon@u.nus.edu (K.B.L.); phaywp@nus.edu.sg (W.-P.Y.); 2 Saw Swee Hock School of Public Health, National University of Singapore and National University Health System, Singapore 117549, Singapore; ephanli@nus.edu.sg; 3 Department of Medicine, Yong Loo Lin School of Medicine, National University of Singapore and National University Health System, Singapore 119228, Singapore

**Keywords:** biomarkers, lung cancer, metabolomics, meta-analysis, systematic literature review

## Abstract

Globally, lung cancer is the most prevalent cancer type. However, screening and early detection is challenging. Previous studies have identified metabolites as promising lung cancer biomarkers. This systematic literature review and meta-analysis aimed to identify metabolites associated with lung cancer risk in observational studies. The literature search was performed in PubMed and EMBASE databases, up to 31 December 2019, for observational studies on the association between metabolites and lung cancer risk. Heterogeneity was assessed using the I^2^ statistic and Cochran’s Q test. Meta-analyses were performed using either a fixed-effects or random-effects model, depending on study heterogeneity. Fifty-three studies with 297 metabolites were included. Most identified metabolites (252 metabolites) were reported in individual studies. Meta-analyses were conducted on 45 metabolites. Five metabolites (cotinine, creatinine riboside, N-acetylneuraminic acid, proline and r-1,t-2,3,c-4-tetrahydroxy-1,2,3,4-tetrahydrophenanthrene) and five metabolite groups (total 3-hydroxycotinine, total cotinine, total nicotine, total 4-(methylnitrosamino)-1-(3-pyridyl)-1-butanol (sum of concentrations of the metabolite and its glucuronides), and total nicotine equivalent (sum of total 3-hydroxycotinine, total cotinine and total nicotine)) were associated with higher lung cancer risk, while three others (folate, methionine and tryptophan) were associated with lower lung cancer risk. Significant heterogeneity was detected across most studies. These significant metabolites should be further evaluated as potential biomarkers for lung cancer.

## 1. Introduction

Lung cancer is the most common form of malignancy worldwide, particularly in men, with high mortality and morbidity rates [1]. Based on histology, lung cancers are broadly classified into two major subtypes: small-cell lung cancer (SCLC) and non-small-cell lung cancer (NSCLC) [2]. The latest report by the World Health Organization identified lung cancer as the most frequent type of cancer in terms of both incidence and mortality, with 2.1 million new cases and 1.8 million deaths occurring worldwide in 2018 [1]. Lung cancer trends are strongly associated with tobacco consumption patterns [3,4,5]. Currently, lung cancer incidence and mortality rates are declining in the more developed countries [3,4], which may partially be attributed to the lower prevalence of tobacco smoking [6]. However, lung cancer trends are increasing in Asia and Africa, which is associated with increasing prevalence of tobacco smoking [3,7].

Although lung cancer is a deadly disease, it can be treated if detected early. The five-year survival rates for localized lung cancer was 56%, as compared with 5% for metastasized lung cancer [8]. However, despite efforts in lung cancer research, early detection and diagnosis remain as major challenges [9]. A considerable proportion of patients present with late-stage lung cancer at diagnosis (46.8–61.2% for NSCLC and 61.3–82% for SCLC) [10]. Considering the high mortality rate of late-stage lung cancer and the difficulty of early diagnosis, identifying potential biomarkers for the detection of early-stage lung cancer is paramount.

In recent years, the use of metabolomics, which is the study of metabolites in biological specimens [11], in lung cancer research has been a subject of great interest. Compared to genomics, transcriptomics and proteomics, which focus on the upstream processes of metabolism, metabolomics directly measures the metabolic profile of an organism, thereby providing a more precise method to detect changes in metabolism [12]. Since malignant cells, including lung cancer cells, have substantially altered genome and metabolism [13,14,15,16], the identification of metabolic changes is a potentially viable strategy for elucidating lung cancer etiology and identifying potential biomarkers.

Numerous studies have explored the association between metabolite levels and lung cancer risk [17,18,19,20,21]. However, most studies have been small-scale, with varying direction and strengths of association across studies. Furthermore, the type of biological samples used have been inconsistent across studies. While earlier reviews on this topic have identified three major classes of metabolites (namely amino acids, fatty acids/lipids and metabolites involved in cellular energy production) to be associated with lung cancer [22,23], to date, the association between levels of each metabolite and lung cancer risk has not been quantitatively evaluated. It is worthwhile to examine both alterations in levels of cellular metabolites as well as smoking-related metabolites. Changes in cellular metabolite levels might be related to intrinsic body alterations, while changes in smoking-related metabolites might be a consequence of patient smoking and other environmental exposures. Therefore, a need to systematically review and quantitatively synthesize the results from all pertinent studies is warranted, in order to identify potential biomarkers for validation studies in the future.

In this systematic literature review and meta-analysis, we aimed to provide a review of the current understanding of the association between metabolites and lung cancer risk based on available evidence from published observational studies.

## 2. Methods

### 2.1. Literature Search Strategy

This systematic literature review was performed based on the Preferred Reporting Items for Systematic Reviews and Meta-analyses (PRISMA) guidelines [24] (List S2). PubMed and EMBASE databases were searched from inception to 31 December 2019. The search terms used included “metabolites”, “metabolomics”, “lung cancer” and its variants (Table S1). Titles and abstracts of searched articles were screened by two reviewers before retrieving full texts of potentially relevant articles for further evaluation. One reviewer then independently evaluated the full text before making the decision to include the study. The reference lists of included articles were further hand-searched to identify additional relevant articles.

### 2.2. Study Selection Criteria

Studies were included if the following inclusion criteria were met: (1) were observational studies; (2) reported at least one metabolite; and, reported either the (3a) estimated hazard ratio (HR)/odds ratio (OR)/risk ratio (RR) for the association between levels of metabolite and any type of lung cancer; or, (3b) actual metabolite concentrations for lung cancer patients and controls. If more than one report were published using data from the same study, we included either the report with the most detailed information, or the most recent report, in the case where the reports provided a similar level of details.

Studies were excluded if they met any of the following exclusion criteria: (1) were non-clinical studies or randomized controlled trials, case reports, case series, reviews or conference abstracts; (2) reported on metastasized lung cancer or recurrent cancer only; (3) included cancer patients who were already on treatment prior to recruitment; (4) included controls with another disease state for non-self-controlled studies; (5) did not provide sufficient data for analysis; or, (6) were not published in English or Chinese.

### 2.3. Data Extraction

For studies that met the eligibility criteria, one reviewer performed data extraction using a standardized form. Data extracted from each study were study design, characteristics of study participants, type of metabolites, type and stage of lung cancer (if reported), type of biological samples used and the number of participants for each metabolite and outcome. For studies that reported the association between levels of metabolite and lung cancer (hereafter termed as categorical studies), the HR/OR/RR and 95% confidence interval (CI) between the highest and lowest category from the models that adjusted for the most covariates were extracted. For studies that reported the concentration of metabolites in lung cancer patients and controls (hereafter termed as concentration studies), the mean and standard deviation (SD) of metabolite concentration were extracted.

For studies that reported the relevant data in only graphical form [25,26], the data were estimated based on the graphical data presented.

One study [27] presented data that were partially reported in a previous study [28]. For this study [27], only the data that were not previously reported were extracted for further analysis.

One study included recurrent cancer cases in their pool of participants [29]. Since the study reported sufficient data to exclude recurrent cases, the mean and SD were recalculated with the recurrent cases excluded.

For one study that reported standard error (SE) instead of SD [30], SD was calculated as:

(1) $$SD\;=\;SE\;\times \;\sqrt{n},$$

where n is the number of study participants.

One study reported the concentration of metabolites in μg/L [31]. The data were transformed into μmol/L using the following formulae:

(2) $$Mean=\frac{Mean_{\mu g}}{M_{r}},$$

(3) $$SD=\frac{SD_{\mu g}}{M_{r}},$$

where $Mean_{\mu g}$,$\;SD_{\mu g}$ is the mean and SD (reported in μg/L), and $M_{r}$ is the molecular weight of the metabolite. The molecular weights of the metabolites were retrieved from the Kyoto Encyclopedia of Genes and Genomes (KEGG) database [32].

For studies that reported data for every participant individually [33,34], the mean and SD were calculated as:

(4) $$Mean=\frac{\sum^{\;}c_{i}}{n},$$

(5) $$SD=\sqrt{\frac{\sum^{\;}{\left(c_{i}-Mean\right)}^{2}}{n-1}},$$

where $c_{i}$ is the concentration of metabolite for each participant and n is the number of study participants.

For studies that reported their data as either median, first and third quartiles [25,35] or median and range [36] in lieu of mean and SD, we estimated the mean and SD using the following formulae [37]:

(6) $$Mean=\frac{q_{1}+q_{2}+q_{3}}{3},$$

(7) $$SD=\frac{q_{3}-q_{1}}{2\times Z_{inv}\left(\frac{0.75n-0.125}{n+0.25}\right)},$$

(8) $$Mean=\frac{m+2q_{2}+M}{4},$$

(9) $$SD=\frac{M-m}{2\times Z_{inv}\left(\frac{n-0.375}{n+0.25}\right)},$$

where $q_{1},\;q_{2},q_{3}$ are the first, second and third quartiles, m,M are the minimum and maximum, n is the number of study participants and $Z_{inv}$ is the inverse function of the standard normal distribution.

### 2.4. Assessment of Methodological Quality

The nine-point Newcastle–Ottawa Scale (NOS) was used to evaluate the methodological quality of observational studies in terms of three broad categories: selection of study groups; comparability of study groups; and the determination of either the exposure (for case-control studies) or outcome (for cohort studies) of interest [38]. Scoring was performed independently by two reviewers (K.B.L and L.A.) and discrepancies in quality scores were resolved by discussion and re-examination of the full-text articles. Any outstanding discrepancies were resolved by discussion with another investigator (W.J.S.).

For self-controlled case-control studies (4 studies) [33,39,40,41], we used a modified version of the NOS by excluding the question on the definition of controls, as the criterion was not relevant to the studies. The overall quality scores for these studies were then scaled to a maximum score of nine points, in order to facilitate comparison across all studies.

For articles that reported data from more than one cohort (2 studies) [42,43], we evaluated the quality of each cohort separately and assigned an overall score by taking the mean of the scores from the cohorts.

The criterion for defining a high-quality study was neither defined by the original authors of NOS, nor was it validated in the current literature. Nevertheless, we classified studies as low, moderate or high quality based on the scores of 0–3 points, 4–6 points or 7–9 points, respectively [44].

### 2.5. Statistical Analysis

Data were grouped based on the metabolite and the type of biological sample used in the quantification of the metabolite. Meta-analyses were conducted if the metabolite from a biological sample type was reported in at least two studies. For categorical studies, the reported strength of association and the 95% CI were natural log-transformed before statistical analyses were performed [45]. The results were presented as estimated effect size and 95% CI. For concentration studies, the results were presented as weighted mean difference (WMD) and 95% CI. Stratified analyses were performed based on smoking status and gender.

Statistical heterogeneity across studies and subgroups was assessed using the I^2^ statistic and the p-value from the Cochran’s Q test. The random-effects model [46] was used to account for significant heterogeneity across studies if I^2^ was > 40% and p-value was < 0.1, while the fixed-effects model was used to obtain more precise estimates when either condition was not met [45]. The inverse variance approach was used for weighting of the individual studies. All statistical analyses were performed using Stata [47], with a *p*-value < 0.05 considered as statistically significant.

## 3. Results

### 3.1. Eligible Studies

**Figure 1:**
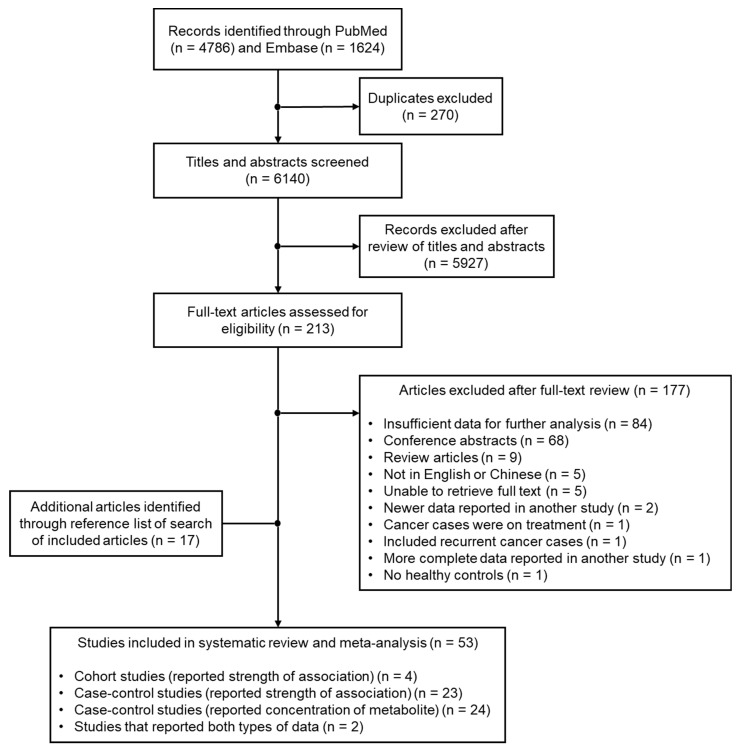
Flowchart of the literature search process and selection of studies.

The literature search process is shown in Figure 1. Of the 6140 unique articles that were identified based on the literature search strategy, 213 articles were included for full-text review. A total of 53 studies were included in the systematic literature review, of which, 27 studies were categorical studies [42,43,48,49,50,51,52,53,54,55,56,57,58,59,60,61,62,63,64,65,66,67,68,69,70,71,72], 24 studies were concentration studies [25,26,27,28,29,30,31,33,34,35,36,39,40,41,73,74,75,76,77,78,79,80,81,82], and two studies reported both the metabolite concentration and its association with lung cancer risk [83,84]. Most studies (84 studies) with full-texts retrieved for review were subsequently excluded because they did not report sufficient data for further analysis, such as reporting the fold-change in metabolite concentrations between lung cancer patients and controls, instead of actual concentrations of metabolites.

Two studies [85,86] were excluded from the review as more recent studies on the same group of participants were published [28,58,67]. One study [87] was excluded from the review as the more complete set of data for the same group of participants was published in another study [59].

### 3.2. Study Characteristics

Included studies were published between 1982 and 2019 (Table 1). Thirty-eight studies enrolled participants from both men and women [25,26,27,28,29,33,34,35,36,42,43,48,49,53,54,55,56,60,61,62,64,65,67,68,69,70,71,73,74,76,77,78,79,80,81,82,83,84], three studies included only female participants [50,51,72], and ten studies included only male participants [30,39,41,52,57,58,59,63,66,75]. Two studies did not report the gender of their participants [31,40].

The majority of the studies included participants with any smoking status (33 studies) [25,27,28,29,34,35,36,42,43,48,49,50,51,52,53,54,55,56,60,61,62,64,65,68,69,70,71,75,78,79,80,82,84]. Eight studies included active smokers exclusively [41,57,58,59,66,67,76,83] and three studies only included participants who had never been smokers [63,72,77]. Nine studies did not report the smoking status of their participants [26,30,31,33,39,40,73,74,81].

Most of the studies were conducted in Asia (22 studies) [26,27,28,29,31,36,41,52,58,59,60,63,64,66,67,72,75,76,77,78,81,82], followed by Europe (18 studies) [30,35,42,43,48,49,50,51,53,54,55,56,57,61,71,79,80,84] and the USA (7 studies) [34,39,40,62,65,69,83]. Two studies involved patient cohorts from the USA, Europe, Asia and Australia [68,70]. Four studies did not report the location of their study [25,33,73,74].

Serum samples were the most common biological sample collected in the studies (21 studies) [26,27,35,36,42,43,48,53,55,56,57,64,68,69,70,74,79,80,81,83,84], followed by urine (18 studies) [26,29,50,51,52,54,58,59,60,62,63,65,66,67,71,72,75,77], plasma (11 studies) [25,28,30,31,49,56,68,70,73,78,82], blood (2 studies) [61,76], tumor tissue (3 studies) [33,39,41], and exhaled breath (2 studies) [34,40]. Four studies collected more than one type of biological sample in their study: three studies collected both serum and plasma samples [56,68,70] and one study collected both serum and urine samples [26].

### 3.3. Quality of Eligible Studies

The quality score of the included studies ranged from 3 to 9 (mean score 7.0 ± 1.6, Table 2). Most of the studies were of high quality (n = 37, 69.8%), while the remaining studies were mainly of moderate quality (n = 15, 28.3%), with one being of low quality (n = 1, 1.9%).

### 3.4. Association between Metabolites and Lung Cancer Risk

A total of 43 individual metabolites and 10 groups of metabolites were reported across all the identified categorical studies (Table S2). Of these, 18 meta-analyses were performed on 12 metabolites, which were: 2-hydroxyethyl mercapturic acid (HEMA), 3-hydroxypropyl mercapturic acid (HPMA), 4-hydroxybut-2-yl mercapturic acid (HBMA), cotinine, creatine riboside, cortisol sulfate, folate, methionine, N-acetylneuraminic acid (NANA), pyridoxal 5′-phosphate, r-1,t-2,3,c-4-tetrahydroxy-1,2,3,4-tetrahydrophenanthrene (PheT) and S-phenyl mercapturic acid (SPMA); and five groups of metabolites, which were:

- Total 3-hydroxycotinine (3-HC) (defined as the sum of concentrations of 3-HC and its glucuronide),
- Total cotinine (defined as the sum of concentrations of cotinine and its glucuronide),
- Total nicotine (defined as the sum of concentrations of nicotine and its glucuronide),
- Total NNAL (defined as the sum of concentrations of 4-(methylnitrosamino)-1-(3-pyridyl)-1-butanol (NNAL) and its glucuronides), and,
- Total nicotine equivalent (TNE) (defined as the sum of the concentration of nicotine, cotinine, 3-HC and their respective glucuronides).

Twelve meta-analyses were performed using the fixed-effects model, while the other six were performed using the random-effects model. Meta-analyses were not performed on the remaining metabolites as they were solely reported in individual studies (Table S3).

Of the serum/plasma metabolites that were meta-analyzed, an increased concentration of cotinine (OR = 14.19, 95% CI = 2.92 to 69.00, Table 3 and Figure S1a) and decreased concentration of folate (OR = 0.82, 95% CI = 0.72 to 0.94, Table 3 and Figure S1b) were significantly associated with increased lung cancer risk. 

Among urinary metabolites that were analyzed, increased concentration of creatine riboside (OR = 3.30, 95% CI = 1.33 to 8.15, Table 3 and Figure S1c), NANA (OR = 2.01, 95% CI = 1.49 to 2.72, Table 3 and Figure S1d), PheT (OR = 2.49, 95% CI = 1.53 to 4.05, Table 3 and Figure S1e), total 3-HC (OR = 3.71, 95% CI = 2.41 to 5.72, Table 3 and Figure S1f), total cotinine (OR = 3.53, 95% CI = 2.62 to 4.77, Table 3 and Figure S1g), total nicotine (OR = 2.51, 95% CI = 1.71 to 3.70, Table 3 and Figure S1h), total NNAL (OR = 2.17, 95% CI = 1.63 to 2.89, Table 3 and Figure S1i) and TNE (OR = 3.75, 95% CI = 2.45 to 5.73, Table 3 and Figure S1j) were significantly associated with increased lung cancer risk.

No statistically significant associations between the exposure of eight metabolites (plasma/serum pyridoxal 5′-phosphate and methionine, and urinary cortisol sulfate, cotinine, HBMA, HEMA, HPMA and SPMA) and lung cancer risk were observed (Figure S3).

Results were stratified by gender for cotinine, pyridoxal 5′-phosphate, total 3-HC, total cotinine, total nicotine, total NNAL and TNE (Figures S1f–j and S4a–c), and by smoking status for cotinine, folate, methionine and pyridoxal 5′-phosphate (Figure S4d–h). No clear trend between men and women were observed with respect to the association between metabolites and lung cancer risk. Among the stratified results, active or former smokers have a stronger association between cotinine, folate and pyridoxal 5′-phosphate exposure and lung cancer risk, when compared to passive or never smokers. 

Cotinine exposure was not significantly associated with lung cancer risk among never smokers (serum/plasma cotinine: OR = 1.06, 95% CI = 0.52 to 2.14, Figure S4d) and passive smokers (urinary cotinine: OR = 2.40, 95% CI = 0.70 to 8.30, Figure S4h), although it remained significant for active smokers (serum/plasma cotinine: OR = 4.15, 95% CI = 2.59 to 6.66, Figure S4d; urinary cotinine: OR = 9.80, 95% CI = 4.50 to 21.30, Figure S4h). The inverse association between serum/plasma folate levels and lung cancer risk was stronger among active smokers (OR = 0.75, 95% CI = 0.42 to 1.32) and former smokers (OR = 0.64, 95% CI = 0.51 to 0.80) when compared with never smokers (OR = 0.86, 95% CI = 0.65 to 1.13) (Figure S4e). The inverse association between serum/plasma pyridoxal 5′-phosphate and lung cancer risk was statistically significant among active smokers (OR = 0.77, 95% CI = 0.65 to 0.92) and former smokers (OR = 0.63, 95% CI = 0.45 to 0.89), but not among never smokers (OR = 0.78, 95% CI = 0.20 to 3.09) (Figure S4g).

### 3.5. Quantitative Difference in Metabolite Level between Lung Cancer Patients and Controls

A total of 255 metabolites were reported across all the identified concentration studies (Table S4). Of these, 40 meta-analyses were performed on 21 types of amino acid in plasma, 11 types of amino acid in serum and eight types of carnitines in serum. Two meta-analyses were performed using the fixed-effects model, while the other 38 were performed using the random-effects model. The remaining metabolites were reported in only individual studies and thus were not meta-analyzed (Table S5).

Of the meta-analyzed metabolites, lower concentration of plasma methionine (WMD = −2.04, 95% CI = −4.01 to −0.06, Table 4 and Figure S2a) and tryptophan (WMD = −6.85, 95% CI = −11.07 to −2.63, Table 4 and Figure S2b) and higher concentration of plasma proline (WMD = 15.98, 95% CI = 6.59 to 25.37, Table 4 and Figure S2c) were found in lung cancer patients, compared with healthy controls. The differences in concentration of other amino acids and carnitines between lung cancer patients and controls were not statistically significant (Figure S5).

## 4. Discussion

### 4.1. Overview

This systematic literature review and meta-analysis aimed to provide a review of the current understanding of the association between metabolites and lung cancer risk. We had included 53 studies, involving 297 metabolites across six different types of biological samples. Although many metabolomics studies on lung cancer were included in this review, the data on any given metabolite is limited, due to the differences in the research focus of each study. Thus, this discussion will primarily focus on the metabolites that were meta-analyzed.

Meta-analyses were performed on 45 metabolites. Our findings indicated that three amino acids (methionine, tryptophan and proline), two smoking-related metabolites (cotinine and PheT) and five groups of smoking-related metabolites (total 3-HC, total cotinine, total nicotine, total NNAL and TNE), a vitamin (folate), a sialic acid (NANA), and a novel compound (creatine riboside) had statistically significant association with lung cancer risk.

### 4.2. Amino Acids

Amino acids have been reported to be associated with other types of cancer in earlier reviews [88,89,90,91]. The amino acids identified in our meta-analyses (methionine, tryptophan and proline) were identified in previous reviews, although the direction of association is different for proline (i.e., positive association in our meta-analysis on lung cancer but inverse association for other cancer types) [88,90,91]. A possible explanation for the discrepancy is that different cancer types have dissimilar metabolism, therefore the association between the patient’s host protein metabolism and amino acid levels with cancer risk may differ between cancer types [92,93,94,95].

#### 4.2.1. Methionine

Results from concentration studies (Figure S2a) suggested that plasma methionine concentration was lower in lung cancer patients. The findings from Kami et al. [33] also identified that the concentration of methionine was increased in tumor tissue of lung cancer patients (Table S5). These observations are largely consistent with the current understanding of the roles that methionine plays in cancer cell proliferation.

The influential roles of methionine in cancer cell growth has been well-documented [96,97,98,99]. Methionine is involved in several critical activities in cancer cells, such as nucleotide biosynthesis via the one-carbon metabolism pathway [100,101,102] and protein synthesis [103]. Methionine is also a precursor metabolite of S-adenosylmethionine, a co-substrate involved in methyl group donation, for cellular processes such as epigenetic control and protein methylation [100,104,105]. Previous reviews have identified that the DNA methylation patterns in lung cancer were altered, where genome-wide hypomethylation, and hypermethylation of the promoter regions of several tumor suppressor genes, such as *DAPK*, *RASSF1A* and *RARβ*, were commonly observed [106,107]. It is thus unsurprising to observe an increased demand for methionine in cancer cells, given the multi-faceted role it plays in oncogenesis. Consequently, cancer cells increase its uptake of essential amino acids, such as methionine, through the up-regulation of essential amino acid transporters [108,109,110].

Methionine is also involved in glutathione formation, which serves as an antioxidant [111]. Lowered glutathione level is a biomarker of oxidative stress [112], and may contribute to chronic inflammation and cancer development [113]. These observations suggest that lowered methionine levels may also play a role in the precipitation of cancer cell proliferation, through the reduction in antioxidant capacity. Methionine is also a direct target of reactive oxygen species (ROS) and acts as a ROS scavenger [114,115].

#### 4.2.2. Tryptophan

Similar to methionine, our findings indicated that the plasma levels of tryptophan were lowered in lung cancer patients (Figure S2b). Furthermore, findings from Chuang et al. [61] suggested that increased kynurenine and 3-hydroxykynurenine levels were associated with lung cancer risk, although the association was not statistically significant (Table S3). These observations could be explained by a potential mechanism that cancer cells use to bypass detection by the host immune system [116].

Tryptophan is a precursor molecule in the kynurenine pathway [117], which synthesizes several metabolites with immunosuppressive activity. The metabolites then suppresses T-cell proliferation and alters NK cell function [118]. A review by Heng et al. [119] identified that increased expression of indoleamine-2,3-dioxygenase 1 (*IDO1*), an enzyme involved in the synthesis of kynurenine from tryptophan, is positively correlated with poorer cancer prognosis across different cancers, including lung cancer. They further illustrated that an increase in *IDO1* would up-regulate the production of kynurenine and its metabolites, which were then used to suppress T-cell activity.

#### 4.2.3. Proline

Our findings indicated that the plasma levels of proline were higher among lung cancer patients (Figure S2c). Unlike methionine and tryptophan, the biochemical rationale behind the increase in plasma concentrations of proline is unclear.

The role of proline in lung carcinogenesis is an area of active research. A recent review by Phang et al. provided an abridged overview of the functionality of proline in cancer cells, as a source for cellular energy production and as an intermediate between the urea cycle and Krebs cycle [120]. Several studies have identified that overexpression of proline dehydrogenase, the enzyme involved in proline degradation, promotes cancer progression [121,122,123]. Future studies could explore the relationship between increased circulating proline concentrations and lung cancer risk.

### 4.3. Folate

Folate is a metabolite in the folate cycle, which is part of the one-carbon metabolism pathway [100,101,102]. Folate is coupled with the methionine cycle as one of its metabolites, 5-methyltetrahydrofolate, is involved in the regeneration of methionine from homocysteine [100,101,102]. Similar to methionine, folate is involved in nucleotide biosynthesis. 10-formyltetrahydrofolate, a metabolite in the folate cycle, serves as a formyl group donor for the formation of carbon-2 and carbon-8 of the purine ring in de novo purine synthesis [100,101,102]. 

Our stratified analysis has identified that active and former smokers have a stronger inverse association between folate levels and lung cancer risk when compared with never smokers (Figure S4e). A possible explanation for this observation may be the confounding effect of exposure to tobacco smoke. The authors of the respective studies cautioned that despite adjusting for the cotinine level, the results may still be confounded by smoking status, considering the influence that smoking has on lung cancer risk [55,68].

### 4.4. Smoking-Related Metabolites

Smoking is well-established as a significant risk factor for lung cancer [124,125,126,127]. More than 5000 compounds were identified in tobacco smoke, among which, over 70 compounds were identified as carcinogenic [128,129,130]. In this study, we conducted meta-analyses on nine smoking-related metabolites, namely nicotine, cotinine, 3-HC, PheT, NNAL, HBMA, HEMA, HPMA and SPMA, either as single metabolite, or as metabolite groups (as are the cases for the meta-analyses on total nicotine, total cotinine, total 3-HC, total NNAL and TNE).

#### 4.4.1. Nicotine and Cotinine

Cotinine is a major metabolite from nicotine metabolism in humans, accounting for 70–80% of the metabolites formed [131]. Cotinine then undergo further metabolism before being excreted [132], with 3-HC being a main metabolite found in urine [133]. Although nicotine and cotinine are associated with lung cancer risk, it is imperative to note that neither are carcinogens. Several studies have shown that nicotine and cotinine neither induced nor influenced lung tumorigenesis [134,135].

Results from our meta-analyses suggested that increased serum/plasma cotinine, urinary total 3-HC, cotinine and nicotine exposure were significantly associated with higher lung cancer risk (Figure S1a,f–h). Urinary cotinine was also associated with higher lung cancer risk, albeit not statistically significant (Figure S3d). These observed associations were noteworthy as nicotine exposure is a consequence of cigarette smoke exposure, which itself contains carcinogens that may induce tumorigenesis [128,129,130].

From the stratified analysis by smoking status, cotinine exposure was associated with lung cancer risk among active smokers, but not among passive or never smokers (Figure S4d,h). Our meta-analyses have also identified that urinary TNE, a biomarker that demonstrated high correlation with smoking [136,137], is positively associated with lung cancer risk (Figure S1j). These results further exemplified the significance of smoking, rather than nicotine exposure, as a risk factor for lung cancer.

#### 4.4.2. PheT

Polycyclic aromatic hydrocarbons (PAHs), are a class of compounds that are well-established as carcinogens [138], with well-documented association between PAH exposure and lung cancer risk [139,140]. Of these, benzoapyrene (BaP), a compound known for its carcinogenic effects, was frequently studied and often used as a reference compound for the evaluation of carcinogenicity of other PAHs [141]. The International Agency for Research on Cancer (IARC) classified several PAHs as carcinogens or potential carcinogens [142]. PAHs are pro-carcinogens [143,144,145,146], with its carcinogenicity potentiated by multi-step biotransformation of the parent compounds, through several metabolic pathways, such as CYP1A1/1B1 and epoxide hydrolase pathway, and aldo-keto reductases pathway [138]. Particularly, PAHs were activated through the formation of bay-region diol epoxides [147]. The carcinogenic metabolites then form DNA adducts, causing errors in DNA replication and altered epigenetic controls, and may contribute to carcinogenesis [143,144,145,146].

PheT, a metabolite of non-carcinogenic phenanthrene, is an established surrogate measure for carcinogenic PAH exposure [147]. Results from our meta-analysis showed that increased PheT exposure was associated with higher lung cancer risk (Figure S1e), consistent with the current understanding of the roles that PAH may play in lung cancer.

#### 4.4.3. NNAL

NNAL is a metabolite of 4-(N-Nitrosomethylamino)-1-(3-pyridyl)-1-butanone (NNK), one of the compounds found in cigarette smoke [148]. NNAL can be further metabolized through glucuronidation into either the O or N glucuronides [149]. Both NNK and NNAL are pro-carcinogens [149]. On its own, both compounds do not have any carcinogenic effect [149]. However, its metabolites (such as α-methylenehydroxy-NNK and 4-(3-pyridyl)-4-oxobutane-1-diazohydroxide) are carcinogenic and may cause DNA damage through the formation of DNA adducts [149].

Total NNAL, the sum of urinary NNAL and its glucuronides, were validated biomarkers for NNK exposure [150]. Our meta-analyses suggested that increased urinary total NNAL was significantly associated with higher lung cancer risk (Figure S1i), further substantiating the current theory of the role of NNAL (and NNK) in carcinogenesis.

#### 4.4.4. HBMA, HEMA, HPMA and SPMA

HBMA, HEMA, HPMA and SPMA are mercapturic acid metabolites of crotonaldehyde, ethylene oxide, acrolein and benzene respectively, and are validated biomarkers for the exposure to the respective parent compounds [151]. Our findings suggested that increased exposure to any of these four metabolites were not associated with lung cancer risk (Figure S3e–h).

Crotonaldehyde is a volatile α,β-unsaturated carbonyl compound [152]. Although crotonaldehyde was shown to induce liver tumors in rats [153], to date, insufficient evidence was found to show that crotonaldehyde exposure is associated with lung cancer risk. According to the IARC, crotonaldehyde is “not classifiable as to its carcinogenicity to humans” [154].

Ethylene oxide is a volatile cyclic ether [155]. The IARC classified ethylene oxide as “carcinogenic to humans”, based on animal data [154]. However, an earlier review suggested that current evidence was insufficient to conclude the carcinogenicity of ethylene oxide on human. Similarly, our meta-analysis did not find any association between ethylene oxide exposure and lung cancer risk (OR = 1.01, 95% CI = 0.64 to 1.59; Figure S3f).

Acrolein is a volatile α,β-unsaturated aldehyde [156]. Although acrolein had demonstrated carcinogenicity in in vitro models [157], there is insufficient evidence to show that acrolein exposure is associated with lung cancer risk to date. According to the IARC, acrolein is “not classifiable as to its carcinogenicity to humans” [154].

Benzene is a volatile aromatic compound [155]. An earlier review summarized the carcinogenic effects of benzene for different tumors in animals [158]. A review by IARC summarized that benzene exposure was associated with increased lung cancer risk in a few studies, although most studies have shown no association [159]. Similarly, our study suggested that increased exposure to SPMA is associated to higher lung cancer risk, albeit not statistically significant (OR = 1.28, 95% CI = 0.83 to 1.96; Figure S3h). The IARC classified benzene as “carcinogenic to humans”, due to its association with several types of leukemia and lymphoma [154,159].

### 4.5. NANA

Increased concentrations of NANA have been associated with several cancer types [160,161,162,163,164,165]. NANA is used in the formation of glycans [166], which in turn play several influential roles in cells, such as inducing proper folding of newly synthesized proteins [167], cell signaling and adhesion [168].

Anomalous glycosylation is increasingly recognized as a hallmark of cancer [169]. Tumor cells are known to produce increased amounts of glycans on the plasma membrane [170]. Excessive glycosylation increases the negative charge on plasma membrane of tumor cells, promoting cell detachment and encouraging metastasis [171].

Results from our meta-analysis concluded that increased NANA exposure was associated with higher lung cancer risk (Figure S1d). Notably, the observation of increased NANA may be due to increased turnover and shedding of cancer cells, resulting in glycans being released into the serum [166].

### 4.6. Creatine Riboside

Creatine riboside is a novel metabolite that was not previously identified in other cancer studies. To date, no studies were performed to elucidate the potential mechanisms behind the elevation of creatine riboside concentration in lung cancer patients. In the study that identified creatine riboside as a statistically significant metabolite, Mathe et al. [62] hypothesized that higher concentrations of creatine riboside may be the consequence of both increased creatine concentrations and higher phosphate turnover within tumor cells.

### 4.7. Strengths and Limitations of This Study

Previous reviews summarized recent work regarding the approaches in the identification of metabolites that could serve as biomarkers for lung cancer and enumerated several potential biomarkers [22,23]. On the other hand, previous meta-analyses focused only on exposure to vitamin D [172,173]. Our current study further expanded on the existing reviews and meta-analyses by identifying other types of metabolites and quantifying either the differences in concentrations between lung cancer patients and controls or the lung cancer risk associated with levels of metabolite. By not restricting the type of metabolite in our inclusion criteria, we included metabolites across different metabolism pathways to provide a comprehensive overview of the current progress in the field.

The results of our meta-analysis should be interpreted with the following caveats in mind. Firstly, most of the metabolites identified were only reported as individual, small-scale studies. The limited quantity of evidence found for most metabolites suggests that further studies are merited in order to validate the feasibility of using these metabolites as biomarkers for lung cancer. Furthermore, about one-third (n = 16, 30.2%) of the included studies were of low-to-moderate quality. Thus, with these points in consideration, the current results should be interpreted with prudence, and future well-designed, large-scale studies are warranted.

Secondly, we could not perform extensive stratified analyses to explore sources of clinical heterogeneity. Due to the limited studies available on each metabolite analyzed, we were only able to perform subgroup analysis by gender and smoking status for those metabolites that were investigated by sufficient number of studies. In addition, most studies did not include the definitions for smoking status, nor did they provide stratified results by smoking status. Thus, we could not account for any possible differences in smoking status. Some of the included studies only presented the combined results of plasma and serum, therefore we were unable to separate them in our analysis [56,68,70]. Furthermore, we could not perform stratified analyses by histological subtype, stage of lung cancer and study design. Although the majority of studies reported the histological subtype of lung cancer patients (Table 1), most studies did not report stratified results based on lung cancer subtypes. Moreover, only a handful of studies reported the lung cancer stage. Several reviews on other cancers, such as colorectal cancer and gastric cancer, have identified that the metabolome profile is different between early-stage and late-stage cancer [88,90]. Thus, future studies would benefit by exploring the influence of lung cancer stage on the evaluated associations in relation to different metabolites.

We noted some heterogeneity in the identified studies. The heterogeneity may be attributed to factors such as ethnicity and smoking status. We recognize that this is a knowledge gap in the current state of research, and more studies should be performed to facilitate the identification of possible factors for heterogeneity. The method used for metabolite identification may also be a possible source of heterogeneity. Considering that studies used different analytical platforms and methodology to identify and quantify the metabolites (Table 1), more work should be done to further harmonize the workflow adopted by researchers in metabolite quantification. We recommend researchers to follow the best practices for the quantification of the metabolites, such as that proposed by Lu et al. [174], and adopt minimum reporting standards, such as that proposed by the Metabolomics Standards Initiative [175], to facilitate the replication of studies and allowing for more meaningful comparisons between different studies.

Thirdly, we were unable to ascertain if the patients in the identified studies were free of co-morbidities, or if it was adjusted for. Several co-morbidities, such as chronic obstructive pulmonary disease (COPD), diabetes mellitus (DM) and other malignancies, were identified to be more prevalent in lung cancer patients compared to the general population [176,177,178]. The metabolomic profile of patients in disease states, such as COPD [179] and DM [180], were identified to be different from that of a normal population. While we excluded studies involving patients with other malignancies or having a history of any malignancy, we could not ascertain if patients were free of co-morbidities at the time of sample collection. It is beneficial for future studies to consider the participants’ co-morbidities as a criterion for recruitment, or to report it as part of the baseline characteristics of their participants.

## 5. Conclusions

This review identified several metabolites that are significantly associated with lung cancer. Amino acids, smoking-related metabolites, folate, NANA and creatine riboside warrant further investigation for use as potential biomarkers. Although a substantial number of studies were reviewed, meta-analyses could only be performed on a subset of the identified metabolites, as most metabolites were solely studied in individual studies. Out of the analyzed metabolites, plasma samples of amino acids may hold more promise. Further studies are warranted to elucidate the possible link between amino acid levels and lung cancer, and to validate the use of these metabolites as potential biomarkers of lung cancer in a larger population.

## Figures and Tables

**Table 1:** Characteristics of included studies (n = 53).

Reference, Year	No. of Study Participants (Case/Control)	Type of Lung Cancer Cases (n)	Type of Biological Sample	Method Used for Metabolite Identification	Level of Identification ^g^	Age of Participants at Recruitment	Gender of Participants	Location of Study (Study Name, if Applicable)	Smoking Status ^a^
Cohort studies reporting the association between exposure to metabolite and lung cancer (n = 4)									
Kilkkinen et al., 2008 [48]	6937 (122 cases)	NR	Serum	Nicotine Metabolite RIA kit	N.A.	50.9 ± 14.9 ^b^	3207M, 3730F	Finland (MFhes)	2052A, 4885N
Afzal et al., 2013 [49]	9791 (507 cases)	NR	Plasma	DiaSorin Liaison 25(OH)D TOTAL assay	N.A.	58 (48–65) ^c^	4359M, 5432F	Copenhagen, Denmark (CCHS)	7474E, 2317N
Ordóñez-Mena et al., 2016 [42]	8928 (134 cases)	NR	Serum	Immunoassay	N.A.	63 (57–67) ^c^	3545M, 5383F	Germany (ESTHER cohort)	NR
	4307 (58 cases)	NR	Serum	Immunoassay	N.A.	62 (56–68) ^c^	1553M, 2754F	Norway (TROMSØ cohort)	N/F
Gao et al., 2019a [43]	4345 (39 cases)	NR	Serum	d-ROM assay	N.A.	69 (64–74) ^c^	1966M, 2379F	Germany (ESTHER cohort)	335A, 1552F, 2374N, 84U
	221/1000 (case-cohort study)	NR	Serum	d-ROM assay	N.A.	Ca: 51 (44–56) ^c^ Cohort: 42 (37–50) ^c^	Ca: 150M, 71F Cohort: 548M, 452F	Norway (TROMSØ cohort)	Ca: 181A, 28F, 11N, 1U Cohort: 443A, 245F, 310N, 2U
Case-control studies reporting the association between exposure to metabolite and lung cancer (n = 23)									
de Waard et al., 1995 [50]	92/305	NR	12H Urine	Capillary gas chromatography-mass spectrometry	N.A.	40–64 ^d^	F	Utrecht, Netherlands (DOM Project)	Ca: 69A, 23P Co: 257A, 191P
Ellard et al., 1995 [51]	69/255	NR	12H Urine	Automated colorimetric method – automated versions of the manual direct barbituric acid and alkaline picrate	N.A.	40–64 ^d^	F	Utrecht, Netherlands (DOM Project)	Ca: 48A, 21N Co: 58A, 197P
London et al., 2000 [52]	232/710	NR	Urine	HPLC	N.A.	58.8 ± 4.8 ^b^	M	Shanghai, China (SCS)	Ca: 189A, 19F, 24N Co: 337A, 58F, 315N
Boffetta et al., 2006 [53]	1741/1741	NR	Serum	Qualitative immunoassay	N.A.	Adults (Actual age range reported as categorical data)	Ca: 1322M, 419F Co: 1322M, 419F	Norway	Ca: 1393A, 96E, 128F, 53N, 71U Co: 727A, 67E, 411F, 445N, 91U
Loft et al., 2007 [54]	251/261	AC (81) SCLC (55) SCC (51) Others (34)	Urine	HPLC	N.A.	50–64 ^d^	Ca: 138M, 113F Co: 146M, 115F	Denmark (DCH Study)	In total, 399A, 94F, 15N
Johansson et al., 2010 [55]	899/1815	SCLC (110) AC (272) LCC (50) SCC (200) Others (267)	Serum	LC-MS/MS, GC-MS/MS and microbiological assay	N.A.	59 (43–73) ^e^	Ca: 559M, 340F Co: 1126M, 689F	Europe (EPIC Study)	Ca: 529A, 260F, 96N, 14U Co: 413A, 663F, 707N, 32U
Timofeeva et al., 2011 [56]	894/1805	SCLC (108) AC (270) LCC (50) SCC (199) Others/Unknown (267)	Plasma/Serum	LC-MS/MS	N.A.	Adults (Actual age range reported as categorical data)	Ca: 556M, 338F Co: 1117M, 688F	Europe (EPIC Study)	Ca: 526A, 258F, 96N Co: 409A, 659F, 705N
Weinstein et al., 2011 [57]	500/500	SCLC (100) SCC (179) AC (73) Others (148)	Fasting serum	DiaSorin Liaison 25(OH)D TOTAL assay	N.A.	59 (55–62) ^c^	M	Finland (ATBC)	A
Yuan et al., 2011 [58]	476/476	AC (105) SCC (153) SCLC (22) Others (35) Unknown (161)	Urine	LC-MS/MS, GC-MS/MS	N.A.	Ca: 57.4 ± 5.0 ^b^ Co: 57.2 ± 4.9 ^b^	M	Shanghai, China (SCS)	A
Yuan et al., 2012 [59]	343/392	AC (70) SCC (104) SCLC (22) Others (28) Unknown (119)	Urine	LC-MS/MS, GC-MS/MS	N.A.	NR	M	Shanghai, China (SCS)	A
Eom et al., 2013 [60]	35/140	NR	Urine	HPLC	N.A.	Ca: 68.87 ± 6.86 ^b^ Co: 68.86 ± 6.85 ^b^	Ca: 27M, 8F Co: 108M, 32F	South Korea (KMCC)	Ca: 28A/F, 7N Co: 91A/F, 48N
Chuang et al., 2014 [61]	893/1748	SCLC (140) AC (284) LCC (63) SCC(198) Others (208)	Blood	LC-MS/MS, GC-MS/MS	N.A.	59 (42–72) ^e^	Ca: 556M, 337F Co: 1086M, 662F	Europe (EPIC Study)	Ca: 526A, 257F, 96N, 14U Co: 396A, 648F, 674N, 30U
Mathe et al., 2014 [62]	469/536	NSCLC	Urine	UPLC-ESI-QTOFMS	Level 1	Ca: 66.2 ^f^ Co: 66.6 ^f^	Ca: 237M, 232F Co: 276M, 260F	Greater Baltimore, Maryland, USA	Ca: 222A, 214F, 33N Co: 71A, 249F, 216N
Yuan et al., 2014 [63]	82/83	SCC (16) AC (34) SCLC (2) Others (9) Unknown (21)	Urine	LC-MS/MS	N.A.	Ca: 58.1 ± 5.2 ^b^ Co: 58.0 ± 5.4 ^b^	M	Shanghai, China (SCS)	N
Wang et al., 2015 [64]	100/100	SCC (35) AC (51) Others (14)	Serum	LC-MS/MS and HPLC	N.A.	Ca: 57.1 ± 9.2 ^b^ Co: 56.6 ± 9.2 ^b^	Ca: 52M, 48F Co: 51M, 49F	Changchun, Jilin, China	Ca: 48A, 20F, 32N Co: 9A, 35F, 56N
Haznadar et al., 2016 [65]	178/351	AC (59) SCC (36) NSCLC (19) SCLC (29) LCC (9) Others (13) Unknown (13)	Urine	UPLC-MS	Level 1	Ca: 57.7 ± 8.6 ^b^ Co: 57.3 ± 8.5 ^b^	Ca: 101M, 77F Co: 194M, 152F	South-eastern states, USA (SCCS)	Ca: 127A, 39F, 7N Co: 140A, 99F, 97N
Yuan et al., 2016 [66]	325/356	SCC (102) AC (80) SCLC (15) Others (17) Unknown (111)	Urine	LC-MS/MS, GC-MS/MS	N.A.	Ca: 56.7 ± 4.9 ^b^ Co: 56.7 ± 4.9 ^b^	M	Shanghai, China (SCS)	A
Yuan et al., 2017 [67]	197/197	AC (51) SCC (48) SCLC (25) Others (49) Unknown (24)	Urine	LC-MS/MS, GC-MS/MS	N.A.	60.8 ± 6.2 ^b^	Ca: 165M, 32F Co: 164M, 33F	Singapore (SCHS)	A
Fanidi et al., 2018 [68]	5364/5364	LCC (174) SCLC (492) SCC (836) AC (2056) Others/Unknown (1806)	Plasma/Serum	LC-MS/MS, GC-MS/MS and microbiological assay	N.A.	60 (44–72) ^e^	2908M, 2456F	Europe, Australia, China, Singapore, USA (LC3)	2519A, 1518F, 1327N
Haznadar et al., 2018 [69]	406/437	AC (202) SCC (108) NSCLC (96)	Serum	UPLC-MS	N.A.	Ca: 66.3 ± 10.0 ^b^ Co: 67.0 ± 8.9 ^b^	Ca: 214M, 192F Co: 234M, 203F	Baltimore, Maryland, USA	Ca: 191A, 186F, 29N Co: 52A, 209F, 176N
Larose et al., 2018 [70]	5364/5364	LCC (174) SCLC (492) SCC (836) AC (2056) Others/Unknown (1806)	Plasma/Serum	LC-MS/MS	N.A.	60 (44–72) ^e^	2908M, 2456F	Europe, Australia, China, Singapore, USA (LC3)	2519A, 1518F, 1327N
Gao et al., 2019b [71]	245/735	NR	Urine	Nitrite/nitrate colorimetric assay	N.A.	62 (59–68) ^c^	Ca: 170M, 75F Co: 509M, 226F	Germany (ESTHER cohort)	Ca: 124A, 87F, 29N Co: 365A, 260F, 93N
Seow et al., 2019 [72]	275/289	AC (135) SCC (9) Others (19) Unknown (112)	Urine	UPLC-MS and 600-MHz hydrogen 1 NMR	Level 2	Ca: 61 (52–65) ^c^ Co: 62 (53–66) ^c^	F	Shanghai, China (SWHS)	N
Studies reporting the concentration of metabolites in lung cancer patients and controls (n = 24)									
Kukreja et al., 1982 [39]	14 (Self-controlled)	SCC (8) AC (4) LCC (2)	Tumor tissue	Silicic acid chromatography and radioimmunoassay	N.A.	NR	M	Chicago, Illinois, USA	NR
Hendrick et al., 1988 [73]	29/18	AC/LCC (11) SCC (9) SCLC (8)	Plasma	Radioimmunoassay	N.A.	Ca: 65.4 ± 7.0 ^b^ Co: 65.5 ± 10.2 ^b^	Ca: 20M, 9F Co: 11M, 7F	NR	NR
Preti et al., 1988 [34]	10/8	SCC (6) LCC (2)	Exhaled breath	GC-MS	Level 1	Ca: 54–77 ^d^ Co: 57–66 ^d^	Ca: 7M, 3F Co: 4M, 4F	Pennsylvania, USA	Ca: 4A, 6F Co: 4A, 1F, 3N
Proenza et al., 2003 [30]	14/14	NR	Fasting plasma	HPLC	N.A.	Ca: 64.4 ± 6.0 ^b^ Co:59.8 ± 7.9 ^b^	M	Spain	NR
Masri et al., 2005 [40]	11/35	NR	Exhaled breath	Chemiluminescent analyzer, amperometric sensor	N.A.	NR	NR	Cleaveland, Ohio, USA	NR
Gencer et al., 2006 [74]	38/26	EC (14) SCLC (12) AC (12)	Fasting serum	Technicon RA-XT^®^ autoanalyzer	N.A.	AC: 54 ± 12 ^b^ EC: 59.6 ± 14 ^b^ SCLC: 52 ± 9 ^b^ Co: 53.2 ± 12 ^b^	AC: 9M, 3F EC: 11M, 3F SCLC: 10M, 2F Co: 21M, 5F	NR	NR
Zhang et al., 2006 [75]	10/12	NR	24H Urine	HPLC and GC-MS/MS	N.A.	30–70 ^d^	M	Beijing, China	Ca: 2A, 8F Co: 8A, 4N
Esme et al., 2008 [76]	49/20	AC (24) SCC (21) LCC (4)	Blood	Spectrophotometric method	N.A.	Ca: 57.2 ± 10.1 ^b^ Co: 52.1 ± 12.1 ^b^	Ca: 40M, 9F Co: 9M, 11F	Turkey	A
Hu et al., 2009 [26]	30/63	NSCLC	Non-fasting serum & Urine	HPLC, amino acid analyzer	N.A.	Ca: 59.7 ± 8.0 ^b^ Co: 67.0 ± 5.4 ^b^	Ca: 7M, 3F Co: 4M, 4F	Anhui, China	NR
Miyagi et al., 2011 [28]	200/996	AC (133) SCC (35) SCLC (8) Others (9) Unknown (15)	Fasting plasma	HPLC–electrospray ionization mass spectrometry	N.A.	Ca: 65.0 ± 10.0 ^b^ Co: 63.2 ± 9.2 ^b^	Ca: 125M, 75F Co: 635M, 371F	Japan	Ca: 84A, 54F, 60N, 2U Co: 137A, 245F, 536N, 78U
Kami et al., 2013 [33]	9 (Self-controlled)	AC (3) SCC (4) LCC (1) PC (1)	Tumor tissue	Capillary electrophoresis time-of-flight mass spectrometry	Level 2	56–82 ^d^	8M, 1F	NR	NR
Okur et al., 2013 [41]	15 (Self-controlled)	AC (3) EC (12)	Tumor tissue	Chemiluminescence assay	N.A.	63.6 ± 9.2 ^b^	M	Istanbul, Turkey	A
Shingyogi et al., 2013 [27]	86/323^h^	AC (55) SCC (12) Other NSCLC (8) SCLC (11) Unknown (3)	Fasting serum	HPLC–electrospray ionization mass spectrometry	N.A.	Ca: 67.8 ± 8.2 ^b^ Co: 61.9 ± 6.0 ^b^	Ca: 68M, 18F Co: 263M, 60F	Japan	Ca: 29A, 36F, 18N, 3U Co: 62A, 107F, 139N, 15U
Hwang et al., 2014 [77]	74/85	NSCLC	Urine	LC-MS	N.A.	Ca: 64.0 ± 10.3 ^b^ Co: 55.5 ± 7.2 ^b^	Ca: 45M, 29F Co: 23M, 62F	Goyang, South Korea	N
Kim et al., 2015 [78]	75/80	AC (37) SCC (30) Other NSCLC (4) Unknown (1)	Fasting plasma	HPLC–electrospray ionization mass spectrometry	N.A.	Ca: 65.6 ± 9.2 ^b^ Co: 63.2 ± 8.9 ^b^	Ca: 51M, 21F Co: 44M, 26F	South Korea	Ca: 40A, 13F, 19N Co: 8A, 25F, 34N, 3U
Klupczynska et al., 2016a [79]	90/62	AC (40) SCC (50)	Fasting serum	LC–MS/MS	N.A.	Ca: 64 ± 6.9 ^b^ Co: 62 ± 8.8 ^b^	Ca: 58M, 32F Co: 40M, 22F	Poznan, Poland	Ca: 43A, 46N, 1U Co: 11A, 49N, 3U
Klupczynska et al., 2016b [80]	90/63	AC (40) SCC (50)	Fasting serum	LC–MS/MS	N.A.	Ca: 64 ± 6.9 ^b^ Co: 62 ± 8.7 ^b^	Ca: 58M, 32F Co: 41M, 22F	Poznan, Poland	Ca: 43A, 46N, 1U Co: 11A, 49N, 4U
Ni et al., 2016 [81]	40/100	NR	Serum	LC–MS/MS	N.A.	Ca: 51–83 ^d^ Co: NR	Ca: 26M, 14F Co: NR	Beijing, China	NR
Yue et al., 2018 [31]	20/20	SCLC	Fasting plasma	LC–MS/MS	Levels 1, 2 (for different metabolites)	NR	NR	Beijing, China	NR
Kawamoto et al., 2019 [29]	54/124	AC	Urine	Radioimmunoassay	N.A.	Ca: 66.6 ± 10.0 ^b^ Co: 44.2 ± 12.9 ^b^	Ca: 23M, 31F Co: 52M, 72F	Tokyo, Japan	Ca: 30A/F, 24N Co: 124N
Klupczynska et al., 2019 [35]	20/20	AC (9) SCC (11)	Fasting serum	Triple quadrupole tandem mass spectrometer coupled with HPLC	N.A.	Ca: 62 ± 5 ^b^ Co: 63 ± 6 ^b^	Ca: 11M, 9F Co: 8M, 12F	Poznan, Poland	Ca: 12A, 8F/N/U Co: 6A, 14F/N/U
Ni et al., 2019 [36]	17/30	AC (4) SCC (5) SCLC (5) Other NSCLC (3)	Fasting serum	LC–MS/MS	N.A.	Ca: 53–77 ^d^ Co: 34–85 ^d^	Ca: 13M, 4F Co: 23M, 7F	Beijing, China	Ca: 4A, 5F, 8N Co: 7A, 6F, 16N, 1U
Pietzke et al., 2019 [25]	56/50	AC (31) SCC (20)	Fasting plasma	GC-MS, LC-MS	N.A.	Ca: 66 ± 9 ^b^ Co: 48 ± 14 ^b^	Ca: 49M, 7F Co: NR	NR	Ca: 28A, 28F/N/U Co: NR
Zhang et al., 2019 [82]	28/38	NR	Fasting plasma	HPLC-MS/MS	N.A.	Ca: 30–79 ^d^ Co: 20–79 ^d^	Ca: 23M, 5F Co: 20M, 18F	Shenyang, China	Ca: 21A, 7N Co: 15A, 8N, 15U
Studies reporting both the association between exposure to metabolite and lung cancer and the concentration of metabolites in lung cancer patients and controls (n = 2)									
Church et al., 2009 [83]	100/100	NR	Non-fasting serum	GC-MS	N.A.	55–74 ^d^	Ca: 71M, 29F Co: 64M, 36F	USA (PLCO)	A
Skaaby et al., 2014 [84]	12204 (126 cases)	NR	Serum	HPLC, immunoassay, IDS-SYS 25-Hydroxy Vitamin D method	N.A.	18–71 ^d^	5866M, 6338F	Denmark (Monica10, Inter99, Health2006)	4554A, 3401F, 4249N

*AC* Adenocarcinoma, *ATBC* Alpha-Tocopherol, Beta-Carotene Cancer Prevention, *Ca* Cases, *CCHS* Copenhagen City Heart Study, *Co* Controls, *DCH* Diet, Cancer and Health, *DOM* Diagnostisch Onderzoek (investigation) Mammacarcinoom, *EC* Epidermoid carcinoma, *EPIC* European Prospective Investigation into Cancer and Nutrition, *ESTHER* Epidemiologische Studie zu Chancen der Verhütung, Früherkennung und optimierten Therapie chronischer Erkrankungen in der älteren Bevölkerung, *GC-MS/MS* Gas chromatography–tandem mass spectrometry, *KMCC* Korean Multi-center Cancer Cohort, *LC3* Lung Cancer Cohort Consortium, *LCC* Large cell carcinoma, *LC-MS/MS* Liquid chromatography–tandem mass spectrometry, *MFhes* Mini-Finland Health Survey, *NMR* Nuclear magnetic resonance, *NR* Not reported, *NSCLC* Non-small cell lung cancer, *PC* Pleomorphic carcinoma, *PLCO* Prostate, Lung, Colorectal, and Ovarian Cancer Screening Trial, *PPP* Pomeranian Pilot Lung Cancer Screening Programme, *SCC* Squamous cell carcinoma, *SCCS* Southern Community Cohort Study, *SCHS* Singapore Chinese Health Study, *SCLC* Small-cell lung cancer, *SCS* Shanghai Cohort Study, *SWHS* Shanghai Women’s Health Study, *UPLC-MS* Ultra-high-performance liquid chromatography–tandem mass spectrometry, *HPLC* High-performance liquid chromatography, *LC-MS/MS* Liquid chromatography-tandem mass spectrometry, *GC-MS* Gas chromatography-mass spectrometry, *GC-MS/MS* Gas chromatography-tandem mass spectrometry, *UPLC-ESI-QTOF-MS* Ultra-performance liquid chromatography-electrospray-ionization-quadrupole time-of-flight mass spectrometry, *N.A.* not applicable. ^a^ Abbreviations for smoking status are as follows: *A*, Active smoker; *E*, Ever smoker (active or former smoker); *F*, Former smoker; *N*, Never smoker; *P*, Passive smoker; *U*, Unknown smoking status. ^b^ Data reported as mean ± SD. ^c^ Data reported as median and interquartile range. ^d^ Data reported as range (minimum-maximum). ^e^ Data reported as median, 5th percentile and 95th percentile. ^f^ Data reported as mean. ^g^ For untargeted metabolomics data: Level 1 identifies the compound by confirming with an authentic standard; Level 2 is matching to databases.

**Table 2:** Methodological quality assessment of included studies (n = 53) using a 9-point Newcastle–Ottawa Scale.

Reference, Year.	Selection (4) ^a^	Comparability (2) ^b^	Determination of Exposure/Outcome (3) ^c^	Overall Quality Score
Kukreja et al., 1982 [39]	1	2	3	6.8 ^d^
Hendrick et al., 1988 [73]	3	0	2	5
Preti et al., 1988 [34]	3	0	2	5
de Waard et al., 1995 [50]	4	1	2	7
Ellard et al., 1995 [51]	4	2	2	8
London et al., 2000 [52]	4	1	3	8
Proenza et al., 2003 [30]	2	0	2	4
Masri et al., 2005 [40]	3	2	3	9 ^d^
Boffetta et al., 2006 [53]	4	2	3	9
Gencer et al., 2006 [74]	2	0	2	4
Zhang et al., 2006 [75]	1	0	2	3
Loft et al., 2007 [54]	4	2	2	8
Esme et al., 2008 [76]	3	1	2	6
Kilkkinen et al., 2008 [48]	4	2	2	8
Church et al., 2009 [83]	4	2	2	8
Hu et al., 2009 [26]	3	0	2	5
Johansson et al., 2010 [55]	3	2	3	8
Miyagi et al., 2011 [28]	3	2	2	7
Timofeeva et al., 2011 [56]	4	2	2	8
Weinstein et al., 2011 [57]	4	2	2	8
Yuan et al., 2011 [58]	4	2	3	9
Yuan et al., 2012 [59]	4	2	3	9
Afzal et al., 2013 [49]	4	2	3	9
Eom et al., 2013 [60]	4	2	2	8
Kami et al., 2013 [33]	2	2	3	7.9 ^d^
Okur et al., 2013 [41]	3	2	3	9 ^d^
Shingyogi et al., 2013 [27]	3	0	2	5
Chuang et al., 2014 [61]	3	2	2	7
Hwang et al., 2014 [77]	2	1	2	5
Mathe et al., 2014 [62]	3	2	2	7
Skaaby et al., 2014 [84]	3	2	3	8
Yuan et al., 2014 [63]	4	2	3	9
Kim et al., 2015 [78]	3	2	2	7
Wang et al., 2015 [64]	3	2	2	7
Haznadar et al., 2016 [65]	3	2	2	7
Klupczynska et al., 2016a [79]	3	2	2	7
Klupczynska et al., 2016b [80]	3	0	2	5
Ni et al., 2016 [81]	3	2	2	7
Ordóñez-Mena et al., 2016-ESTHER [42]	4	2	3	9
Ordóñez-Mena et al., 2016-TROMSØ [42]	4	2	2	8
Yuan et al., 2016 [66]	4	2	3	9
Yuan et al., 2017 [67]	4	2	3	9
Fanidi et al., 2018 [68]	3	2	2	7
Haznadar et al., 2018 [69]	3	2	2	7
Larose et al., 2018 [70]	2	2	2	6
Yue et al., 2018 [31]	3	1	2	6
Gao et al., 2019a–ESTHER [43]	3	2	3	8
Gao et al., 2019a–TROMSØ [43]	3	2	2	7
Gao et al., 2019b [71]	3	2	3	8
Kawamoto et al., 2019 [29]	3	2	2	7
Klupczynska et al., 2019 [35]	2	2	2	6
Ni et al., 2019 [36]	3	2	2	7
Pietzke et al., 2019 [25]	2	0	2	4
Seow et al., 2019 [72]	4	2	2	8
Zhang et al., 2019 [82]	2	0	2	4

*ESTHER* Epidemiologische Studie zu Chancen der Verhütung, Früherkennung und optimierten Therapie chronischer Erkrankungen in der älteren Bevölkerung cohort study, *TROMSØ* Tromsø study. ^a^ A maximum of four points may be awarded to the study based on: (for case-control studies) adequacy of case definition, representativeness of cases, selection of controls, and definition of controls; or, (for cohort studies) representativeness of exposed cohort, selection of non-exposed cohort, ascertainment of exposure, and demonstration that outcome of interest was not present at start of study. ^b^ A maximum of two points may be awarded to the study, based on the comparability between cases and controls (for case-control studies) or comparability between the exposed and non-exposed group (for cohort studies). One point was awarded if the study adjusted for age, and the other point was awarded if the following factors were controlled for or stratified during analysis: age, smoking status, gender. ^c^ A maximum of three points may be awarded to the study, based on: (for case-control studies) ascertainment of exposure, same method of ascertainment for cases and controls, and same non-response rate or, (for cohort studies) assessment of outcome, duration of follow-up for outcome, and adequacy of follow-up of cohorts. ^d^ For self-controlled case-control studies, a modified version of the NOS was used, with the exclusion of the question on the definition of controls, such that these studies could have a maximum of 8 points (instead of 9 points). The overall quality score of each of these studies was scaled to a maximum score of 9 points, in order to facilitate comparison across all studies.

**Table 3:** Odds ratio (OR) and 95% confidence interval (CI) of the association of lung cancer in relation to metabolites that achieved statistical significance. Refer to Figure S1 for the forest plot of each metabolite.

Metabolite.	OR	95% CI	No. of Studies	I^2^ (%)	Cochran’s Q Test’s *p*-Value	Forest Plot
Serum/Plasma						
Cotinine ^†^	14.19	2.92–69.00	3	96.8	<0.001	Figure S1a
Folate	0.82	0.72–0.94	2	47.7	0.167	Figure S1b
Urine						
Creatine Riboside ^†^	3.30	1.33–8.15	2	84.7	0.011	Figure S1c
NANA	2.01	1.49–2.72	2	0.0	0.458	Figure S1d
PheT	2.49	1.53–4.05	2	0.0	0.673	Figure S1e
Total 3-HC (3-HC + 3-HC-Gluc)	3.71	2.41–5.72	2	0.0	0.499	Figure S1f
Total Cotinine (Cotinine + Cotinine-Gluc)	3.53	2.62–4.77	3	0.0	0.406	Figure S1g
Total Nicotine (Nicotine + Nicotine-Gluc)	2.51	1.71–3.70	2	8.9	0.295	Figure S1h
Total NNAL (NNAL + NNAL-Glucs)	2.17	1.63–2.89	3	28.3	0.248	Figure S1i
Total Nicotine Equivalent (Total nicotine + Total cotinine + Total 3-HC)	3.75	2.45–5.73	2	16.3	0.274	Figure S1j

^†^ Random-effects models were used for this metabolite.

**Table 4:** Weighted mean difference (WMD) and 95% confidence interval (CI) of the of the plasma metabolite concentration between lung cancer patients and healthy controls, for metabolites that achieved statistical significance. Refer to Figure S2 for the forest plot of each metabolite.

Metabolite	WMD (μmol/L)	95% CI (μmol/L)	No. of Studies	I^2^ (%)	Cochran’s Q Test’s *p*-Value	Forest Plot
Plasma						
Methionine ^†^	−2.04	−4.01–−0.06	5	86.0	<0.001	Figure S2a
Tryptophan ^†^	−6.85	−11.07–−2.63	4	87.1	<0.001	Figure S2b
Proline ^†^	15.98	6.59–25.37	6	83.6	<0.001	Figure S2c

^†^ Random-effects models were used for this metabolite.

## Supplementary Materials

The following are available online at https://www.mdpi.com/2218-1989/10/9/362/s1, List S1: List of abbreviations used, List S2: Preferred Reporting items for Systematic Reviews and Meta-analysis (PRISMA) checklist, Table S1: Literature search strategy used for electronic databases, Table S2: Metabolites reported in categorical studies, Table S3: Metabolites solely reported in individual categorical studies, Table S4: Metabolites reported in concentration studies, Table S5: Metabolites solely reported in individual concentration studies, Figure S1: Effect size (ES; i.e., odds ratio (OR)) and 95% confidence interval (CI) of the association of lung cancer in relation to metabolites that achieved statistical significance, Figure S2: Weighted mean difference (WMD) and 95% confidence interval (CI) of the plasma metabolite concentration between lung cancer patients and healthy controls using a random-effects model, for metabolites that achieved statistical significance, Figure S3: Effect size (ES; i.e., odds ratio (OR)) and 95% confidence interval (CI) of the association of lung cancer in relation to levels of metabolite, for metabolites that did not achieve statistical significance, Figure S4: Effect size (ES; i.e., odds ratio (OR)) and 95% confidence interval (CI) of the association of lung cancer in relation to levels of metabolite, stratified by gender (for a–c) and smoking status (for d–h), Figure S5: Weighted mean difference (WMD) and 95% confidence interval (CI) of the metabolite concentration between lung cancer patients and healthy controls for metabolites that did not achieve statistical significance.
