# Supplementary material for: Association between Metabolites and the Risk of Lung Cancer: A Systematic Literature Review and Meta-Analysis of Observational Studies
Source: Metabolites. 2020 Sep 5;10(9):362. doi: 10.3390/metabo10090362 (PMC7570231; doi:10.3390/metabo10090362)
Supplement: Supplementary file 1 [file metabolites-10-00362-s001.pdf]

metabolites

MDPI

Supplementary Material

Association between Metabolites and the Risk of Lung Cancer: A Systematic Literature Review and Meta-Analysis of Observational Studies

Kian Boon Lee [1], Lina Ang [2], Wai-Ping Yau [1] and Wei Jie Seow [2,3,*]

[1] Department of Pharmacy, Faculty of Science, National University of Singapore, Singapore 117543, Singapore; lee_kian_boon@u.nus.edu (K.B.L.); phaywp@nus.edu.sg (W.-P.Y.)
[2] Saw Swee Hock School of Public Health, National University of Singapore and National University Health System, Singapore 117549, Singapore, Singapore; ephanli@nus.edu.sg
[3] Department of Medicine, Yong Loo Lin School of Medicine, National University of Singapore and National University Health System, Singapore 119228, Singapore
* Correspondence: ephswj@nus.edu.sg; Tel.: +65-6601-1243; Fax: +65-6779-1489

Contents

List S1. List of abbreviations used 3
List S2. Preferred Reporting items for Systematic Reviews and Meta-analysis (PRISMA) checklist .. 4
Table S1. Literature search strategy used for electronic databases. 6
Table S2. Metabolites reported in categorical studies. 8
Table S3. Metabolites solely reported in individual categorical studies. 9
Table S4. Metabolites reported in concentration studies. 10
Table S5. Metabolites solely reported in individual concentration studies. 14
Figure S1. Effect size (ES; i.e. odds ratio (OR)) and 95% confidence interval (CI) of the association of lung cancer in relation to metabolites that achieved statistical significance. 31
Figure S2. Weighted mean difference (WMD) and 95% confidence interval (CI) of the plasma metabolite concentration between lung cancer patients and healthy controls using a random-effects model, for metabolites that achieved statistical significance. 33
Figure S3. Effect size (ES; i.e. odds ratio (OR)) and 95% confidence interval (CI) of the association of lung cancer in relation to levels of metabolite, for metabolites that did not achieve statistical significance. 37
Figure S4. Effect size (ES; i.e. odds ratio (OR)) and 95% confidence interval (CI) of the association of lung cancer in relation to levels of metabolite, stratified by gender (for **a**-**c**) and smoking status (for **d**-**h**). 41
Figure S5. Weighted mean difference (WMD) and 95% confidence interval (CI) of the metabolite concentration between lung cancer patients and healthy controls for metabolites that did not achieve statistical significance. 60

List S1. List of abbreviations used

1,25(OH)$_2$ D	1,25-Dihydroxyvitamin D
25(OH) D	25-hydroxyvitamin D
3-HC	3-hydroxycotinine
3-OH-Phe	3-hydroxyphenanthrene
6KPGF1α	6-keto-prostaglandin F1-α
ADP	Adenosine diphosphate
AMP	Adenosine monophosphate
ATP	Adenosine triphosphate
cGMP	Cyclic guanosine monophosphate
GDP	Guanosine diphosphate
GMP	Guanosine monophosphate
GTP	Guanosine triphosphate
Gluc	Glucuronide
HBMA	4-hydroxybut-2-yl mercapturic acid
HEMA	2-hydroxyethyl mercapturic acid
HPMA	3-hydroxypropyl mercapturic acid
LPC	Lysophosphatidylcholine
MHBMA	Monohydroxybutyl mercapturic acid
NANA	N-acetylneuraminic acid
NNAL	4-(methylnitrosamino)-1-(3-pyridyl)-1-butanol
PC	Phosphatidylcholine
PG	Prostaglandin
PGE-MUM	7alpha-hydroxy-5,11-diketotetranor-prosta-1,16-dioic acid
PGEM-II	11-dcoxy-13,14-dihydro-15-keto-11,16-cyclo-prostaglandin E2
PheT	r-1,t-2,3,c-4-tetrahydroxy-1,2,3,4-tetrahydrophenanthrene
ROM	Reactive oxygen metabolite
SM	Sphingomyelin
SPMA	S-phenyl mercapturic acid
TNE	Total nicotine equivalent

List S2. Preferred Reporting items for Systematic Reviews and Meta-analysis (PRISMA) checklist

Section/topic	#	Checklist item	Reported in section
**TITLE**			
Title	1	Identify the report as a systematic review, meta-analysis, or both.	Title
**ABSTRACT**			
Structured summary	2	Provide a structured summary including, as applicable: background; objectives; data sources; study eligibility criteria, participants, and interventions; study appraisal and synthesis methods; results; limitations; conclusions and implications of key findings; systematic review registration number.	Abstract
**INTRODUCTION**			
Rationale	3	Describe the rationale for the review in the context of what is already known.	Section 1
Objectives	4	Provide an explicit statement of questions being addressed with reference to participants, interventions, comparisons, outcomes, and study design (PICOS).	Section 1
**METHODS**			
Protocol and registration	5	Indicate if a review protocol exists, if and where it can be accessed (e.g., Web address), and, if available, provide registration information including registration number.	-
Eligibility criteria	6	Specify study characteristics (e.g., PICOS, length of follow-up) and report characteristics (e.g., years considered, language, publication status) used as criteria for eligibility, giving rationale.	Section 2.2
Information sources	7	Describe all information sources (e.g., databases with dates of coverage, contact with study authors to identify additional studies) in the search and date last searched.	Section 2.1
Search	8	Present full electronic search strategy for at least one database, including any limits used, such that it could be repeated.	Table S1
Study selection	9	State the process for selecting studies (i.e., screening, eligibility, included in systematic review, and, if applicable, included in the meta-analysis).	Section 2.1 & 2.2
Data collection process	10	Describe method of data extraction from reports (e.g., piloted forms, independently, in duplicate) and any processes for obtaining and confirming data from investigators.	Section 2.3
Data items	11	List and define all variables for which data were sought (e.g., PICOS, funding sources) and any assumptions and simplifications made.	Section 2.3
Risk of bias in individual studies	12	Describe methods used for assessing risk of bias of individual studies (including specification of whether this was done at the study or outcome level), and how this information is to be used in any data synthesis.	Section 2.4
Summary measures	13	State the principal summary measures (e.g., risk ratio, difference in means).	Section 2.5

Section/topic	#	Checklist item	Reported in section
Synthesis of results	14	Describe the methods of handling data and combining results of studies, if done, including measures of consistency (e.g., I^2) for each meta-analysis.	Section 2.5
Risk of bias across studies	15	Specify any assessment of risk of bias that may affect the cumulative evidence (e.g., publication bias, selective reporting within studies).	- [a]
Additional analyses	16	Describe methods of additional analyses (e.g., sensitivity or subgroup analyses, meta-regression), if done, indicating which were pre-specified.	Section 2.5
**RESULTS**			
Study selection	17	Give numbers of studies screened, assessed for eligibility, and included in the review, with reasons for exclusions at each stage, ideally with a flow diagram.	Section 3.1 & Figure 1
Study characteristics	18	For each study, present characteristics for which data were extracted (e.g., study size, PICOS, follow-up period) and provide the citations.	Section 3.2 & Table 1
Risk of bias within studies	19	Present data on risk of bias of each study and, if available, any outcome level assessment (see item 12).	Section 3.3 & Table 2
Results of individual studies	20	For all outcomes considered (benefits or harms), present, for each study: (a) simple summary data for each intervention group (b) effect estimates and confidence intervals, ideally with a forest plot.	Sections 3.4 & 3.5; Figures S1, S2, S3, S4 & S5; Tables S3 & S5
Synthesis of results	21	Present results of each meta-analysis done, including confidence intervals and measures of consistency.	Sections 3.4 & 3.5; Tables 3 & 4; Figures S1, S2, S3, S4 & S5
Risk of bias across studies	22	Present results of any assessment of risk of bias across studies (see Item 15).	- [a]
Additional analysis	23	Give results of additional analyses, if done (e.g., sensitivity or subgroup analyses, meta-regression [see Item 16]).	Figure S4
**DISCUSSION**			
Summary of evidence	24	Summarize the main findings including the strength of evidence for each main outcome; consider their relevance to key groups (e.g., healthcare providers, users, and policy makers).	Section 4.1
Limitations	25	Discuss limitations at study and outcome level (e.g., risk of bias), and at review-level (e.g., incomplete retrieval of identified research, reporting bias).	Section 4.7
Conclusions	26	Provide a general interpretation of the results in the context of other evidence, and implications for future research.	Section 5
**FUNDING**			
Funding	27	Describe sources of funding for the systematic review and other support (e.g., supply of data); role of funders for the systematic review.	Funding

Checklist retrieved from PRISMA website [1].

[a] Risk of bias across studies was not assessed due to limited number of included studies (<10 for each meta-analysis) [2].

Table S1. Literature search strategy used for electronic databases.

For each database, the search performed is ((#1 AND #2) OR #3) AND #4.

PubMed

	Terms
1. Cancer	"Adenocarcinoma"[Mesh] OR "Carcinoma, Squamous Cell"[Mesh] OR "Neoplasms"[Mesh] OR<br>adenocarcinoma OR adenocarcinomas OR cancer OR cancers OR carcinogen OR carcinogenic OR carcinogens OR carcinoma OR carcinomas OR malignancy OR malignant OR metastasis OR neoplasia OR neoplasm OR neoplastic OR sarcoma OR tumor OR tumors OR tumour OR tumours OR<br>((adenoma OR adenomas) AND malignant) OR<br>(((squamous OR epidermoid OR planocellular) AND<br>(cancer OR cancers OR carcinoma OR carcinomas)) OR SCC)
2. Lung	"Lung"[Mesh] OR lung OR lungs OR pulmonary OR respiratory
3. Subtypes of lung cancer	"Bronchial Neoplasms"[Mesh] OR "Carcinoma, Non-Small-Cell Lung"[Mesh] OR "Lung Neoplasms"[Mesh] OR "Small Cell Lung Carcinoma"[Mesh] OR<br>((bronchial OR bronchioles OR bronchi) AND (cancer OR cancers OR carcinoma OR carcinomas OR neoplasm)) OR<br>NSCLC OR ((non-small-cell OR (non small cell) OR (non AND small AND cell) OR (non AND (small-cell OR smallcell)) OR ((non-small OR nonsmall) AND cell)) AND lung AND (cancer OR cancers OR carcinoma OR carcinomas OR neoplasm)) OR<br>SCLC OR ((small AND cell) OR smallcell OR small-cell) AND lung AND (cancer OR cancers OR carcinoma OR carcinomas OR neoplasm)
4. Metabolites / Metabolomics	"Metabolomics"[Mesh] OR "Metabolome"[Mesh] OR metabolomic OR metabolomics OR metabolome OR metabolite OR metabolites

Embase

	Terms
1. Cancer	'adenocarcinoma'/exp OR 'adenocarcinomas' OR 'cancer'/exp OR 'cancers'/exp OR 'carcinogen'/exp OR 'carcinogenic' OR 'carcinogens'/exp OR 'carcinoma'/exp OR 'carcinomas' OR 'malignancy'/exp OR 'malignant' OR 'metastasis'/exp OR 'neoplasia'/exp OR 'neoplasm'/exp OR 'neoplasms'/exp OR 'neoplastic' OR 'sarcoma'/exp OR 'tumor'/exp OR 'tumors'/exp OR 'tumour'/exp OR 'tumours' OR (('adenoma'/exp OR 'adenomas') AND ('malignant' OR 'malignancy'/exp)) OR (('squamous' OR 'epidermoid'/exp OR 'planocellular') AND ('cancer'/exp OR 'cancers'/exp OR 'carcinoma'/exp OR 'carcinomas')) OR 'scc'
2. Lung	'lung'/exp OR 'lungs'/exp OR 'pulmonary' OR 'respiratory'
3. Subtypes of lung cancer	'bronchial neoplasms'/exp OR (('bronchial' OR 'bronchioles'/exp OR 'bronchi'/exp) AND ('cancer'/exp OR 'cancers'/exp OR 'carcinoma'/exp OR 'carcinomas' OR 'neoplasm'/exp)) OR<br>'non small cell lung cancer'/exp OR 'nsclc' OR (('non small cell' OR 'non-small-cell' OR ('non' AND 'small' AND 'cell'/exp) OR ('non' AND ('small-cell' OR 'smallcell')) OR (('non-small' OR 'nonsmall') AND 'cell'/exp)) AND ('cancer'/exp OR 'cancers'/exp OR 'carcinoma'/exp OR 'carcinomas' OR 'neoplasm'/exp)) OR

	Terms
3. Subtypes of lung cancer (continued)	'small cell lung cancer'/exp OR (('small' AND'cell'/exp OR 'smallcell' OR 'small-cell') AND 'lung'/exp AND ('cancer'/exp OR 'cancers'/exp OR 'carcinoma'/exp OR 'carcinomas' OR 'neoplasm'/exp)) OR 'sclc'
4. Metabolites / Metabolomics	'metabolomic' OR 'metabolomics'/exp OR 'metabolome'/exp OR 'metabolite'/exp OR 'metabolites'/exp

Table S2. Metabolites reported in categorical studies.

**Biological Sample**	**Metabolites reported**	
Blood	3-Hydroxyantranilic acid [3]	Kynurenine [3]
	3-Hydroxykynurenine [3]	Neopterine [3]
	Anthralinic acid [3]	Tryptophan [3]
	Kynurenic acid [3]	Xanthurenic acid [3]
Plasma	25(OH)D [4,5]	
Serum	1,25$(OH)_2D_2$ [6]	Homocysteine [11]
	1,25$(OH)_2D_3$ [6]	Methionine [11]
	25(OH) D_2 [6]	PheT [13]
	25(OH) D_3 [6]	Pyridoxal 5'-phosphate [11]
	25(OH)D [7-10]	Riboflavin [11]
	Cobalamin [11]	ROM [14]
	Cotinine [12,13]	Total NNAL [a] [13]
	Folate [11]	
Plasma / Serum	Cotinine [15,16]	Methionine [17]
	Folate [17]	Pyridoxal 5'-phosphate [17]
Urine	1-hydroxypyrene [18]	Nitrate + Nitrite [28]
	2-Naphthol [18]	NNAL-Glucs [25]
	3-OH-Phe [19]	NNAL-N-Gluc [25]
	5-Methyl-2-Furoic Acid [20]	NNAL-O-Gluc [25]
	7-methylguanine [21]	PheT [19,29]
	8-hydroxydeoxyguanosine [18]	SPMA [19,26]
	Cortisol sulfate [22,23]	Thiobarbituric acid reactive substances [18]
	Cotinine [18,24]	Total 3-HC [b] [25,30]
	Creatine riboside [22,23]	Total antioxidant capacity [18]
	Free NNAL [25]	Total cotinine [c] [25,29,30]
	HBMA [19,26]	Total nicotine [d] [25,30]
	HEMA [19,26]	TNE [e] [25,30]
	HPMA [19,26]	Total nicotine metabolites [f] [31]
	Isothiocyanates [27]	Total NNAL [a] [25,29,30]
	MHBMA [26]	Total OH-Phe [g] [19]
	NANA [22,23]	

Refer to Supplementary material List 1 for the name of abbreviated metabolites.

[a] Total NNAL is defined as the total concentration of NNAL and its glucuronides
[b] Total 3-HC is defined as the total concentration of 3-HC and its glucuronides
[c] Total cotinine is defined as the total concentration of cotinine and its glucuronides
[d] Total nicotine is defined as the total concentration of nicotine and its glucuronides
[e] TNE is defined as the sum of the concentration of nicotine, cotinine, 3-HC and their respective glucuronides
[f] Total nicotine metabolite is defined as the total concentration of nicotine, cotinine and all their other pyridyl-containing metabolites
[g] Total OH-Phe is defined as the total concentration of 1-, 2-, 3- and 4-hydroxyphenanthrene

Table S3. Metabolites solely reported in individual categorical studies.

Reference	Metabolite	Measure of Association	Effect Size (95% CI)
Blood			
Chuang 2014 [3]	3-Hydroxyantranilic acid	OR	1.04 (0.73-1.49)
	3-Hydroxykynurenine	OR	1.12 (0.80-1.58)
	Anthralinic acid	OR	1.32 (0.91-1.91)
	Kynurenic acid	OR	0.79 (0.56-1.10)
	Kynurenine	OR	1.30 (0.92-1.84)
	Neopterine	OR	1.06 (0.76-1.49)
	Tryptophan	OR	0.88 (0.59-1.30)
Serum			
Church 2009 [13]	PheT	OR (per SD [87.2 pmol/L] increase)	1.23 (0.88-1.72)
Johansson 2010 [11]	Cobalamin	OR	1.20 (0.90-1.59)
	Folate	OR	0.68 (0.50-0.91)
	Homocysteine	OR	0.82 (0.60-1.12)
	Riboflavin	OR	0.85 (0.63-1.14)
Gao 2019a [14]	ROM	HR	ESTHER: 2.35 (1.01-5.50)<br>TROMSØ: 1.77 (1.09-2.87)
Serum / Plasma			
Larose 2018 [15]	Cotinine	OR (per 500nmol/L increase)	Active smokers: 1.39 (1.32-1.47)<br>Former smokers 1.17 (1.07-1.28)<br>Never smokers 1.64 (1.10-2.30)
Urine			
Ellard 1995 [31]	Total nicotine metabolites	OR	7.8 (4.3-14.0)
London 2000 [27]	Isothiocyanates	RR	0.65 (0.43-0.97)
Loft 2007 [21]	7-methylguanine	IRR	1.75 (1.04-2.95)
Yuan 2012 [26]	MHBMA	OR	1.08 (0.66-1.75)
Eom 2013 [18]	1-hydroxypyrene	OR	1.70 (0.74-4.03)
	2-Naphthol	OR	1.39 (0.58-3.44)
	8-hydroxydeoxyguanosine	OR	2.00 (0.87-4.78)
	Thiobarbituric acid reactive substances	OR	1.83 (0.78-4.41)
	Total antioxidant capacity	OR	0.60 (0.23-1.46)
Yuan 2014 [19]	3-OH-Phe	OR	3.10 (1.24-7.75)
	Total OH-Phe	OR	2.59 (1.01-6.65)
Yuan 2017 [25]	Free NNAL	OR	2.26 (1.25-4.09)
	NNAL-Glucs[a]	OR	1.58 (0.87–2.86)
	NNAL-N-Gluc	OR	1.92 (1.04-3.54)
	NNAL-O-Gluc	OR	1.51 (0.83-2.76)
Gao 2019b [28]	Nitrate & Nitrite	OR	1.37 (1.04-1.82)
Seow 2019 [20]	5-Methyl-2-Furoic Acid	OR	0.46 (0.30-0.70)

HR Hazard ratio, *IRR* Incidence rate ratio, *OR* Odds ratio, *RR* Risk ratio.

Refer to Supplementary material List 1 for the name of abbreviated metabolites.

[a] NNAL-Glucs is defined as the total concentration of NNAL-N-glucuronide and NNAL-O-glucuronide.

Table S4. Metabolites reported in concentration studies.

Biological Sample	Metabolites Reported	
Breath	Aniline [32]	Nitrate [33]
	Nitrite [33]	O-toluidine [32]
Hemolysed whole blood	Alanine [34]	Lysine [34]
	Asparagine [34]	Methionine [34]
	Aspartate [34]	Ornithine [34]
	Glutamate [34]	Phenylalanine [34]
	Glutamine [34]	Proline [34]
	Glycine [34]	Serine [34]
	Histidine [34]	Threonine [34]
	Hydroxyproline [34]	Tyrosine [34]
	Isoleucine [34]	Valine [34]
	Leucine [34]	
Plasma	PGEM-II [35]	LPC 18:3 (sn-2) [41]
	1-methyladenosine [36]	LPC 19:0 (sn-1) [41]
	1-methylhistidine [36]	LPC 19:0 (sn-2) [41]
	4-hydroxyproline [36]	LPC 19:1 (sn-1) [41]
	5′-methylthioadenosine [36]	LPC 19:1 (sn-2) [41]
	5-methylcytidine [36]	LPC 19:2 (sn-2) [41]
	$6KPGF_{1\alpha}$ [36]	LPC 20:0 (sn-1) [41]
	AMP [36]	LPC 20:0 (sn-2) [41]
	Alanine [34,37-40]	LPC 20:1 (sn-1) [41]
	Arginine [36-40]	LPC 20:1 (sn-2) [41]
	Asparagine [34,36-40]	LPC 20:2 (sn-1) [41]
	Aspartate [34,40]	LPC 20:2 (sn-2) [41]
	Choline [36]	LPC 20:3 (sn-1) [41]
	Citric acid [36]	LPC 20:3 (sn-2) [41]
	Citrulline [36,38,39]	LPC 20:4 (sn-1) [41]
	Creatinine [36]	LPC 20:4 (sn-2) [41]
	cGMP [36]	LPC 20:5 (sn-1) [41]
	Cystathionine [36]	LPC 20:5 (sn-2) [41]
	Formate [40]	LPC 22:1 (sn-2) [41]
	Gluconolactone [36]	LPC 22:2 (sn-2) [41]
	Glutamate [34,36,40]	LPC 22:4 (sn-1) [41]
	Glutamine [36-40]	LPC 22:4 (sn-2) [41]
	Glycine [34,36-40]	LPC 22:5 (sn-1) [41]
	Histidine [34,36-39]	LPC 22:5 (sn-2) [41]
	Hydroxyproline [34]	LPC 22:6 (sn-1) [41]
	Isoleucine [34,37-40]	LPC 22:6 (sn-2) [41]
	Leucine [34,37-40]	Malic acid [36]
	Lysine [34,37-40]	Melatonin [42]
	LPC 14:0 (sn-1) [41]	Methionine [34,37-40]
	LPC 14:0 (sn-2) [41]	N,N-dimethylglycine [36]
	LPC 15:0 (sn-1) [41]	NG,NG-dimethylarginine [36]
	LPC 15:0 (sn-2) [41]	Niacinamide [36]
	LPC 16:0 (sn-1) [41]	Nicotinylglycine [36]
	LPC 16:0 (sn-2) [41]	Ornithine [34,36-39]
	LPC 17:0 (sn-1) [41]	Phenylalanine [34,36-40]
	LPC 17:0 (sn-2) [41]	Proline [34,36-40]

Biological Sample	Metabolites Reported	
Plasma (continued)	LPC 17:1 (sn-1) [41]	Pyroglutamic acid [36]
	LPC 17:1 (sn-2) [41]	Serine [34,36-40]
	LPC 18:0 (sn-1) [41]	Succinic acid [36]
	LPC 18:0 (sn-2) [41]	Testosterone [36]
	LPC 18:1 (sn-1) [41]	Threonine [34,36-40]
	LPC 18:1 (sn-2) [41]	Tryptophan [37-40,42]
	LPC 18:2 (sn-1) [41]	Tyrosine [34,37-40]
	LPC 18:2 (sn-2) [41]	Valine [34,37-40]
	LPC 18:3 (sn-1) [41]	Xanthine [36]
Serum	1-methylhistidine [43]	Diacyl-PC C38:0 [45]
	2-hydroxybutyric acid [44]	Diacyl-PC C38:1 [45]
	25(OH)D [9]	Diacyl-PC C38:3 [45]
	3-methylhistidine [43]	Diacyl-PC C38:4 [45]
	NNAL [13]	Diacyl-PC C38:5 [45]
	Acyl-alkyl-PC C30:0 [45]	Diacyl-PC C38:6 [45]
	Acyl-alkyl-PC C30:1 [45]	Diacyl PC C40:2 [45]
	Acyl-alkyl-PC C32:1 [45]	Diacyl-PC C40:3 [45]
	Acyl-alkyl-PC C32:2 [45]	Diacyl-PC C40:4 [45]
	Acyl-alkyl-PC C34:0 [45]	Diacyl-PC C40:5 [45]
	Acyl-alkyl-PC C34:1 [45]	Diacyl-PC C40:6 [45]
	Acyl-alkyl-PC C34:2 [45]	Diacyl-PC C42:0 [45]
	Acyl-alkyl-PC C34:3 [45]	Diacyl-PC C42:1 [45]
	Acyl-alkyl-PC C36:0 [45]	Diacyl-PC C42:2 [45]
	Acyl-alkyl-PC C36:1 [45]	Diacyl PC C42:4 [45]
	Acyl-alkyl-PC C36:2 [45]	Diacyl-PC C42:5 [45]
	Acyl-alkyl-PC C36:3 [45]	Ethanolamine [43]
	Acyl-alkyl-PC C36:4 [45]	Fumaric acid [44]
	Acyl-alkyl-PC C36:5 [45]	Glutamate [46]
	Acyl-alkyl-PC C38:0 [45]	Glutamic acid [43]
	Acyl-alkyl-PC C38:1 [45]	Glutamine [43]
	Acyl-alkyl-PC C38:2 [45]	Glutaric acid [44]
	Acyl-alkyl-PC C38:3 [45]	Glycine [43,46,47]
	Acyl-alkyl-PC C38:4 [45]	Histidine [43,46,47]
	Acyl-alkyl-PC C38:5 [45]	Hydroxyproline [43]
	Acyl-alkyl-PC C38:6 [45]	Isoleucine [43]
	Acyl-alkyl-PC C40:1 [45]	Lactic Acid [44]
	Acyl-alkyl-PC C40:2 [45]	Leucine [43,46,47]
	Acyl-alkyl-PC C40:3 [45]	Lysine [43]
	Acyl-alkyl-PC C40:4 [45]	LPC acyl C16:0 [45]
	Acyl-alkyl-PC C40:5 [45]	LPC acyl C16:1 [45]
	Acyl-alkyl-PC C40:6 [45]	LPC acyl C17:0 [45]
	Acyl-alkyl PC C42:1 [45]	LPC acyl C18:0 [45]
	Acyl-alkyl-PC C42:2 [45]	LPC acyl C18:1 [45]
	Acyl-alkyl-PC C42:3 [45]	LPC acyl C18:2 [45]
	Acyl-alkyl-PC C42:4 [45]	LPC acyl C20:3 [45]
	Acyl-alkyl PC C44:3 [45]	LPC acyl C20:4 [45]
	Acyl-alkyl-PC C44:4 [45]	LPC acyl C26:0 [45]
	Acyl-alkyl-PC C44:5 [45]	LPC acyl C26:1 [45]
	Acyl-alkyl-PC C44:6 [45]	LPC acyl C28:0 [45]
	Alanine [43,46,47]	LPC acyl C28:1 [45]

Biological Sample	Metabolites Reported	
Serum (continued)	Arginine [43,46,47]	Malondialdehyde [48]
	Ascorbic acid [48]	Methionine [43,46,47]
	Asparagine [43]	Nitrate [48]
	Aspartate [46]	Nitrite [48]
	Aspartic acid [43]	Ornithine [43,46,47]
	C0-carnitine [45]	Phenylalanine [43,46,47]
	C2-carnitine [45-47]	Proline [43]
	C3-carnitine [46,47]	Pyroglutamic acid [44]
	C4-carnitine [46,47]	ROM [49]
	C5-carnitine [46,47]	PheT [13]
	C6-carnitine [46,47]	Reduced glutathione [48]
	C8-carnitine [46,47]	Retinol [48]
	C10:1-carnitine [45]	Sarcosine [43]
	C12:1-carnitine [45]	Serine [43]
	C14-carnitine [46,47]	SM (OH) C14:1 [45]
	C14:1-carnitine [45]	SM (OH) C16:1 [45]
	C14:2-carnitine [45]	SM (OH) C22:1 [45]
	C16-carnitine [46,47]	SM (OH) C22:2 [45]
	C18:1-carnitine [45]	SM (OH) C24:1 [45]
	C18:2-carnitine [45]	SM C16:0 [45]
	Citrulline [43,46,47]	SM C16:1 [45]
	Cotinine [13]	SM C18:0 [45]
	Diacyl-PC C28:1 [45]	SM C18:1 [45]
	Diacyl-PC C30:0 [45]	SM C20:2 [45]
	Diacyl-PC C30:2 [45]	SM C22:3 [45]
	Diacyl-PC C32:0 [45]	SM C24:0 [45]
	Diacyl-PC C32:1 [45]	SM C24:1 [45]
	Diacyl-PC C32:2 [45]	SM C26:0 [45]
	Diacyl-PC C32:3 [45]	SM C26:1 [45]
	Diacyl-PC C34:1 [45]	Succinic acid [44]
	Diacyl-PC C34:2 [45]	Taurine [43]
	Diacyl-PC C34:3 [45]	Threonine [43]
	Diacyl PC C34:4 [45]	Tryptophan [43]
	Diacyl-PC C36:0 [45]	Tyrosine [43,46,47]
	Diacyl-PC C36:1 [45]	Valine [43,46,47]
	Diacyl-PC C36:2 [45]	α-aminoadipic acid [43]
	Diacyl-PC C36:3 [45]	α-amino-N-butyric acid [43]
	Diacyl-PC C36:4 [45]	β-alanine [43]
	Diacyl-PC C36:5 [45]	β-aminoisobutyric acid [43]
	Diacyl-PC C36:6 [45]	β-carotene [48]
Tumor Tissue	3-phosphoglycerate [50]	Histidine [50]
	ADP [50]	Isocitrate [50]
	AMP [50]	Isoleucine [50]
	ATP [50]	Lactate [50]
	Alanine [50]	Leucine [50]
	Arginine [50]	Lysine [50]
	Asparagine [50]	Malate [50]
	Aspartic acid [50]	Methionine [50]
	cis-aconitate [50]	Phenylalanine [50]
	Citrate [50]	Phosphoenolpyruvate [50]

Biological Sample	Metabolites Reported	
Tumor Tissue (continued)	Dihydroxyacetone phosphate [50]	Proline [50]
	Fructose 1,6-bisphosphate [50]	PGE [51]
	Fructose 6-phosphate [50]	PGE_2 [51]
	Fumarate [50]	Pyruvate [50]
	Glucose-6-phosphate [50]	ROM [52]
	Glutamic acid [50]	Serine [51]
	Glutamine [50]	Succinate [51]
	Glycine [50]	Threonine [51]
	GDP [50]	Tryptophan [51]
	GMP [50]	Tyrosine [51]
	GTP [50]	Valine [51]
Urine	6-sulfatoxymelatonin [42]	PGE-MUM [55]
	NNAL [53,54]	

Refer to Supplementary material List 1 for the name of abbreviated metabolites.

Table S5. Metabolites solely reported in individual concentration studies.

Ref.	Metabolite	Cancer cases		Controls		Unit	p-value
		No.	Metabolite conc.	No.	Metabolite conc.		
Exhaled breath							
Preti 1988 [32]	Aniline	10	0.65 ± 0.43 [a] (ND: 5) [b]	8	- (ND: 8) [b]	ng/L	NR
	O-toluidine	10	0.38 ± 0.26 [a] (ND: 1) [b]	8	0.15 ± 0.08 (ND: 2) [b]	ng/L	NR
Masri 2005 [33]	Nitric oxide	11	1.84 ± 3.16 [a]	35	7.40 ± 0.54 [a]	ppb	0.001
	Nitrate	9	10.01 ± 2.10 [a]	34	16.75 ± 1.20 [a]	µmol/L	0.014
	Nitrite	9	5.83 ± 2.10 [a]	34	1.93 ± 0.27 [a]	µmol/L	0.047
Hemolyzed Whole Blood							
Proenza 2003 [34]	Alanine	14	137.0 ± 44.9 [a]	14	108.0 ± 52.4 [a]	µmol/L	NS
	Asparagine	14	52.9 ± 22.4 [a]	14	48.3 ± 19.8 [a]	µmol/L	NS
	Aspartate	14	12.0 ± 59.9 [a]	14	78.5 ± 52.4 [a]	µmol/L	NS
	Glutamate	14	166.0 ± 44.9 [a]	14	168.0 ± 37 [a]	µmol/L	NS
	Glutamine	14	208.0 ± 37.4 [a]	14	159.0 ± 48.6 [a]	µmol/L	NS
	Glycine	14	136.0 ± 24.3 [a]	14	118.0 ± 31.4 [a]	µmol/L	NS
	Histidine	14	18.0 ± 10.5 [a]	14	14.6 ± 5.2 [a]	µmol/L	NS
	Hydroxyproline	14	6.5 ± 3.4 [a]	14	4.5 ± 3.7 [a]	µmol/L	NS
	Isoleucine	14	19.1 ± 8.2 [a]	14	12.9 ± 5.2 [a]	µmol/L	NS
	Leucine	14	45.2 ± 14.2 [a]	14	31.7 ± 10.9 [a]	µmol/L	NS
	Lysine	14	7.9 ± 10.1 [a]	14	3.3 ± 18.7 [a]	µmol/L	NS
	Methionine	14	5.9 ± 1.9 [a]	14	4.7 ± 3.7 [a]	µmol/L	NS
	Ornithine	14	33.0 ± 20.6 [a]	14	25.8 ± 12.3 [a]	µmol/L	NS
	Phenylalanine	14	16.9 ± 5.2 [a]	14	14.7 ± 6.7 [a]	µmol/L	NS
	Proline	14	58.5 ± 20.6 [a]	14	36.8 ± 22.8 [a]	µmol/L	<0.05
	Serine	14	55.5 ± 22.4 [a]	14	38.7 ± 17.2 [a]	µmol/L	NS
	Threonine	14	42.4 ± 17.2 [a]	14	26.5 ± 10.1 [a]	µmol/L	NS
	Tyrosine	14	28.3 ± 7.5 [a]	14	20.0 ± 5.6 [a]	µmol/L	NS

Ref.	Metabolite	Cancer cases		Controls		Unit	p-value
		No.	Metabolite conc.	No.	Metabolite conc.		
Proenza 2003 [34] (continued)	Valine	14	75.8 ± 27.3 [a]	14	50.9 ± 19.1 [a]	µmol/L	<0.05
			Plasma				
Hendrick 1988 [35]	6KPGF$_{1}\alpha$	29	13.6 (9.3-18.7) [c]	18	99.4 (95.9-113.8) [c]	ng/L	<0.002
	PGEM-II	29	21.3 (16.3-27.0) [c]	18	132.3 (113-152.8) [c]	ng/L	<0.01
Proenza 2003 [34]	Hydroxyproline	14	11.0 ± 5.2 [a]	14	11.8 ± 4.9 [a]	µmol/L	NS
Yue 2018 [36]	1-Methyladenosine	20	7396.10 ± 1303.52[a]	20	5521.85 ± 792.75[a]	µg/L	4.41×10^{-10}
	1-Methylhistidine	20	3102.10 ± 695.01[a]	20	1912.36 ± 722.3[a]	µg/L	4.35×10^{-4}
	4-Hydroxyproline	20	303.28 ± 107.42[a]	20	131.24 ± 59.38[a]	µg/L	5.05×10^{-4}
	5′-Methylthioadenosine	20	802.62 ± 172.27[a]	20	311.79 ± 67.04[a]	µg/L	5.46×10^{-3}
	5-Methylcytidine	20	73.17 ± 33.40[a]	20	103.46 ± 35.56[a]	µg/L	5.01×10^{-4}
	AMP	20	3240.20 ± 1091.67[a]	20	1211.94 ± 319.08[a]	µg/L	3.91×10^{-5}
	cGMP	20	16847.50 ± 2023.57[a]	20	14360.00 ± 2034.44[a]	µg/L	1.17×10^{-4}
	Choline	20	5.34 ± 1.52[a]	20	2.28 ± 1.43[a]	µg/L	2.34×10^{-14}
	Citric Acid	20	135.09 ± 38.09[a]	20	29.47 ± 6.40[a]	µg/L	1.87×10^{-7}
	Creatinine	20	1.93 ± 0.92[a]	20	3.32 ± 1.89[a]	µg/L	2.81×10^{-6}
	Cystathionine	20	1116.98 ± 638.10[a]	20	634.97 ± 611.20[a]	µg/L	1.04×10^{-7}
	Gluconolactone	20	224.84 ± 120.50[a]	20	112.48 ± 38.85[a]	µg/L	5.91×10^{-8}
	Malic Acid	20	398.03 ± 103.15[a]	20	623.55 ± 63.80[a]	µg/L	9.29×10^{-6}
	N,N-Dimethylglycine	20	4.54 ± 2.62[a]	20	1.30 ± 1.73[a]	µg/L	6.51×10^{-4}
	NG,NG-Dimethylarginine	20	438.06 ± 85.80[a]	20	342.24 ± 77.10[a]	µg/L	9.60×10^{-15}
	Niacinamide	20	26303.50 ± 4458.58[a]	20	22834.50 ± 2545.10[a]	µg/L	0.008
	Nicotinylglycine	20	2.19 ± 0.59[a]	20	3.18 ± 0.96[a]	µg/L	3.50×10^{-4}
	Pyroglutamic Acid	20	2.91 ± 0.84[a]	20	1.71 ± 0.92[a]	µg/L	1.23×10^{-9}
	Succinic Acid	20	208.88 ± 46.71[a]	20	1181.10 ± 867.10[a]	µg/L	3.43×10^{-6}
	Testosterone	20	9562.95 ± 1219.14[a]	20	8107.15 ± 1450.78[a]	µg/L	4.33×10^{-5}

Ref.	Metabolite	Cancer cases		Controls		Unit	p-value
		No.	Metabolite conc.	No.	Metabolite conc.		
Yue 2018 [36] (continued)	Xanthine	20	10.84 ± 4.51[a]	20	16.48 ± 4.84[a]	µg/L	3.10×10^{-4}
Pietzke 2019 [40]	Formate	56	41.8 (33.0-49.8)[d]	50	69.9 (61.7-78.2)[d]	µmol/L	$<10^{-6}$
Zhang 2019 [41]	LPC 14:0 (sn-1)	28	0.07680 ± 0.03800[a]	38	0.0986 ± 0.0369[a]	mg/L	<0.05
	LPC 14:0 (sn-2)	28	0.4160 ± 0.2128[a]	38	0.5436 ± 0.2125[a]	mg/L	<0.05
	LPC 15:0 (sn-1)	28	0.06870 ± 0.02290[a]	38	0.0822 ± 0.0355[a]	mg/L	NS
	LPC 15:0 (sn-2)	28	0.3168 ± 0.0990[a]	38	0.3719 ± 0.1394[a]	mg/L	NS
	LPC 16:0 (sn-1)	28	11.94 ± 3.68[a]	38	13.66 ± 3.90[a]	mg/L	NS
	LPC 16:0 (sn-2)	28	50.24 ± 12.85[a]	38	57.24 ± 10.81[a]	mg/L	<0.05
	LPC 17:0 (sn-1)	28	0.4321 ± 0.2517[a]	38	0.5819 ± 0.3672[a]	mg/L	NS
	LPC 17:0 (sn-2)	28	0.818 ± 0.279[a]	38	0.837 ± 0.328[a]	mg/L	<0.05
	LPC 17:1 (sn-1)	28	0.02520 ± 0.01040[a]	38	0.03090 ± 0.01480[a]	mg/L	NS
	LPC 17:1 (sn-2)	28	0.1240 ± 0.0437[a]	38	0.1465 ± 0.0652[a]	mg/L	NS
	LPC 18:0 (sn-1)	28	4.396 ± 1.461[a]	38	5.118 ± 1.596[a]	mg/L	NS
	LPC 18:0 (sn-2)	28	25.74 ± 7.80[a]	38	29.14 ± 8.79[a]	mg/L	NS
	LPC 18:1 (sn-1)	28	2.114 ± 0.731[a]	38	2.810 ± 0.875[a]	mg/L	<0.01
	LPC 18:1 (sn-2)	28	11.32 ± 3.95[a]	38	12.70 ± 4.64[a]	mg/L	NS
	LPC 18:2 (sn-1)	28	3.588 ± 1.400[a]	38	5.040 ± 1.719[a]	mg/L	<0.01
	LPC 18:2 (sn-2)	28	14.72 ± 4.84[a]	38	22.19 ± 7.30[a]	mg/L	<0.05
	LPC 18:3 (sn-1)	28	0.07690 ± 0.03780[a]	38	0.0853 ± 0.0406[a]	mg/L	NS
	LPC 18:3 (sn-2)	28	0.3334 ± 0.1734[a]	38	0.3605 ± 0.1670[a]	mg/L	NS
	LPC 19:0 (sn-1)	28	0.04420 ± 0.01880[a]	38	0.05820 ± 0.04870[a]	mg/L	NS
	LPC 19:0 (sn-2)	28	0.1037 ± 0.0333[a]	38	0.1095 ± 0.0380[a]	mg/L	NS
	LPC 19:1 (sn-1)	28	0.00980 ± 0.00380[a]	38	0.01210 ± 0.00490[a]	mg/L	<0.05
	LPC 19:1 (sn-2)	28	0.04230 ± 0.01520[a]	38	0.05490 ± 0.02020[a]	mg/L	<0.01
	LPC 19:2 (sn-2)	28	0.006800 ± 0.002600[a]	38	0.01080 ± 0.00490[a]	mg/L	<0.01
	LPC 20:0 (sn-1)	28	0.02340 ± 0.00770[a]	38	0.03180 ± 0.01240[a]	mg/L	<0.01
	LPC 20:0 (sn-2)	28	0.1364 ± 0.0418[a]	38	0.1602 ± 0.0609[a]	mg/L	NS
	LPC 20:1 (sn-1)	28	0.04990 ± 0.02020[a]	38	0.0616 ± 0.0329[a]	mg/L	NS

Ref.	Metabolite	Cancer cases		Controls		Unit	p-value
		No.	Metabolite conc.	No.	Metabolite conc.		
Zhang 2019 [41] (continued)	LPC 20:1 (sn-2)	28	0.2639 ± 0.0989[a]	38	0.2742 ± 0.1421[a]	mg/L	NS
	LPC 20:2 (sn-1)	28	0.07250 ± 0.03040[a]	38	0.0846 ± 0.0324[a]	mg/L	NS
	LPC 20:2 (sn-2)	28	0.3086 ± 0.1387[a]	38	0.3191 ± 0.1237[a]	mg/L	NS
	LPC 20:3 (sn-1)	28	0.3652 ± 0.1245[a]	38	0.4395 ± 0.1333[a]	mg/L	<0.05
	LPC 20:3 (sn-2)	28	0.905 ± 0.358[a]	38	1.195 ± 0.382[a]	mg/L	<0.01
	LPC 20:4 (sn-1)	28	0.952 ± 0.360[a]	38	1.084 ± 0.400[a]	mg/L	NS
	LPC 20:4 (sn-2)	28	3.363 ± 1.260[a]	38	4.076 ± 1.531[a]	mg/L	<0.05
	LPC 20:5 (sn-1)	28	0.05140 ± 0.02740[a]	38	0.06060 ± 0.04670[a]	mg/L	NS
	LPC 20:5 (sn-2)	28	0.1729 ± 0.0919[a]	38	0.2082 ± 0.1655[a]	mg/L	NS
	LPC 22:1 (sn-2)	28	0.02190 ± 0.01050[a]	38	0.02230 ± 0.01110[a]	mg/L	NS
	LPC 22:2 (sn-2)	28	0.00990 ± 0.00390[a]	38	0.00930 ± 0.00350[a]	mg/L	NS
	LPC 22:4 (sn-1)	28	0.02360 ± 0.01010[a]	38	0.03250 ± 0.01220[a]	mg/L	<0.01
	LPC 22:4 (sn-2)	28	0.07160 ± 0.02840[a]	38	0.0881 ± 0.0331[a]	mg/L	<0.05
	LPC 22:5 (sn-1)	28	0.07990 ± 0.02510[a]	38	0.0933 ± 0.0434[a]	mg/L	NS
	LPC 22:5 (sn-2)	28	0.2092 ± 0.0828[a]	38	0.2638 ± 0.0962[a]	mg/L	<0.05
	LPC 22:6 (sn-1)	28	0.2384 ± 0.0767[a]	38	0.2827 ± 0.1347[a]	mg/L	NS
	LPC 22:6 (sn-2)	28	0.7230 ± 0.2652[a]	38	0.7780 ± 0.3760[a]	mg/L	NS
		Serum					
Gencer 2006 [49]	ROM	14[e]	405±32[a]				<0.01
		12[f]	483±40[a]	26	240 ± 74[a]	U/L	<0.001
		12[g]	437±35[a]				<0.01
Esme 2008 [48]	Ascorbic acid	49	12.3 ± 4.2[a]	22	14.5 ± 3.9[a]	mg/L	<0.05
	Malondialdehyde	49	1.87 ± 0.66[a]	22	1.30 ± 0.35[a]	μmol/L	<0.01
	Nitrate	49	2.71 ± 0.91[a]	22	0.88 ± 0.14[a]	mg/L	<0.001
	Nitrite	49	7.71 ± 2.55[a]	22	6.06 ± 1.14[a]	mg/L	<0.01
	Reduced glutathione	49	242 ± 81[a]	22	368 ± 36[a]	mg/L	<0.001
	Retinol	49	535 ± 85[a]	22	575 ± 66[a]	mg/L	<0.05
	β-carotene	49	211 ± 49[a]	22	243 ± 30[a]	mg/L	<0.01
Church 2009 [13]	Cotinine	100	227 ± 93[a]	100	217 ± 111[a]	μg/L	NS
	PheT	100	92.5 ± 107.6[a]	100	76.3 ± 66.8[a]	pmol/L	NS

Ref.	Metabolite	Cancer cases		Controls		Unit	p-value
		No.	Metabolite conc.	No.	Metabolite conc.		
Church 2009 [13] (continued)	Total NNAL	100	92.4 ± 40.7[a]	100	77.4 ± 39.3[a]	pmol/L	0.0084
Skaaby 2014 [9]	25(OH)D	126	24.9 ± 126[a]	10485	51.8 ± 26.5[a]	nmol/L	NR
Klupczynska 2016a [44]	2-Hydroxybutyric Acid	90	72.43 ± 33.38[a]	62	58.99 ± 33.39[a]	µmol/L	0.00283
	Fumaric Acid	90	1.29 ± 1.15[a]	62	1.5 ± 1.22[a]	µmol/L	0.0003
	Glutaric Acid	90	0.52 ± 0.23[a]	62	0.52 ± 0.2[a]	µmol/L	NS
	Lactic Acid	90	1.89 ± 0.71[a]	62	2.2 ± 0.8[a]	mmol/L	0.00979
	Pyroglutamic Acid	90	26.99 ± 11.56[a]	62	35.28 ± 10.22[a]	µmol/L	2.64×10^{-8}
	Succinic Acid	90	8.52 ± 2.23[a]	62	8.9 ± 2.04[a]	µmol/L	NS
Klupczynska 2016b [43]	1-Methylhistidine	90	7 ± 5.85[a]	63	8.19 ± 7.61[a]	µmol/L	NS
	3-Methylhistidine	90	3.76 ± 2.08[a]	63	3.44 ± 1.23[a]	µmol/L	NS
	Asparagine	90	49.26 ± 11.31[a]	63	46.1 ± 8.85[a]	µmol/L	NS
	Aspartic Acid	90	19.23 ± 9.5[a]	63	12.64 ± 6.01[a]	µmol/L	<0.0001
	Ethanolamine	90	9.69 ± 2.29[a]	63	9.85 ± 2.29[a]	µmol/L	NS
	Glutamic Acid	90	80.81 ± 36.92[a]	63	72.48 ± 40.75[a]	µmol/L	NS
	Glutamine	90	502.11 ± 103.48[a]	63	489.72 ± 98.95[a]	µmol/L	NS
	Hydroxyproline	90	9.79 ± 4.23[a]	63	11.91 ± 7.61[a]	µmol/L	NS
	Isoleucine	90	65.77 ± 19.8[a]	63	66.11 ± 15.1[a]	µmol/L	NS
	Lysine	90	196.48 ± 55.51[a]	63	190.67 ± 46.22[a]	µmol/L	NS
	Proline	90	161.39 ± 50.88[a]	63	183.31 ± 65.84[a]	µmol/L	0.0329
	Sarcosine	90	1.26 ± 0.64[a]	63	1.25 ± 0.52[a]	µmol/L	NS
	Serine	90	142.71 ± 39.77[a]	63	126.18 ± 32.19[a]	µmol/L	0.0071
	Taurine	90	108.89 ± 43.22[a]	63	113.71 ± 47.39[a]	µmol/L	NS
	Threonine	90	106.65 ± 30.11[a]	63	106.1 ± 28.4[a]	µmol/L	NS
	Tryptophan	90	42.43 ± 13[a]	63	45.51 ± 9.94[a]	µmol/L	NS
	α-Aminoadipic Acid	90	0.86 ± 0.48[a]	63	0.88 ± 0.33[a]	µmol/L	NS
	α-Amino-N-Butyric Acid	90	23.25 ± 8.94[a]	63	23.43 ± 7.69[a]	µmol/L	NS
	β-Alanine	90	22.61 ± 10.27[a]	63	15.89 ± 9.1[a]	µmol/L	0.0001
	β-Aminoisobutyric Acid	90	2.11 ± 0.95[a]	63	2.19 ± 1.19[a]	µmol/L	NS
Ni 2016 [46]	Glutamate	40	76.50 ± 21.56[a]	100	87.55 ± 27.25[a]	µmol/L	0.0234

Ref.	Metabolite	Cancer cases		Controls		Unit	p-value
		No.	Metabolite conc.	No.	Metabolite conc.		
Klupczynska 2019 [45]	C0-carnitine	20	43.9 (37.6-52.1)[d]	20	41.0 (33.3-45.9)[d]	µmol/L	NR
	C10:1-carnitine	20	0.135 (0.104-0.183)[d]	20	0.121 (0.073-0.153)[d]	µmol/L	NR
	C12:1-carnitine	20	0.164 (0.150-0.247)[d]	20	0.164 (0.145-0.201)[d]	µmol/L	NR
	C14:1-carnitine	20	0.118 (0.099-0.150)[d]	20	0.104 (0.083-0.170)[d]	µmol/L	NR
	C14:2-carnitine	20	0.041 (0.035-0.054)[d]	20	0.036 (0.027-0.05)[d]	µmol/L	NR
	C18:1-carnitine	20	0.136 (0.115-0.193)[d]	20	0.162 (0.133-0.199)[d]	µmol/L	NR
	C18:2-carnitine	20	0.041 (0.034-0.054)[d]	20	0.052 (0.041-0.063)[d]	µmol/L	NR
	LPC acyl C16:0	20	79.8 (68.2-97.6)[d]	20	94.0 (80.9-107.5)[d]	µmol/L	NR
	LPC acyl C16:1	20	2.61 (1.93-3.25)[d]	20	3.14 (2.59-3.54)[d]	µmol/L	NR
	LPC acyl C17:0	20	5.75 (4.91-7.13)[d]	20	6.96 (5.92-8.07)[d]	µmol/L	NR
	LPC acyl C18:0	20	24.2 (19.8-28.4)[d]	20	28.5 (23.6-34.8)[d]	µmol/L	NR
	LPC acyl C18:1	20	15.9 (12.0-20.0)[d]	20	20.3 (14.9-23.8)[d]	µmol/L	NR
	LPC acyl C18:2	20	15.5 (11.5-18.2)[d]	20	21.3 (14.8-26.9)[d]	µmol/L	NR
	LPC acyl C20:3	20	1.66 (1.55-2.17)[d]	20	2.04 (1.80-2.64)[d]	µmol/L	NR
	LPC acyl C20:4	20	5.93 (4.87-7.27)[d]	20	7.57 (6.64-8.50)[d]	µmol/L	NR
	LPC acyl C26:0	20	0.506 (0.422-0.618)[d]	20	0.321 (0.206-0.438)[d]	µmol/L	NR
	LPC acyl C26:1	20	0.265 (0.207-0.322)[d]	20	0.183 (0.135-0.243)[d]	µmol/L	NR
	LPC acyl C28:0	20	0.472 (0.415-0.601)[d]	20	0.333 (0.233-0.489)[d]	µmol/L	NR
	LPC acyl C28:1	20	0.651 (0.561-0.750)[d]	20	0.534 (0.474-0.660)[d]	µmol/L	NR
	Diacyl-PC C28:1	20	2.03 (1.52-2.38)[d]	20	2.38 (1.86-2.68)[d]	µmol/L	NR

Ref.	Metabolite	Cancer cases		Controls		Unit	p-value
		No.	Metabolite conc.	No.	Metabolite conc.		
Klupczynska 2019 [45] (continued)	Diacyl-PC C30:0	20	7.68 (6.36-8.27)[d]	20	8.38 (7.36-8.90)[d]	µmol/L	NR
	Diacyl-PC C30:2	20	0.876 (0.751-1.005)[d]	20	0.964 (0.811-1.29)[d]	µmol/L	NR
	Diacyl-PC C32:0	20	12.1 (10.9-13.1)[d]	20	11.6 (10.8-12.6)[d]	µmol/L	NR
	Diacyl-PC C32:1	20	12.0 (10.0-15.7)[d]	20	12.5 (9.88-16.6)[d]	µmol/L	NR
	Diacyl-PC C32:2	20	1.38 (0.868-1.66)[d]	20	2.01 (1.83-2.82)[d]	µmol/L	NR
	Diacyl-PC C32:3	20	0.641 (0.487-0.706)[d]	20	0.623 (0.569-0.764)[d]	µmol/L	NR
	Diacyl-PC C34:1	20	181 (164-210)[d]	20	174 (156-198)[d]	µmol/L	NR
	Diacyl-PC C34:2	20	247 (223-266)[d]	20	246 (233-273)[d]	µmol/L	NR
	Diacyl-PC C34:3	20	9.75 (8.71-12.3)[d]	20	11.4 (9.51-12.9)[d]	µmol/L	NR
	Diacyl-PC C34:4	20	0.903 (0.676-1.03)[d]	20	1.45 (1.15-1.71)[d]	µmol/L	NR
	Diacyl-PC C36:0	20	4.74 (4.08-5.41)[d]	20	5.13 (4.66-5.61)[d]	µmol/L	NR
	Diacyl-PC C36:1	20	42.2 (37.9-49.5)[d]	20	46.5 (42.2-53.7)[d]	µmol/L	NR
	Diacyl-PC C36:2	20	146 (124-163)[d]	20	155 (140-173)[d]	µmol/L	NR
	Diacyl-PC C36:3	20	97.0 (87.6-104)[d]	20	99.0 (88.7-112)[d]	µmol/L	NR
	Diacyl-PC C36:4	20	135 (120-149)[d]	20	141 (119-171)[d]	µmol/L	NR
	Diacyl-PC C36:5	20	16.5 (12.2-21.7)[d]	20	20.2 (17.3-28.5)[d]	µmol/L	NR
	Diacyl-PC C36:6	20	0.750 (0.582-1.01)[d]	20	0.999 (0.785-1.19)[d]	µmol/L	NR
	Diacyl-PC C38:0	20	3.06 (2.59-3.38)[d]	20	3.54 (2.64-4.09)[d]	µmol/L	NR
	Diacyl-PC C38:1	20	0.904 (0.653-1.21)[d]	20	0.765 (0.610-0.973)[d]	µmol/L	NR
	Diacyl-PC C38:3	20	43.1 (37.3-51.8)[d]	20	44.8 (37.4-50.6)[d]	µmol/L	NR
	Diacyl-PC C38:4	20	80.0 (71.7-105)[d]	20	93.1 (72.8-111)[d]	µmol/L	NR
	Diacyl-PC C38:5	20	40.3 (33.1-46.2)[d]	20	47.6 (39.0-53.0)[d]	µmol/L	NR
	Diacyl-PC C38:6	20	59.1 (50.9-75.9)[d]	20	71.4 (61.1-82.5)[d]	µmol/L	NR
	Diacyl-PC C40:2	20	0.787 (0.494-1.05)[d]	20	0.361 (0.281-0.665)[d]	µmol/L	NR
	Diacyl-PC C40:3	20	1.00 (0.786-1.28)[d]	20	0.694 (0.585-1.02)[d]	µmol/L	NR
	Diacyl-PC C40:4	20	3.38 (2.95-4.28)[d]	20	3.34 (2.82-3.79)[d]	µmol/L	NR

Ref.	Metabolite	Cancer cases		Controls		Unit	p-value
		No.	Metabolite conc.	No.	Metabolite conc.		
Klupczynska 2019 [45] (continued)	Diacyl-PC C40:5	20	7.90 (6.24-10.1)[d]	20	9.13 (7.21-9.54)[d]	µmol/L	NR
	Diacyl-PC C40:6	20	22.8 (17.3-28.1)[d]	20	23.9 (21.4-31.0)[d]	µmol/L	NR
	Diacyl-PC C42:0	20	0.521 (0.435-0.686)[d]	20	0.550 (0.393-0.720)[d]	µmol/L	NR
	Diacyl-PC C42:1	20	0.291 (0.230-0.371)[d]	20	0.272 (0.221-0.343)[d]	µmol/L	NR
	Diacyl-PC C42:2	20	0.321 (0.266-0.414)[d]	20	0.287 (0.216-0.334)[d]	µmol/L	NR
	Diacyl-PC C42:4	20	0.389 (0.265-0.585)[d]	20	0.207 (0.176-0.356)[d]	µmol/L	NR
	Diacyl-PC C42:5	20	0.368 (0.341-0.500)[d]	20	0.337 (0.313-0.411)[d]	µmol/L	NR
	Acyl-alkyl-PC C30:0	20	0.359 (0.316-0.432)[d]	20	0.425 (0.299-0.497)[d]	µmol/L	NR
	Acyl-alkyl-PC C30:1	20	0.495 (0.407-0.573)[d]	20	0.494 (0.467-0.563)[d]	µmol/L	NR
	Acyl-alkyl-PC C32:1	20	2.12 (1.69-2.48)[d]	20	2.27 (1.74-2.47)[d]	µmol/L	NR
	Acyl-alkyl-PC C32:2	20	0.909 (0.781-1.04)[d]	20	1.01 (0.888-1.08)[d]	µmol/L	NR
	Acyl-alkyl-PC C34:0	20	1.27 (1.17-1.49)[d]	20	1.66 (1.31-1.95)[d]	µmol/L	NR
	Acyl-alkyl-PC C34:1	20	7.13 (6.06-8.05)[d]	20	7.90 (6.28-9.20)[d]	µmol/L	NR
	Acyl-alkyl-PC C34:2	20	6.77 (4.96-7.78)[d]	20	7.66 (6.39-9.36)[d]	µmol/L	NR
	Acyl-alkyl-PC C34:3	20	5.57 (3.99-7.16)[d]	20	5.90 (5.69-7.24)[d]	µmol/L	NR
	Acyl-alkyl-PC C36:0	20	1.46 (1.32-1.75)[d]	20	1.24 (0.988-1.50)[d]	µmol/L	NR
	Acyl-alkyl-PC C36:1	20	8.58 (7.24-10.5)[d]	20	8.63 (6.52-9.44)[d]	µmol/L	NR
	Acyl-alkyl-PC C36:2	20	7.81 (6.58-8.79)[d]	20	9.53 (8.75-10.8)[d]	µmol/L	NR
	Acyl-alkyl-PC C36:3	20	5.60 (4.61-6.75)[d]	20	6.65 (5.51-7.88)[d]	µmol/L	NR
	Acyl-alkyl-PC C36:4	20	10.7 (9.13-13.0)[d]	20	12.8 (10.9-16.4)[d]	µmol/L	NR
	Acyl-alkyl-PC C36:5	20	20.1 (17.8-22.2)[d]	20	21.3 (18.5-23.6)[d]	µmol/L	NR
	Acyl-alkyl-PC C38:0	20	2.04 (1.76-2.48)[d]	20	2.21 (1.84-2.53)[d]	µmol/L	NR
	Acyl-alkyl-PC C38:1	20	2.45 (1.97-3.05)[d]	20	1.75 (1.14-2.75)[d]	µmol/L	NR

Ref.	Metabolite	Cancer cases		Controls		Unit	p-value
		No.	Metabolite conc.	No.	Metabolite conc.		
Klupczynska 2019 [45] (continued)	Acyl-alkyl-PC C38:2	20	2.99 (2.58-4.21)[d]	20	2.22 (1.79-3.37)[d]	µmol/L	NR
	Acyl-alkyl-PC C38:3	20	7.32 (5.55-9.42)[d]	20	4.85 (3.75-7.72)[d]	µmol/L	NR
	Acyl-alkyl-PC C38:4	20	10.2 (8.45-11.4)[d]	20	11.6 (9.83-13.1)[d]	µmol/L	NR
	Acyl-alkyl-PC C38:5	20	11.6 (9.88-13.8)[d]	20	13.8 (11.0-15.9)[d]	µmol/L	NR
	Acyl-alkyl-PC C38:6	20	5.57 (4.78-6.66)[d]	20	7.34 (5.64-8.13)[d]	µmol/L	NR
	Acyl-alkyl-PC C40:1	20	1.12 (0.96-1.36)[d]	20	1.19 (1.10-1.32)[d]	µmol/L	NR
	Acyl-alkyl-PC C40:2	20	2.34 (2.02-2.80)[d]	20	2.31 (1.87-2.58)[d]	µmol/L	NR
	Acyl-alkyl-PC C40:3	20	3.39 (2.16-4.63)[d]	20	1.27 (1.08-3.30)[d]	µmol/L	NR
	Acyl-alkyl-PC C40:4	20	3.11 (2.42-4.37)[d]	20	2.33 (1.97-3.44)[d]	µmol/L	NR
	Acyl-alkyl-PC C40:5	20	4.34 (3.34-5.61)[d]	20	3.72 (3.20-4.44)[d]	µmol/L	NR
	Acyl-alkyl-PC C40:6	20	3.40 (3.00-4.12)[d]	20	4.22 (3.42-5.28)[d]	µmol/L	NR
	Acyl-alkyl-PC C42:1	20	0.429 (0.385-0.562)[d]	20	0.297 (0.268-0.417)[d]	µmol/L	NR
	Acyl-alkyl-PC C42:2	20	0.586 (0.538-0.801)[d]	20	0.571 (0.524-0.617)[d]	µmol/L	NR
	Acyl-alkyl-PC C42:3	20	0.828 (0.723-1.18)[d]	20	0.793 (0.749-0.864)[d]	µmol/L	NR
	Acyl-alkyl-PC C42:4	20	0.559 (0.444-0.894)[d]	20	0.481 (0.337-0.716)[d]	µmol/L	NR
	Acyl-alkyl-PC C44:3	20	0.209 (0.163-0.301)[d]	20	0.142 (0.122-0.184)[d]	µmol/L	NR
	Acyl-alkyl-PC C44:4	20	0.434 (0.384-0.562)[d]	20	0.418 (0.380-0.501)[d]	µmol/L	NR
	Acyl-alkyl-PC C44:5	20	1.45 (1.23-1.84)[d]	20	1.53 (1.26-2.04)[d]	µmol/L	NR
	Acyl-alkyl-PC C44:6	20	0.930 (0.804-1.23)[d]	20	1.13 (0.812-1.27)[d]	µmol/L	NR
	SM (OH) C14:1	20	2.89 (1.61-3.60)[d]	20	3.38 (2.64-4.64)[d]	µmol/L	NR
	SM (OH) C16:1	20	2.41 (1.79-2.98)[d]	20	3.01 (2.35-3.35)[d]	µmol/L	NR
	SM (OH) C22:1	20	10.2 (9.15-11.0)[d]	20	12.3 (10.3-13.6)[d]	µmol/L	NR
	SM (OH) C22:2	20	9.66 (8.09-11.2)[d]	20	11.4 (9.77-12.1)[d]	µmol/L	NR
	SM (OH) C24:1	20	1.22 (1.09-1.31)[d]	20	1.22 (1.11-1.40)[d]	µmol/L	NR

Ref.	Metabolite	Cancer cases		Controls		Unit	p-value
		No.	Metabolite conc.	No.	Metabolite conc.		
Klupczynska 2019 [45] (continued)	SM C16:0	20	90.0 (80.7-105)[d]	20	89.3 (81.6-98.4)[d]	μmol/L	NR
	SM C16:1	20	13.1 (11.4-14.7)[d]	20	12.6 (11.4-14.5)[d]	μmol/L	NR
	SM C18:0	20	22.5 (17.1-23.8)[d]	20	21.7 (18.0-25.5)[d]	μmol/L	NR
	SM C18:1	20	11.7 (8.68-14.1)[d]	20	10.0 (8.88-13.7)[d]	μmol/L	NR
	SM C20:2	20	0.839 (0.751-1.052)[d]	20	0.978 (0.810-1.05)[d]	μmol/L	NR
	SM C22:3	20	6.81 (5.60-7.73)[d]	20	8.29 (6.88-10.9)[d]	μmol/L	NR
	SM C24:0	20	22.0 (20.3-24.5)[d]	20	23.1 (19.6-25.8)[d]	μmol/L	NR
	SM C24:1	20	58.3 (51.1-69.5)[d]	20	54.4 (48.9-63.4)[d]	μmol/L	NR
	SM C26:0	20	0.182 (0.162-0.198)[d]	20	0.167 (0.134-0.213)[d]	μmol/L	NR
	SM C26:1	20	0.504 (0.343-0.543)[d]	20	0.462 (0.375-0.552)[d]	μmol/L	NR
Tumor tissue							
Kukreja 1982 [51]	PGE	8	258.3 ± 224.1[a]	8	61.1 ± 43.2[a]	pg/mg tissue	NR
	PGE_2	6	51.8 ± 19.3[a]	6	19.0 ± 12.1[a] (ND: 1)[b]	pg/mg tissue	NR
Kami 2013 [50]	3-Phosphoglycerate	9	145.59 ± 136.45[a]	9	284.63 ± 135.29	nmol/g tissue	NR
	ADP	9	153.82 ± 90.59[a]	9	64.45 ± 52.27[a]	nmol/g tissue	NR
	AMP	9	363.19 ± 177.99[a] (ND: 2)[b]	9	45.99 ± 74.34[a] (ND:3)[b]	nmol/g tissue	NR
	ATP	9	67.07 ± 55.8	9	17.5 ± 17.26[a] (ND: 1)[b]	nmol/g tissue	NR
	Alanine	9	1273.08 ± 491.2[a]	9	783.83 ± 166.14[a]	nmol/g tissue	NR
	Arginine	9	478.51 ± 159.47[a]	9	395.57 ± 128.6[a]	nmol/g tissue	NR

Ref.	Metabolite	Cancer cases		Controls		Unit	p-value
		No.	Metabolite conc.	No.	Metabolite conc.		
Kami 2013 [50] (continued)	Asparagine	9	285.51 ± 112.58[a]	9	132.85 ± 31.93[a]	nmol/g tissue	NR
	Aspartic acid	9	760.48 ± 311.02[a]	9	715.89 ± 267.35[a]	nmol/g tissue	NR
	cis-aconitate	9	- (ND: 9)[b]	9	1.19 ± 0.66[a] (ND: 4)[b]	nmol/g tissue	NR
	Citrate	9	195.98 ± 60.39[a]	9	203.31 ± 51.86[a]	nmol/g tissue	NR
	Dihydroxyacetone Phosphate	9	31.68 ± 21.26[a]	9	57.29 ± 30.12[a]	nmol/g tissue	NR
	Fructose 1,6-Bisphosphate	9	175.33 ± 177.59[a]	9	19.67 ± 10.91[a] (ND: 2)[b]	nmol/g tissue	NR
	Fructose 6-Phosphate	9	18.53 ± 8.19[a] (ND: 5)[b]	9	59.28 ± 41.36[a]	nmol/g tissue	NR
	Fumarate	9	143.89 ± 70.29[a]	9	56.71 ± 21.89[a]	nmol/g tissue	NR
	Glucose-6-Phosphate	9	106.08 ± 72.41[a]	9	237.4 ± 162.68[a]	nmol/g tissue	NR
	Glutamic Acid	9	3573.49 ± 1141.71[a]	9	2206.62 ± 822.43[a]	nmol/g tissue	NR
	Glutamine	9	1888.54 ± 524.24[a]	9	1004.86 ± 302.96[a]	nmol/g tissue	NR
	Glycine	9	2086.53 ± 644.81[a]	9	1437.56 ± 481.76[a]	nmol/g tissue	NR
	GDP	9	68.97 ± 37.9[a]	9	35.77 ± 45.75[a]	nmol/g tissue	NR
	GMP	9	228.61 ± 137.86[a]	9	38.72 ± 36.9[a] (ND:1)[b]	nmol/g tissue	NR
	GTP	9	15.33 ± 12.69[a]	9	3.61 ± 1.76[a] (ND:3)[b]	nmol/g tissue	NR

Ref.	Metabolite	Cancer cases		Controls		Unit	p-value
		No.	Metabolite conc.	No.	Metabolite conc.		
Kami 2013 [50] (continued)	Histidine	9	242.28 ± 73.74[a]	9	138.14 ± 17.32[a]	nmol/g tissue	NR
	Isocitrate	9	4.22 ± 1.43[a]	9	5.5 ± 3.77[a]	nmol/g tissue	NR
	Isoleucine	9	258.6 ± 79.99[a]	9	143.51 ± 34.22[a]	nmol/g tissue	NR
	Lactate	9	23203.52 ± 8530.5[a]	9	7513.84 ± 3711.68[a] (ND: 1)[b]	nmol/g tissue	NR
	Leucine	9	606.64 ± 181.65[a]	9	385.23 ± 88.95[a]	nmol/g tissue	NR
	Lysine	9	740.03 ± 196.15[a]	9	693.35 ± 174.16[a]	nmol/g tissue	NR
	Malate	9	569.51 ± 243.37[a]	9	253.98 ± 73.3[a]	nmol/g tissue	NR
	Methionine	9	130.35 ± 52.73[a]	9	78.21 ± 22.19[a]	nmol/g tissue	NR
	Phenylalanine	9	259.61 ± 79.34[a]	9	173.41 ± 56.45[a]	nmol/g tissue	NR
	Phosphoenolpyruvate	9	24 ± 46.31[a] (ND: 1)[b]	9	88.84 ± 46.38[a]	nmol/g tissue	NR
	Proline	9	698.56 ± 264.28[a]	9	345.4 ± 65.44[a]	nmol/g tissue	NR
	Pyruvate	9	52.97 ± 24.71[a]	9	21.94 ± 6.65[a] (ND: 1)[b]	nmol/g tissue	NR
	Serine	9	868.18 ± 320.96[a]	9	615.82 ± 179.62[a]	nmol/g tissue	NR
	Succinate	9	169.04 ± 130.52[a]	9	92.88 ± 74.89[a]	nmol/g tissue	NR
	Threonine	9	490.43 ± 156.38[a]	9	293.94 ± 88.94[a]	nmol/g tissue	NR

Ref.	Metabolite	Cancer cases		Controls		Unit	p-value
		No.	Metabolite conc.	No.	Metabolite conc.		
Kami 2013 [50] (continued)	Tryptophan	9	37.48 ± 10.8[a]	9	22.37 ± 8.86[a] (ND: 1)[b]	nmol/g tissue	NR
	Tyrosine	9	267.7 ± 88.08[a]	9	175.84 ± 63.09[a]	nmol/g tissue	NR
	Valine	9	605.28 ± 138.22[a]	9	372.57 ± 56.74[a]	nmol/g tissue	NR
			Urine				
Zhang 2006 [53]	NNAL	10	0.685 ± 1.516[a]	8	0.174 ± 0.136[a]	µg/L	NR
	NNAL-O-Gluc	10	0.349 ± 0.282[a]	8	0.862 ± 1.657[a]	µg/L	NR
Hwang 2014 [54]	Total NNAL (NNAL + NNAL-N-Gluc + NNAL-O-Gluc)	74	4.7 ± 15[a]	85	6.5 ±1 7.9[a]	pg/mg	<0.001
Kawamoto 2019 [55]	PGE-MUM	54	22.4 ± 11.9[a]	124	15.4 ± 8.3	µg/g Cr	<0.05

Cr Creatinine, *ND* Not detected, *NR* Not reported, *NS* Not significant.

Refer to Supplementary material List 1 for the name of abbreviated metabolites.

[a] Data is reported as mean ± SD

[b] For studies where the reported metabolites were not detected in some of their participants, the number of sample is reported as ND

[c] Data is reported as mean (95% CI), where CI were calculated from the square root of the data

[d] Data is reported as median (inter-quartile range)

[e] The lung cancer group were diagnosed with epidermoid carcinoma

[f] The lung cancer group were diagnosed with SCLC

[g] The lung cancer group were diagnosed with adenocarcinoma

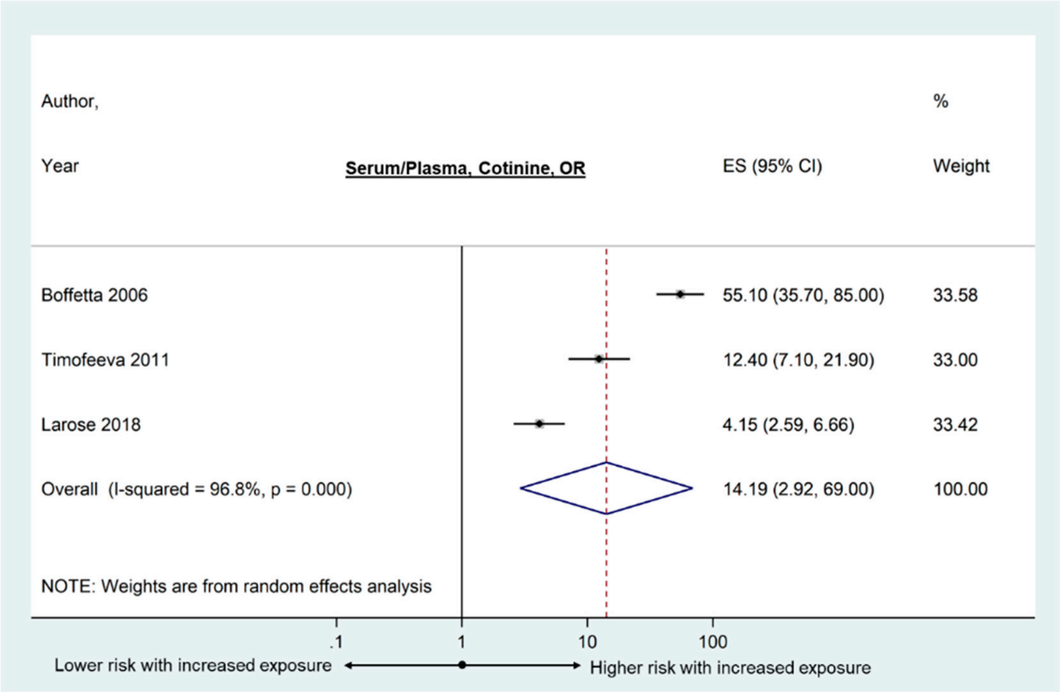
Author,
Year
%
Weight
Serum/Plasma, Cotinine, OR
ES (95% CI)
Boffetta 2006
55.10 (35.70, 85.00)
33.58
Timofeeva 2011
12.40 (7.10, 21.90)
33.00
Larose 2018
4.15 (2.59, 6.66)
33.42
Overall (I-squared = 96.8%, p = 0.000)
14.19 (2.92, 69.00)
100.00
NOTE: Weights are from random effects analysis
.1
1
10
100
Lower risk with increased exposure
Higher risk with increased exposure

(**a**)

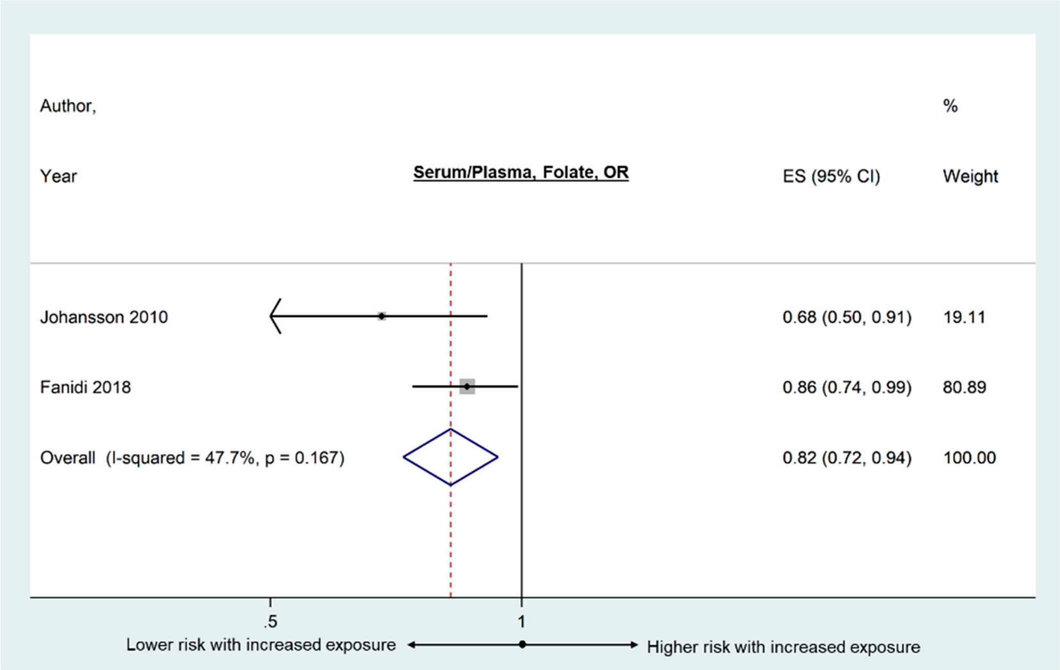
Author,
Year
%
Weight
Serum/Plasma, Folate, OR
ES (95% CI)
Johansson 2010
0.68 (0.50, 0.91)
19.11
Fanidi 2018
0.86 (0.74, 0.99)
80.89
Overall (I-squared = 47.7%, p = 0.167)
0.82 (0.72, 0.94)
100.00
.5
1
Lower risk with increased exposure
Higher risk with increased exposure

(**b**)

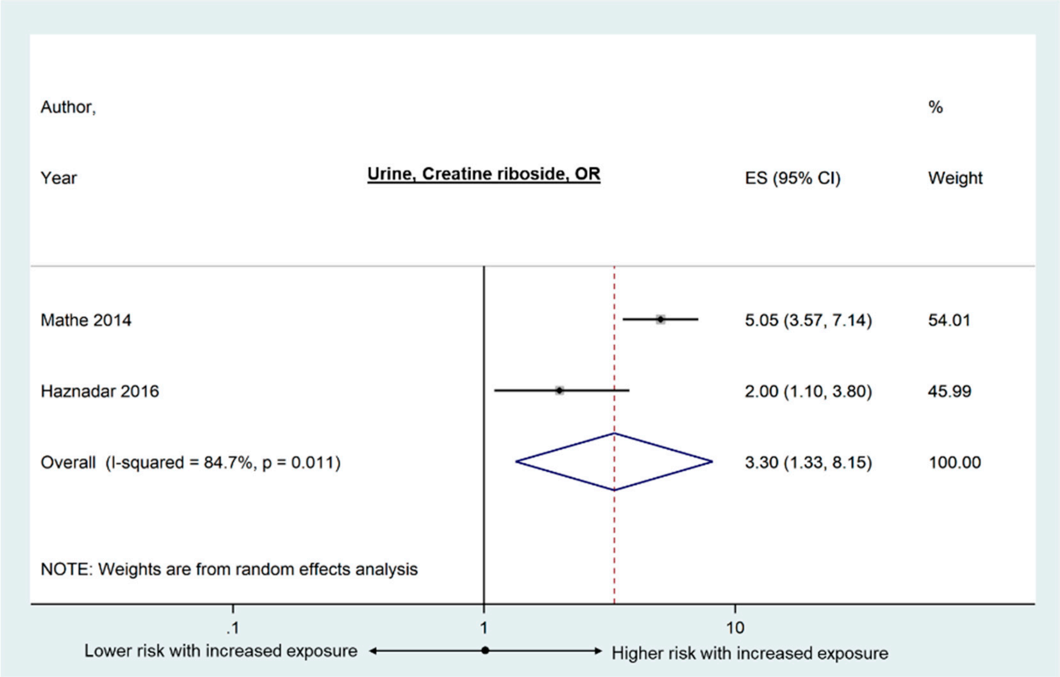

Author,
Year
Urine, Creatine riboside, OR
%
ES (95% CI)
Weight
Mathe 2014
5.05 (3.57, 7.14)
54.01
Haznadar 2016
2.00 (1.10, 3.80)
45.99
Overall (I-squared = 84.7%, p = 0.011)
3.30 (1.33, 8.15)
100.00
NOTE: Weights are from random effects analysis
.1
1
10
Lower risk with increased exposure
Higher risk with increased exposure

(**c**)

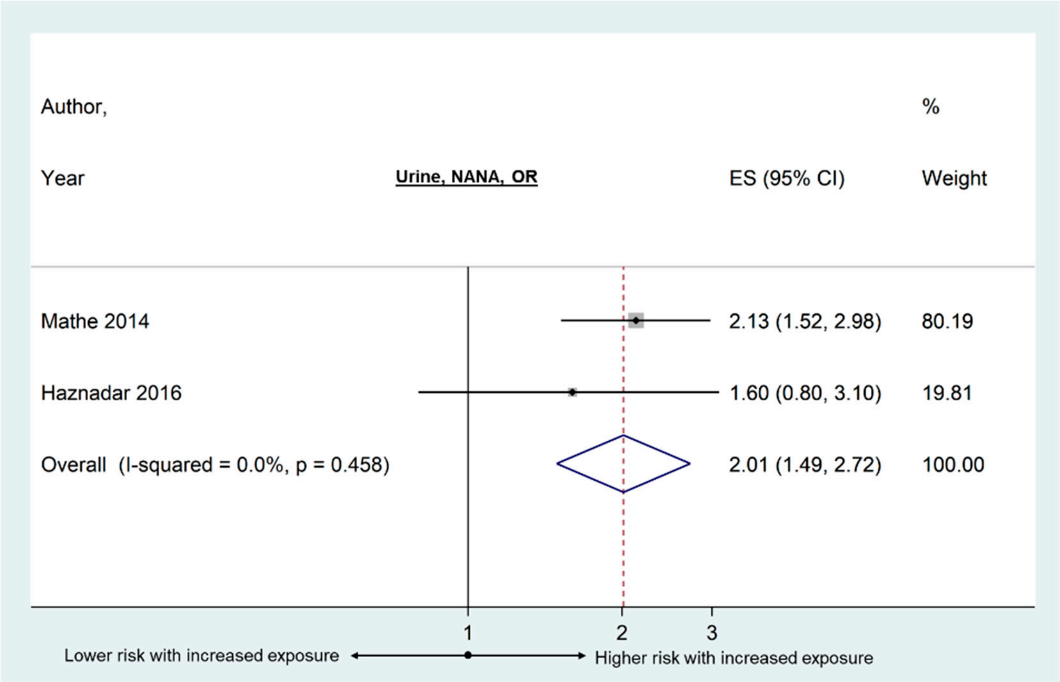

Author,
Year
Urine, NANA, OR
%
ES (95% CI)
Weight
Mathe 2014
2.13 (1.52, 2.98)
80.19
Haznadar 2016
1.60 (0.80, 3.10)
19.81
Overall (I-squared = 0.0%, p = 0.458)
2.01 (1.49, 2.72)
100.00
1
2
3
Lower risk with increased exposure
Higher risk with increased exposure

(**d**)

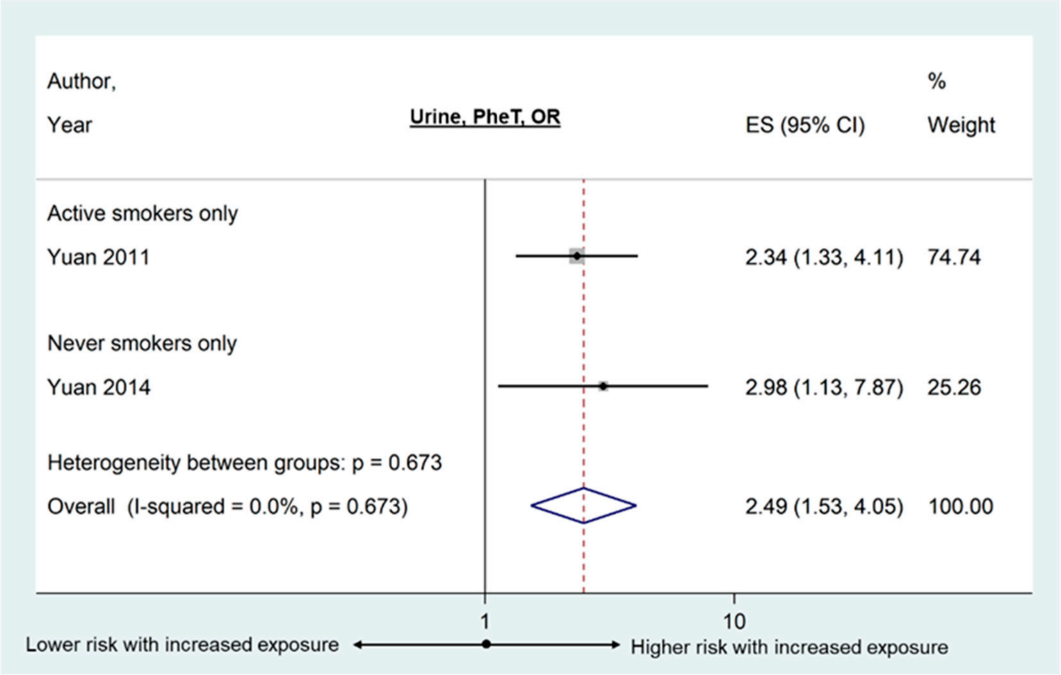

Author,
Year
Urine, PheT, OR
ES (95% CI)
%
Weight
Active smokers only
Yuan 2011
2.34 (1.33, 4.11)
74.74
Never smokers only
Yuan 2014
2.98 (1.13, 7.87)
25.26
Heterogeneity between groups: p = 0.673
Overall (I-squared = 0.0%, p = 0.673)
2.49 (1.53, 4.05)
100.00
1
10
Lower risk with increased exposure
Higher risk with increased exposure

(**e**)

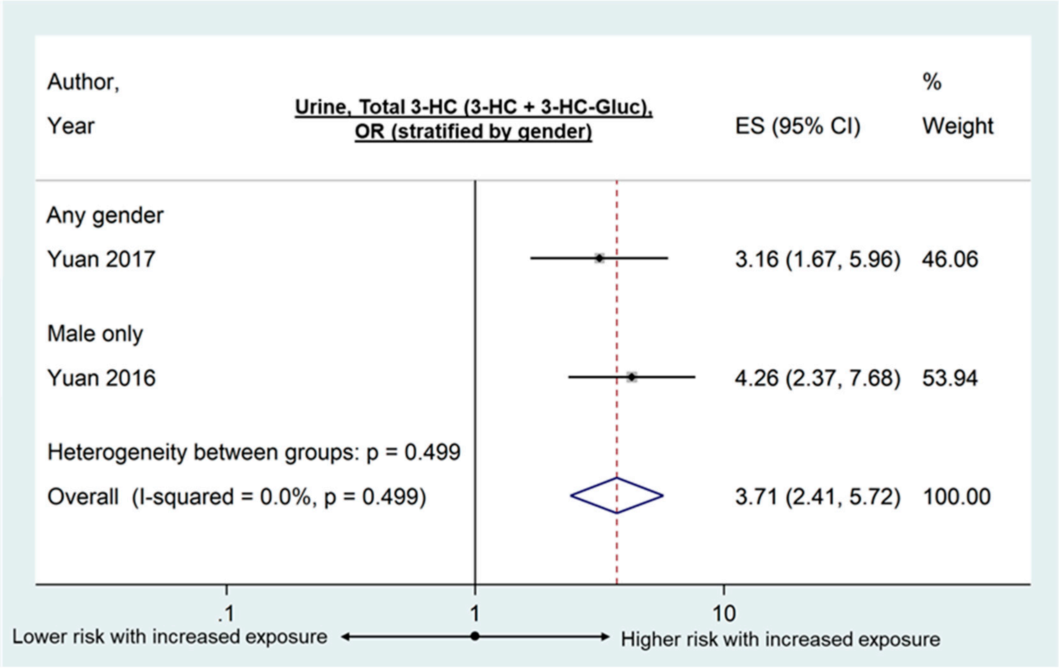

Author,
Year
Urine, Total 3-HC (3-HC + 3-HC-Gluc),
OR (stratified by gender)
ES (95% CI)
%
Weight
Any gender
Yuan 2017
3.16 (1.67, 5.96)
46.06
Male only
Yuan 2016
4.26 (2.37, 7.68)
53.94
Heterogeneity between groups: p = 0.499
Overall (I-squared = 0.0%, p = 0.499)
3.71 (2.41, 5.72)
100.00
.1
1
10
Lower risk with increased exposure
Higher risk with increased exposure

(**f**)

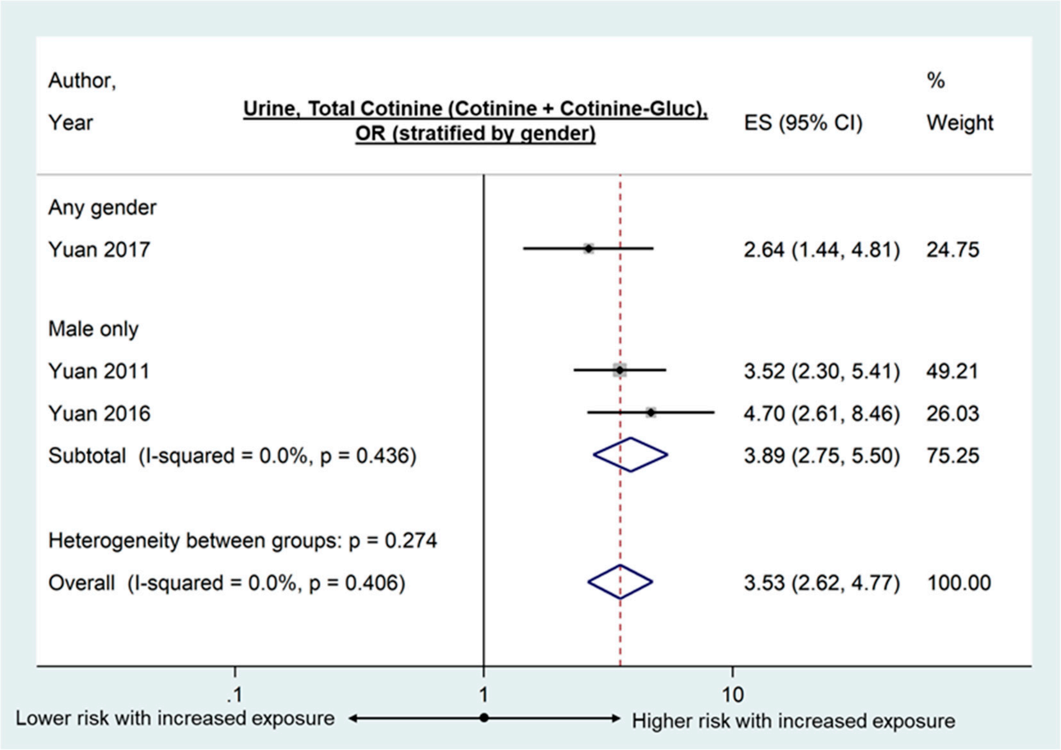

Author,
Year
Urine, Total Cotinine (Cotinine + Cotinine-Gluc), OR (stratified by gender)
ES (95% CI)
%
Weight
Any gender
Yuan 2017
2.64 (1.44, 4.81)
24.75
Male only
Yuan 2011
3.52 (2.30, 5.41)
49.21
Yuan 2016
4.70 (2.61, 8.46)
26.03
Subtotal (I-squared = 0.0%, p = 0.436)
3.89 (2.75, 5.50)
75.25
Heterogeneity between groups: p = 0.274
Overall (I-squared = 0.0%, p = 0.406)
3.53 (2.62, 4.77)
100.00
.1
1
10
Lower risk with increased exposure
Higher risk with increased exposure

(**g**)

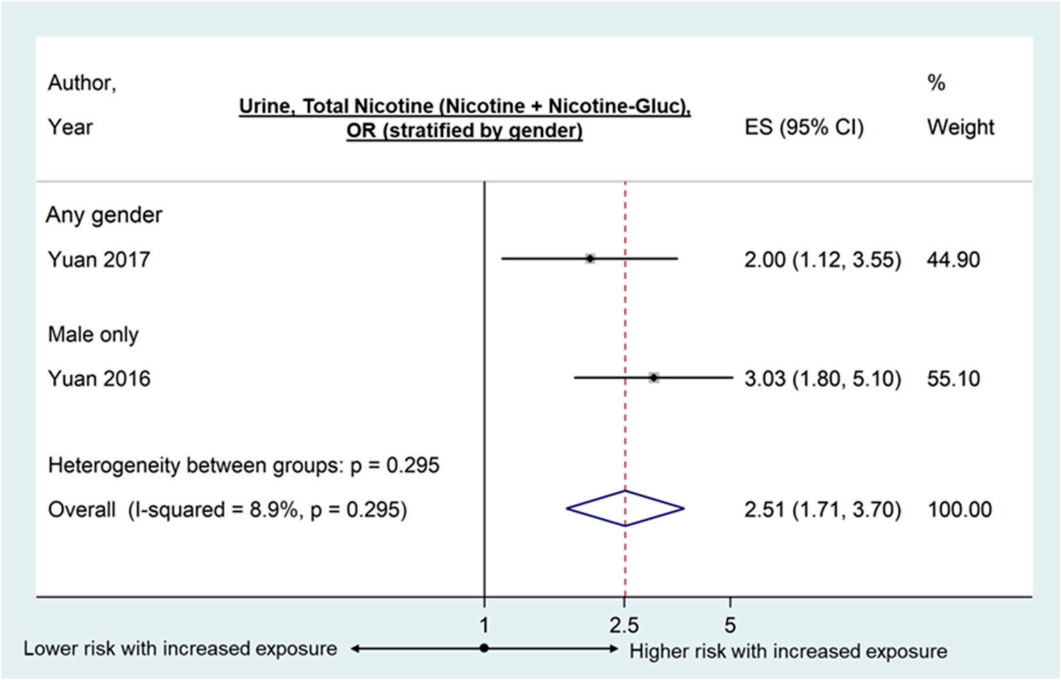

Author,
Year
Urine, Total Nicotine (Nicotine + Nicotine-Gluc), OR (stratified by gender)
ES (95% CI)
%
Weight
Any gender
Yuan 2017
2.00 (1.12, 3.55)
44.90
Male only
Yuan 2016
3.03 (1.80, 5.10)
55.10
Heterogeneity between groups: p = 0.295
Overall (I-squared = 8.9%, p = 0.295)
2.51 (1.71, 3.70)
100.00
1
2.5
5
Lower risk with increased exposure
Higher risk with increased exposure

(**h**)

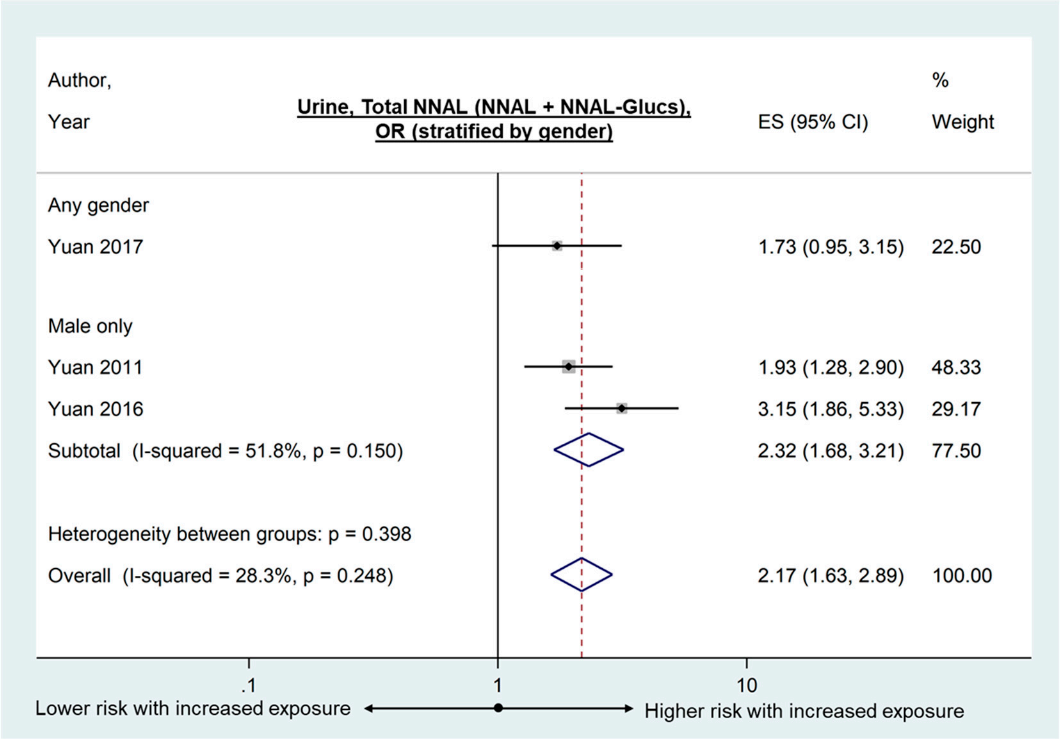

(i)

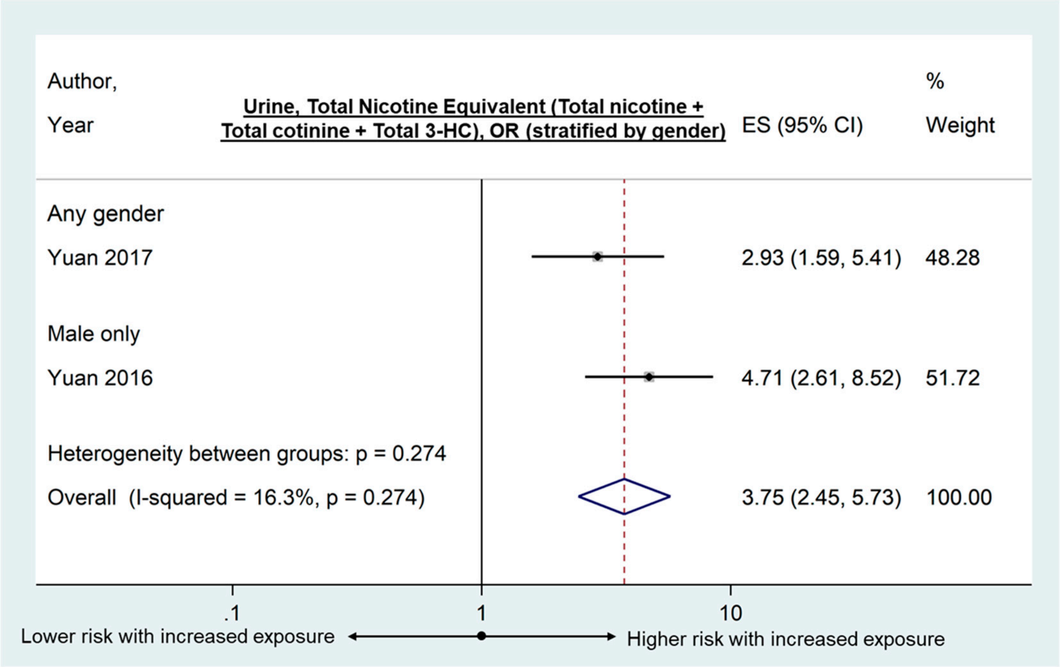

(j)

Figure S1. Effect size (ES; i.e. odds ratio (OR)) and 95% confidence interval (CI) of the association of lung cancer in relation to metabolites that achieved statistical significance.

The metabolites are (a) serum/plasma cotinine, (b) serum/plasma folate, (c) urinary creatine riboside, (d) urinary NANA, (e) urinary PheT, (f) urinary total 3-HC, (g) urinary total cotinine, (h) urinary total nicotine, (i) urinary total NNAL and (j) urinary TNE. Refer to Supplementary material List 1 for the name of abbreviated metabolites.

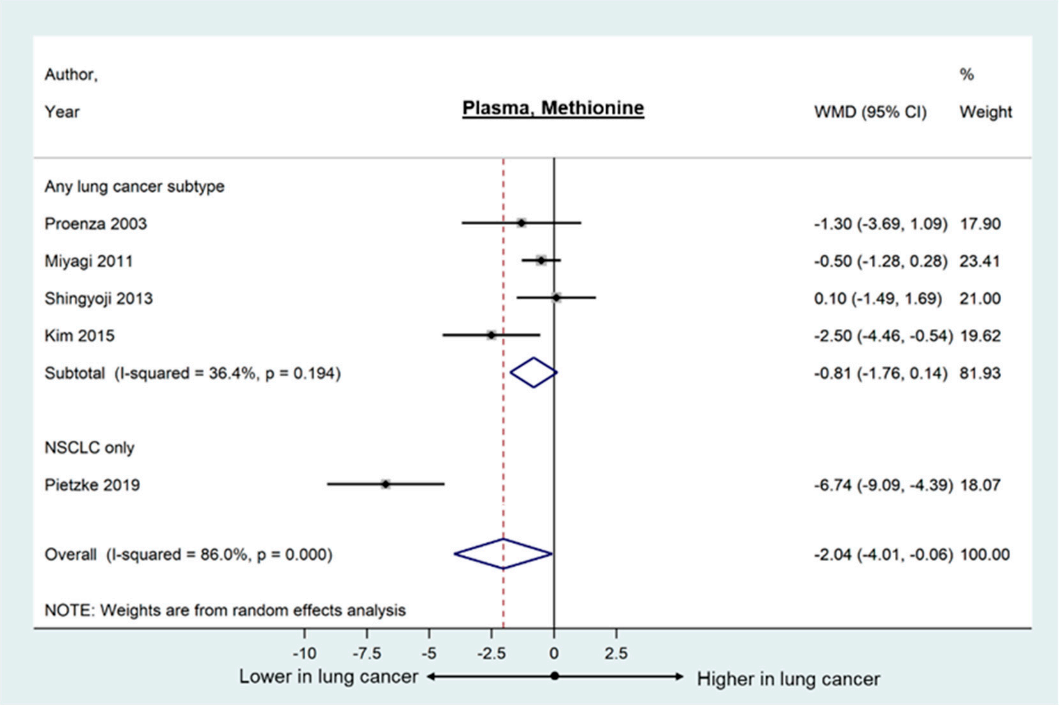
Author,
Year
Plasma, Methionine
WMD (95% CI)
% Weight
Any lung cancer subtype
Proenza 2003 -1.30 (-3.69, 1.09) 17.90
Miyagi 2011 -0.50 (-1.28, 0.28) 23.41
Shingyoji 2013 0.10 (-1.49, 1.69) 21.00
Kim 2015 -2.50 (-4.46, -0.54) 19.62
Subtotal (I-squared = 36.4%, p = 0.194) -0.81 (-1.76, 0.14) 81.93
NSCLC only
Pietzke 2019 -6.74 (-9.09, -4.39) 18.07
Overall (I-squared = 86.0%, p = 0.000) -2.04 (-4.01, -0.06) 100.00
NOTE: Weights are from random effects analysis
-10
-7.5
-5
-2.5
0
2.5
Lower in lung cancer
Higher in lung cancer

(**a**)

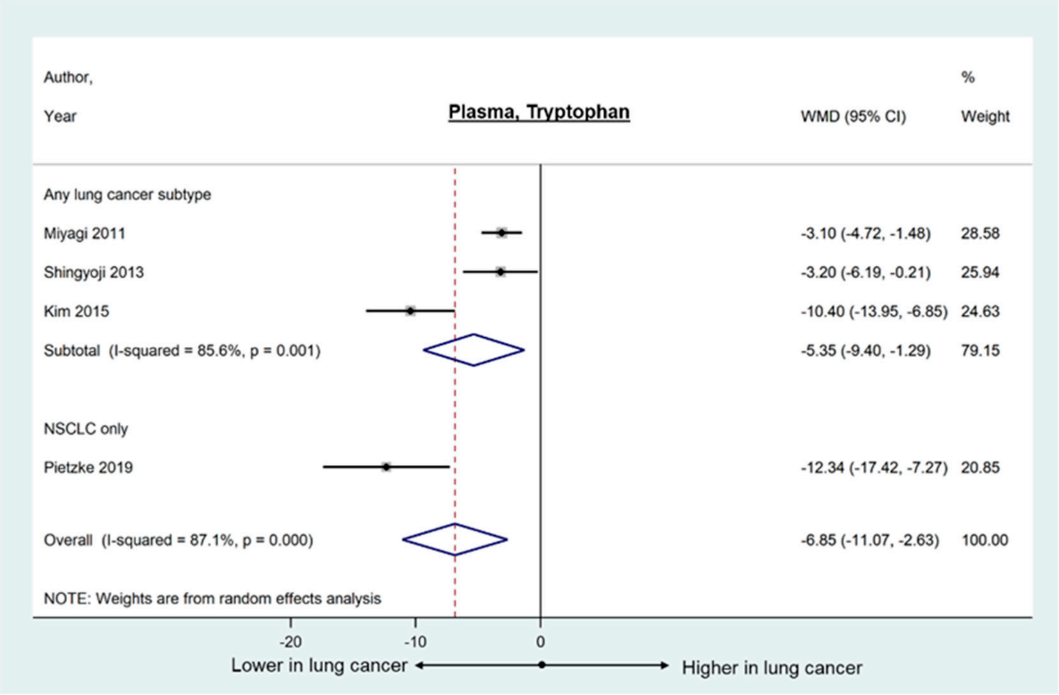
Author,
Year
Plasma, Tryptophan
WMD (95% CI)
% Weight
Any lung cancer subtype
Miyagi 2011 -3.10 (-4.72, -1.48) 28.58
Shingyoji 2013 -3.20 (-6.19, -0.21) 25.94
Kim 2015 -10.40 (-13.95, -6.85) 24.63
Subtotal (I-squared = 85.6%, p = 0.001) -5.35 (-9.40, -1.29) 79.15
NSCLC only
Pietzke 2019 -12.34 (-17.42, -7.27) 20.85
Overall (I-squared = 87.1%, p = 0.000) -6.85 (-11.07, -2.63) 100.00
NOTE: Weights are from random effects analysis
-20
-10
0
Lower in lung cancer
Higher in lung cancer

(**b**)

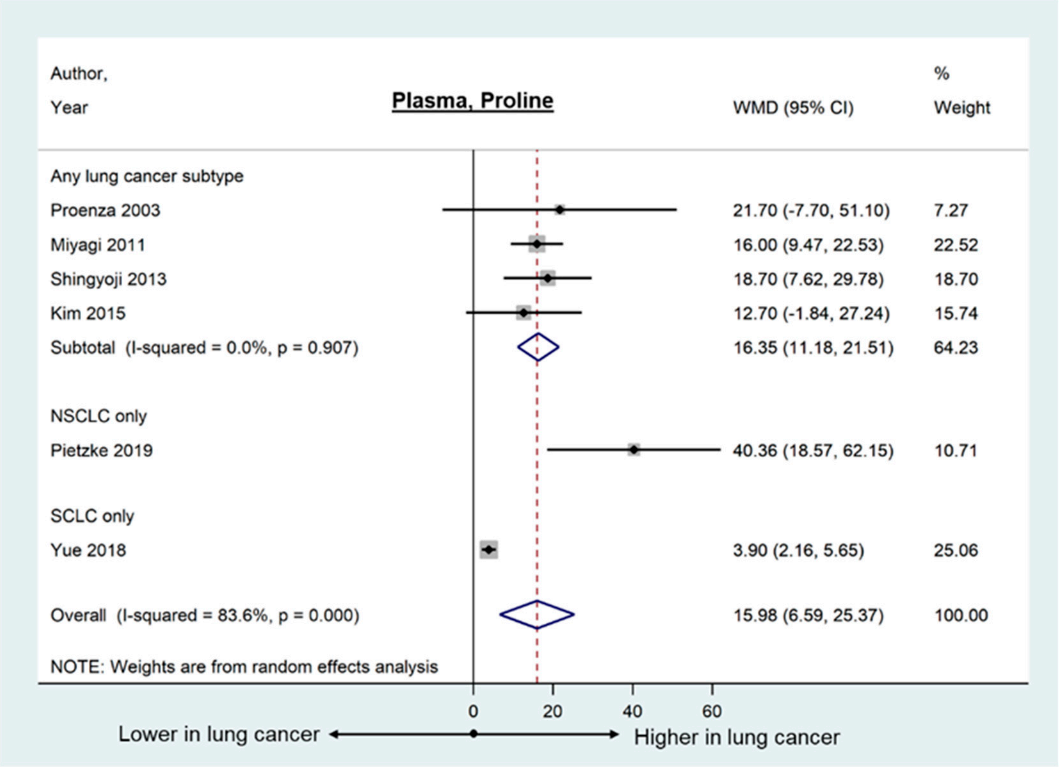

(**c**)

Figure S2. Weighted mean difference (WMD) and 95% confidence interval (CI) of the plasma metabolite concentration between lung cancer patients and healthy controls using a random-effects model, for metabolites that achieved statistical significance.

The metabolites are (a) methionine, (b) tryptophan and (c) proline. Data are presented as μmol/L.

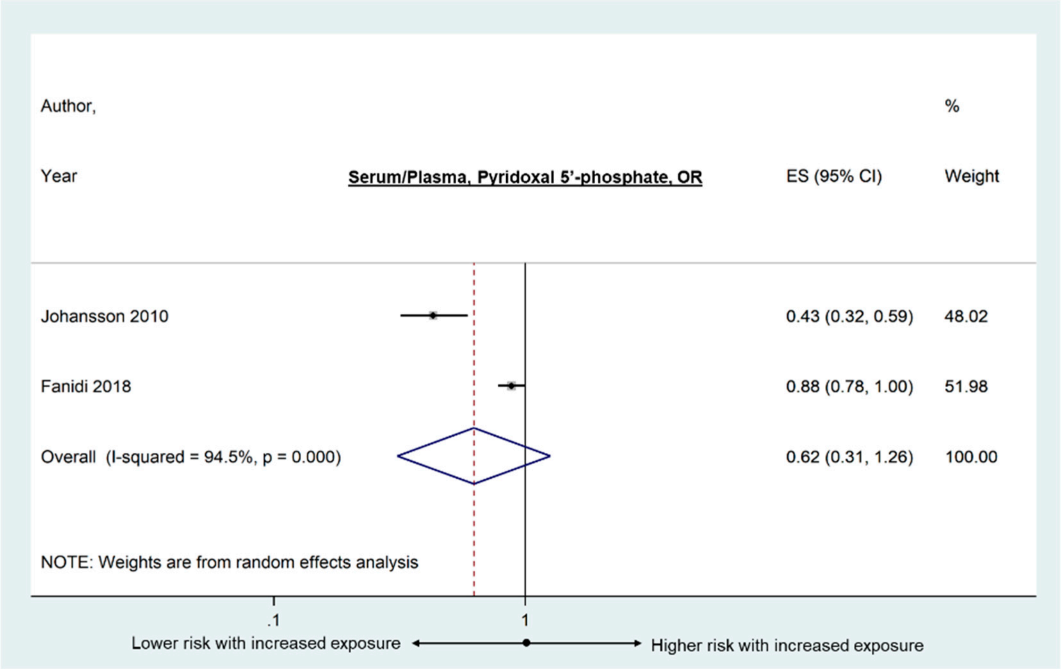

Author,
Year
%
Serum/Plasma, Pyridoxal 5'-phosphate, OR
ES (95% CI)
Weight
Johansson 2010
0.43 (0.32, 0.59)
48.02
Fanidi 2018
0.88 (0.78, 1.00)
51.98
Overall (I-squared = 94.5%, p = 0.000)
0.62 (0.31, 1.26)
100.00
NOTE: Weights are from random effects analysis
.1
1
Lower risk with increased exposure
Higher risk with increased exposure

(**a**)

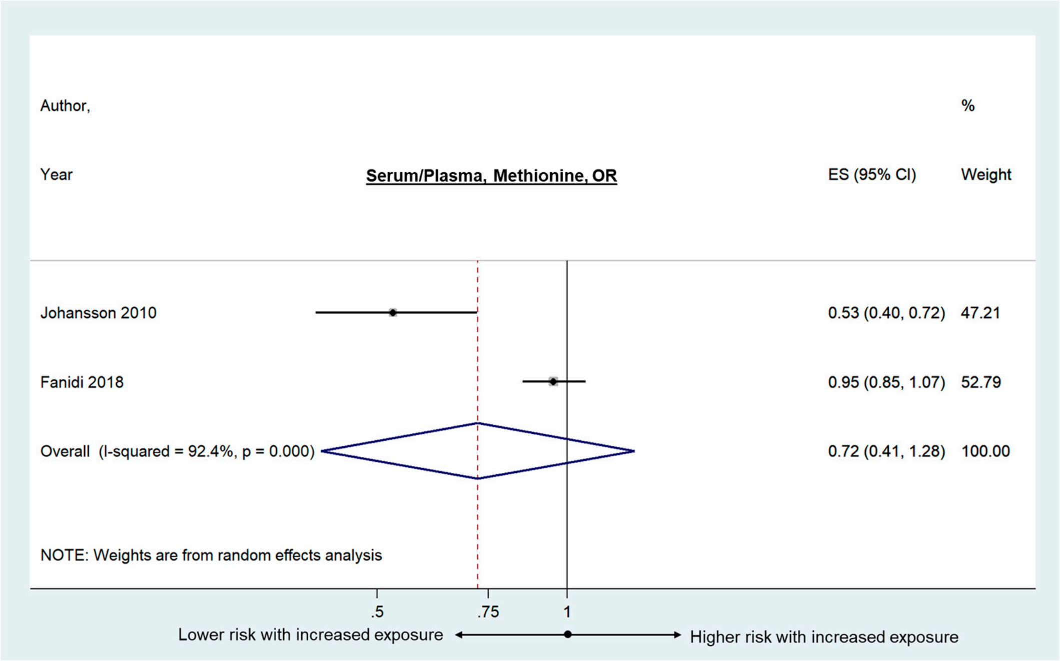

Author,
Year
%
Serum/Plasma, Methionine, OR
ES (95% CI)
Weight
Johansson 2010
0.53 (0.40, 0.72)
47.21
Fanidi 2018
0.95 (0.85, 1.07)
52.79
Overall (I-squared = 92.4%, p = 0.000)
0.72 (0.41, 1.28)
100.00
NOTE: Weights are from random effects analysis
.5
.75
1
Lower risk with increased exposure
Higher risk with increased exposure

(**b**)

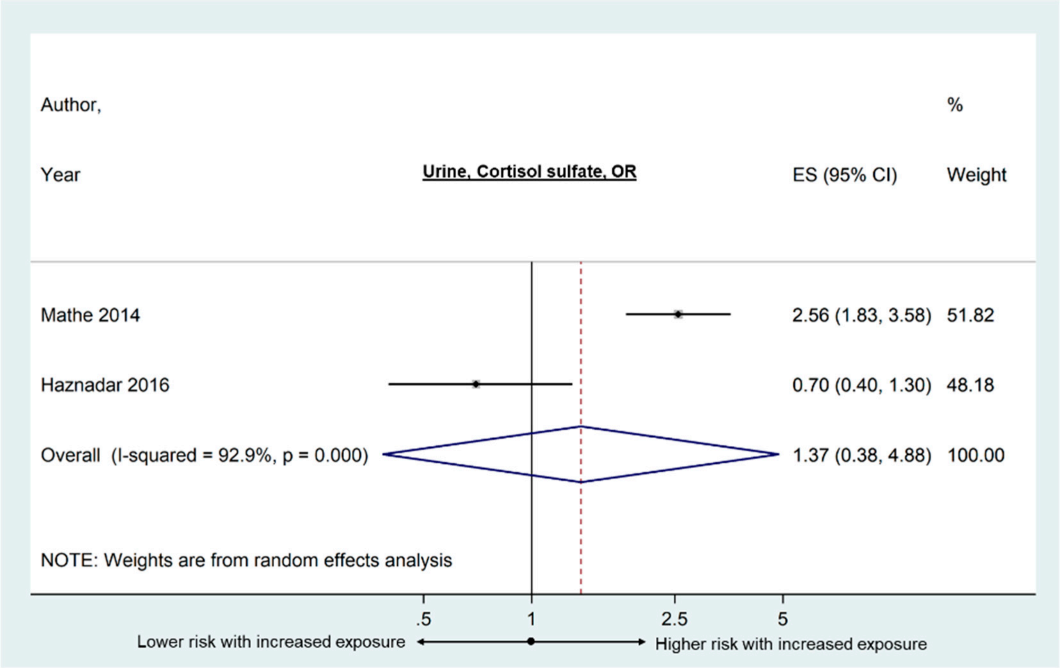
Author,
Year
Urine, Cortisol sulfate, OR
ES (95% CI)
%
Weight
Mathe 2014
2.56 (1.83, 3.58)
51.82
Haznadar 2016
0.70 (0.40, 1.30)
48.18
Overall (I-squared = 92.9%, p = 0.000)
1.37 (0.38, 4.88)
100.00
NOTE: Weights are from random effects analysis
.5
1
2.5
5
Lower risk with increased exposure
Higher risk with increased exposure

(**c**)

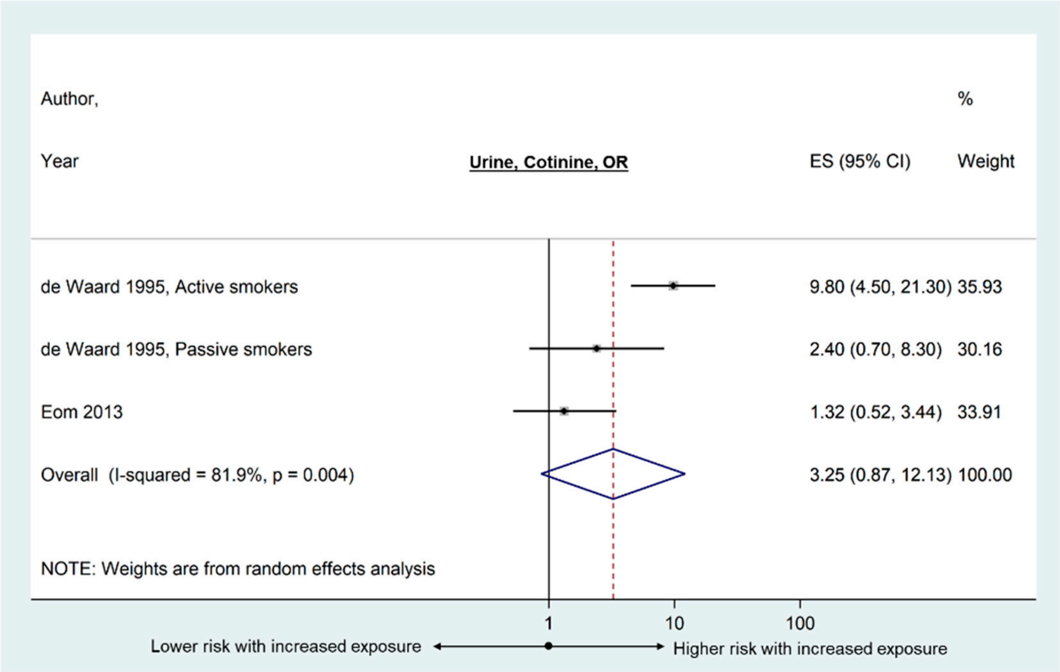
Author,
Year
Urine, Cotinine, OR
ES (95% CI)
%
Weight
de Waard 1995, Active smokers
9.80 (4.50, 21.30)
35.93
de Waard 1995, Passive smokers
2.40 (0.70, 8.30)
30.16
Eom 2013
1.32 (0.52, 3.44)
33.91
Overall (I-squared = 81.9%, p = 0.004)
3.25 (0.87, 12.13)
100.00
NOTE: Weights are from random effects analysis
1
10
100
Lower risk with increased exposure
Higher risk with increased exposure

(**d**)

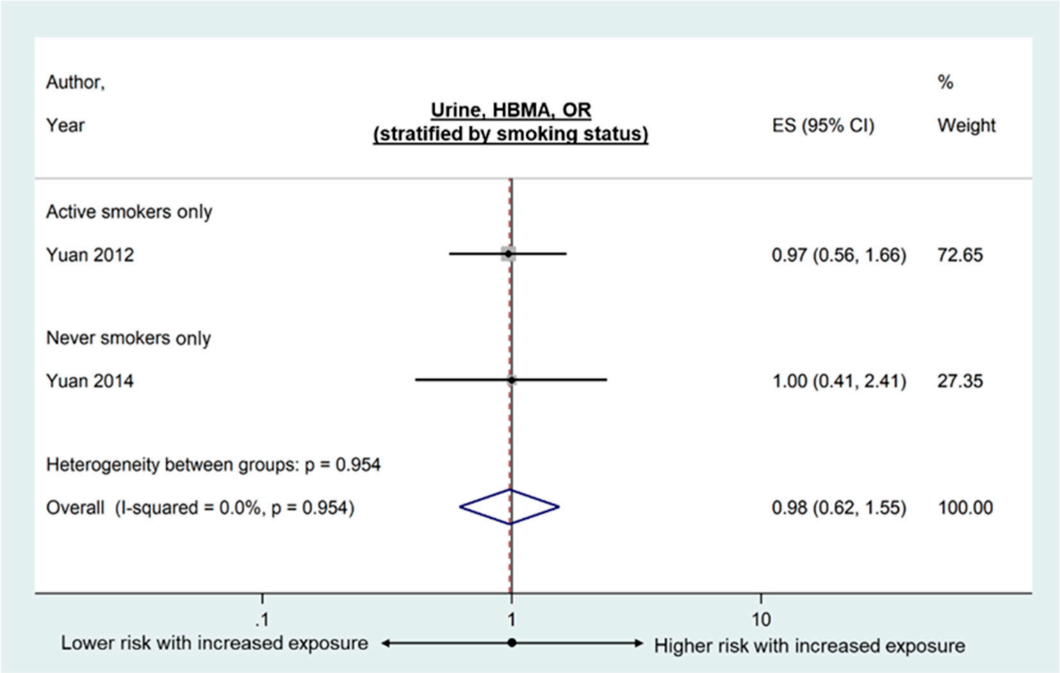
Author,
Year
Urine, HBMA, OR
(stratified by smoking status)
ES (95% CI)
%
Weight
Active smokers only
Yuan 2012
0.97 (0.56, 1.66)
72.65
Never smokers only
Yuan 2014
1.00 (0.41, 2.41)
27.35
Heterogeneity between groups: p = 0.954
Overall (I-squared = 0.0%, p = 0.954)
0.98 (0.62, 1.55)
100.00
.1
1
10
Lower risk with increased exposure
Higher risk with increased exposure

(**e**)

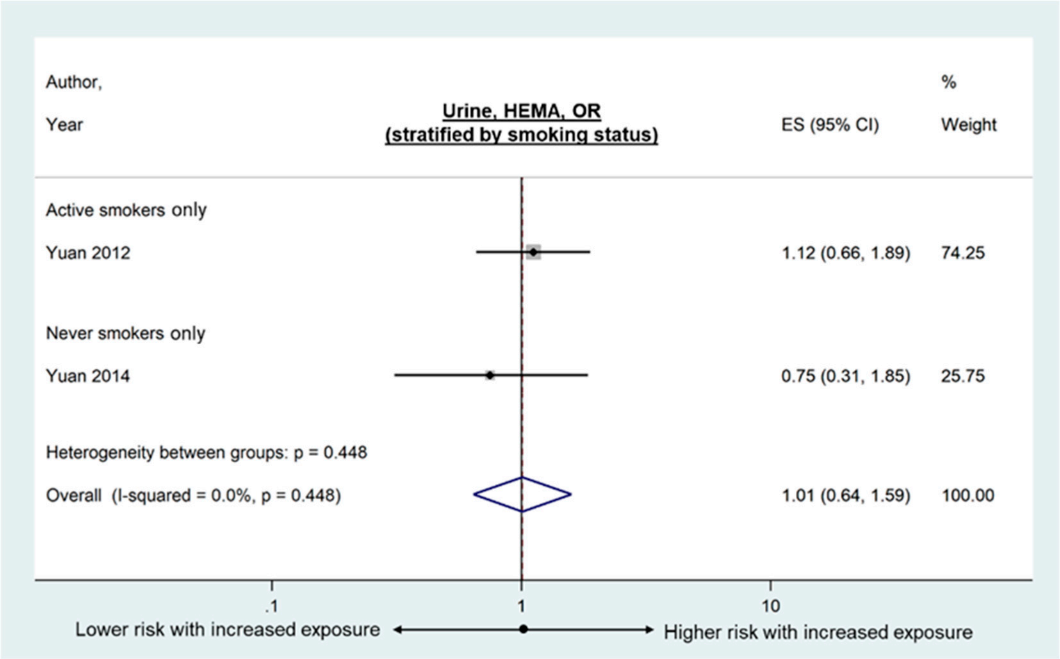
Author,
Year
Urine, HEMA, OR
(stratified by smoking status)
ES (95% CI)
%
Weight
Active smokers only
Yuan 2012
1.12 (0.66, 1.89)
74.25
Never smokers only
Yuan 2014
0.75 (0.31, 1.85)
25.75
Heterogeneity between groups: p = 0.448
Overall (I-squared = 0.0%, p = 0.448)
1.01 (0.64, 1.59)
100.00
.1
1
10
Lower risk with increased exposure
Higher risk with increased exposure

(**f**)

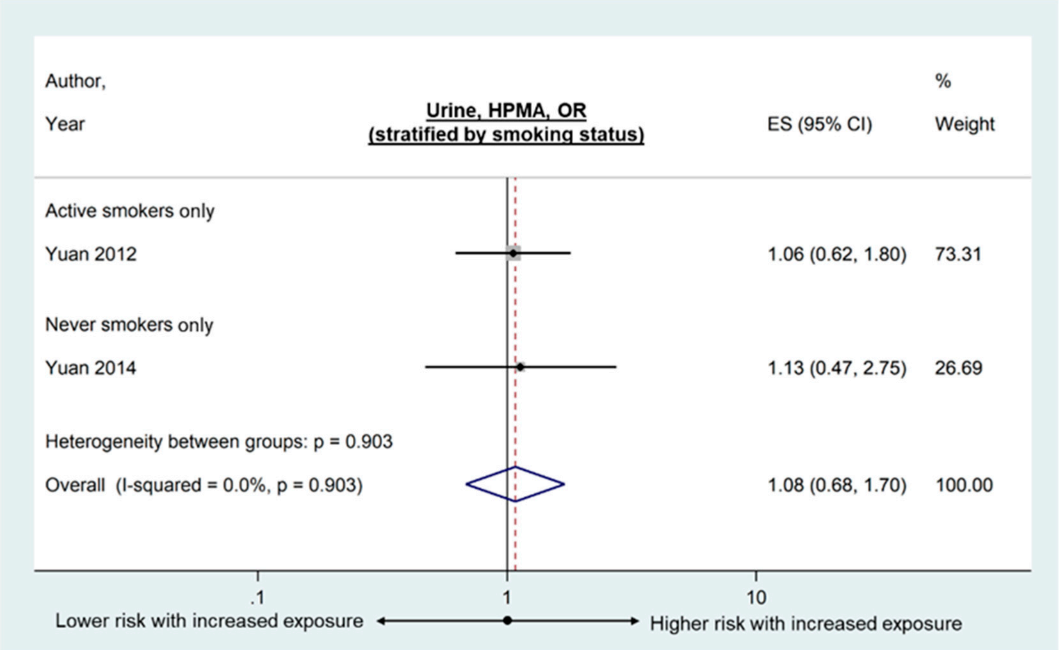

(g)

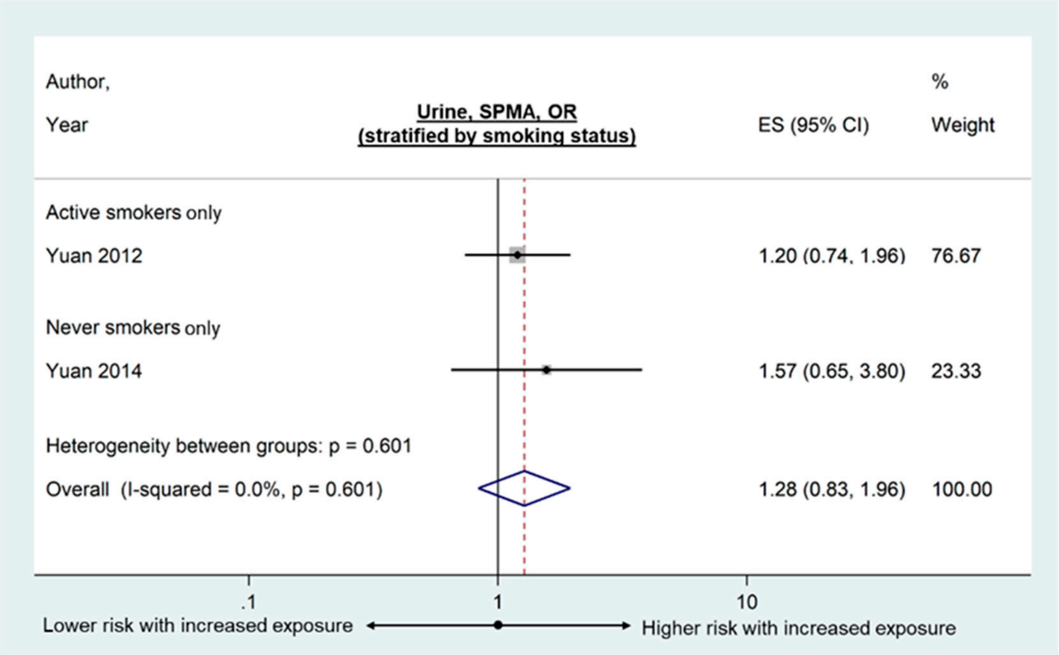

(h)

Figure S3. Effect size (ES; i.e. odds ratio (OR)) and 95% confidence interval (CI) of the association of lung cancer in relation to levels of metabolite, for metabolites that did not achieve statistical significance.

The metabolites are (a) serum/plasma pyridoxal 5'-phosphate, (b) serum/plasma methionine, (c) urinary cortisol sulfate, (d) urinary cotinine, (e) urinary HBMA, (f) urinary HEMA, (g) urinary HPMA and (h) urinary SPMA. Refer to Supplementary material List 1 for the name of abbreviated metabolites.

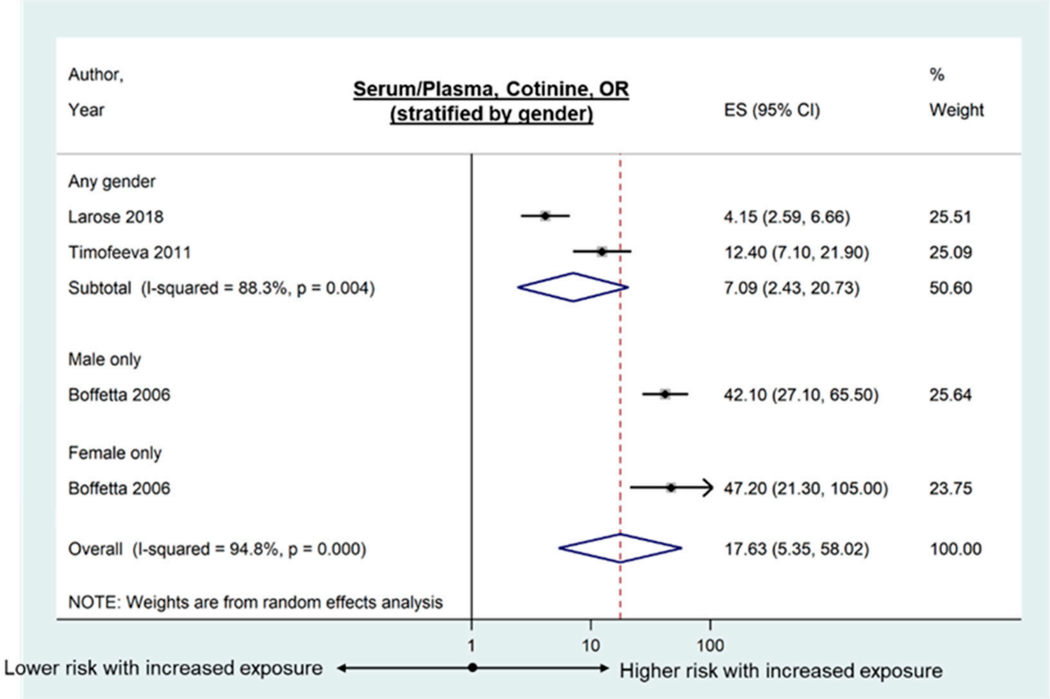
Author,
Year
Serum/Plasma, Cotinine, OR
(stratified by gender)
ES (95% CI)
%
Weight
Any gender
Larose 2018
4.15 (2.59, 6.66)
25.51
Timofeeva 2011
12.40 (7.10, 21.90)
25.09
Subtotal (I-squared = 88.3%, p = 0.004)
7.09 (2.43, 20.73)
50.60
Male only
Boffetta 2006
42.10 (27.10, 65.50)
25.64
Female only
Boffetta 2006
47.20 (21.30, 105.00)
23.75
Overall (I-squared = 94.8%, p = 0.000)
17.63 (5.35, 58.02)
100.00
NOTE: Weights are from random effects analysis
1
10
100
Lower risk with increased exposure
Higher risk with increased exposure

(**a**)

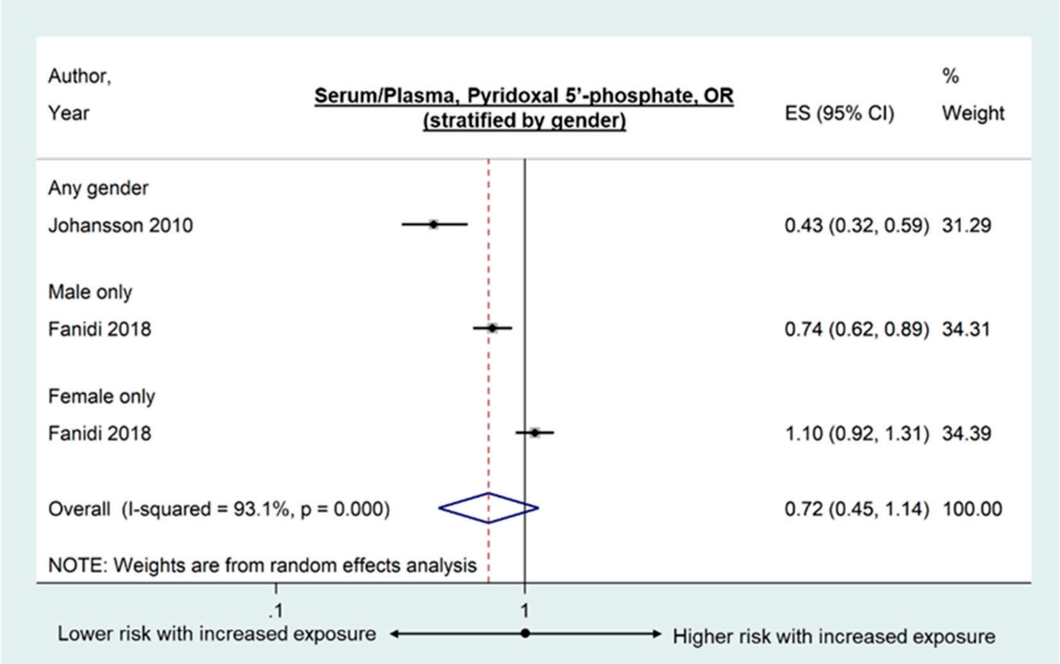
Author,
Year
Serum/Plasma, Pyridoxal 5'-phosphate, OR
(stratified by gender)
ES (95% CI)
%
Weight
Any gender
Johansson 2010
0.43 (0.32, 0.59)
31.29
Male only
Fanidi 2018
0.74 (0.62, 0.89)
34.31
Female only
Fanidi 2018
1.10 (0.92, 1.31)
34.39
Overall (I-squared = 93.1%, p = 0.000)
0.72 (0.45, 1.14)
100.00
NOTE: Weights are from random effects analysis
.1
1
Lower risk with increased exposure
Higher risk with increased exposure

(**b**)

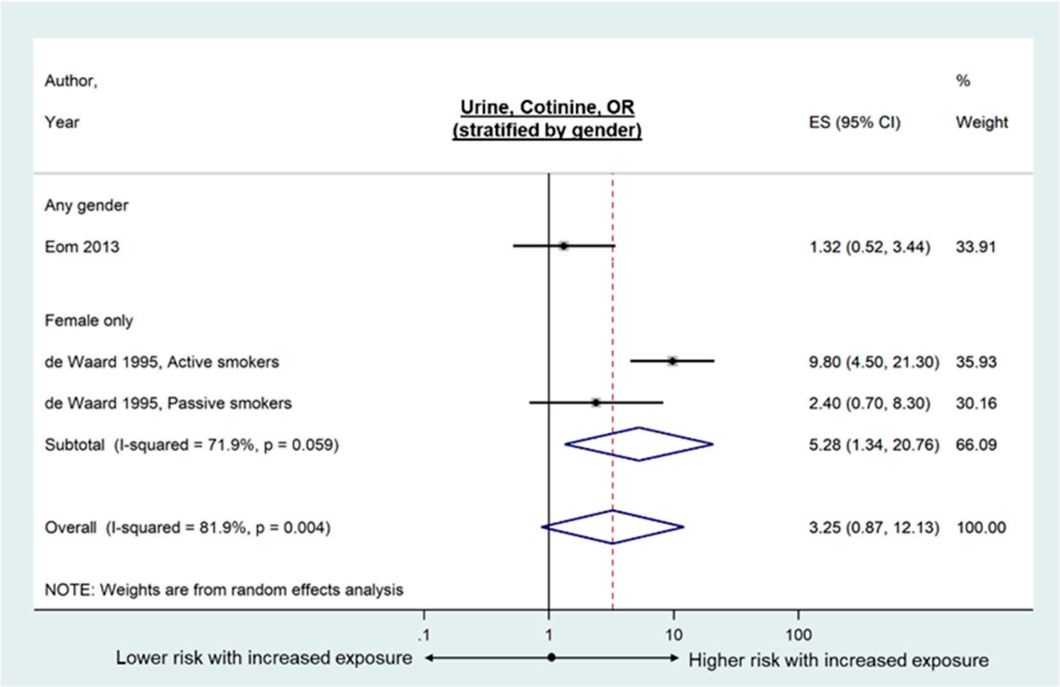
Author,
Year
Urine, Cotinine, OR
(stratified by gender)
ES (95% CI)
%
Weight
Any gender
Eom 2013
1.32 (0.52, 3.44)
33.91
Female only
de Waard 1995, Active smokers
9.80 (4.50, 21.30)
35.93
de Waard 1995, Passive smokers
2.40 (0.70, 8.30)
30.16
Subtotal (I-squared = 71.9%, p = 0.059)
5.28 (1.34, 20.76)
66.09
Overall (I-squared = 81.9%, p = 0.004)
3.25 (0.87, 12.13)
100.00
NOTE: Weights are from random effects analysis
.1
1
10
100
Lower risk with increased exposure
Higher risk with increased exposure

(**c**)

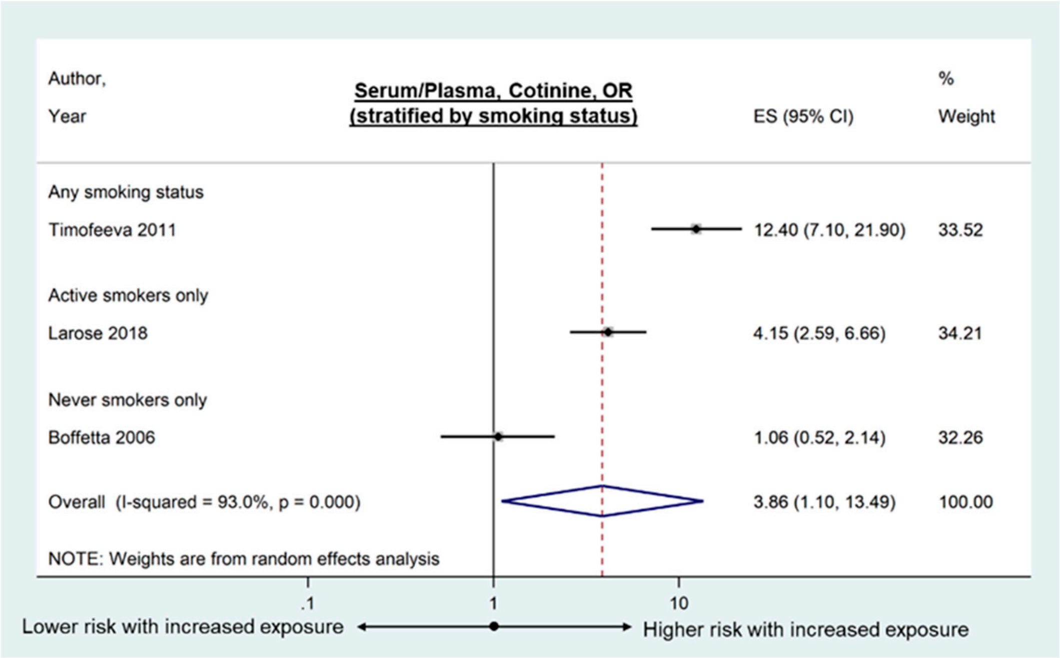
Author,
Year
Serum/Plasma, Cotinine, OR
(stratified by smoking status)
ES (95% CI)
%
Weight
Any smoking status
Timofeeva 2011
12.40 (7.10, 21.90)
33.52
Active smokers only
Larose 2018
4.15 (2.59, 6.66)
34.21
Never smokers only
Boffetta 2006
1.06 (0.52, 2.14)
32.26
Overall (I-squared = 93.0%, p = 0.000)
3.86 (1.10, 13.49)
100.00
NOTE: Weights are from random effects analysis
.1
1
10
Lower risk with increased exposure
Higher risk with increased exposure

(**d**)

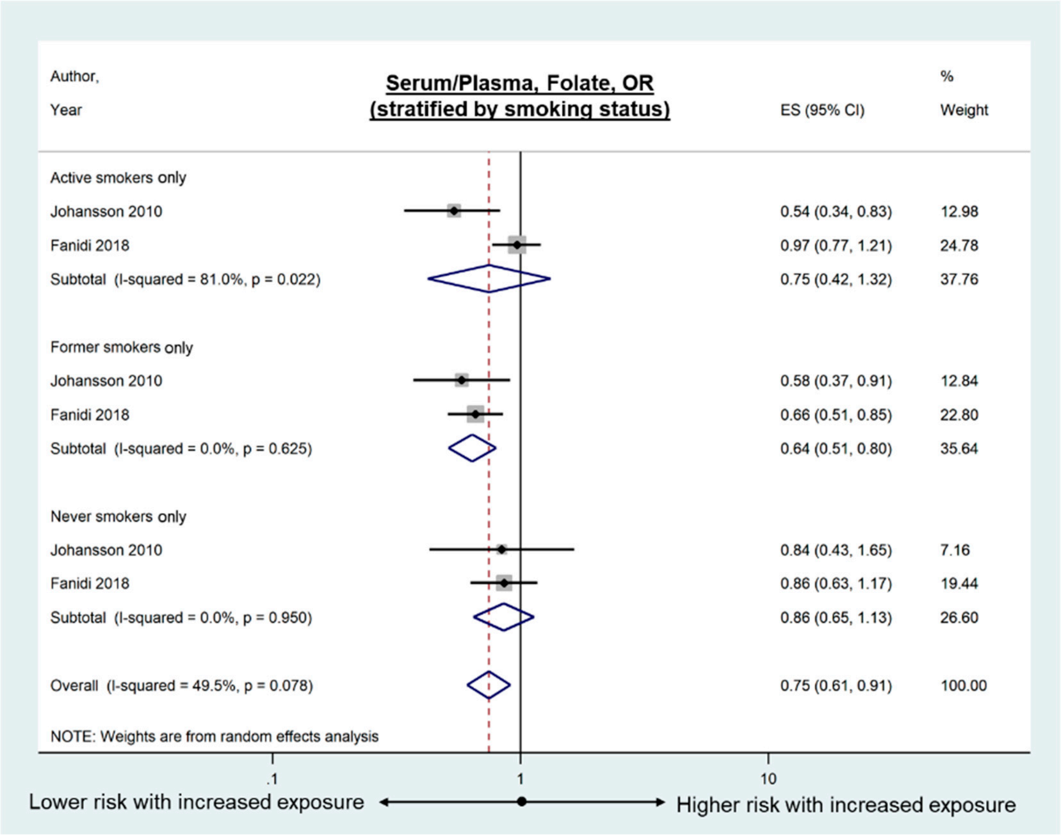
Author,
Year
Serum/Plasma, Folate, OR
(stratified by smoking status)
ES (95% CI)
%
Weight
Active smokers only
Johansson 2010
0.54 (0.34, 0.83)
12.98
Fanidi 2018
0.97 (0.77, 1.21)
24.78
Subtotal (I-squared = 81.0%, p = 0.022)
0.75 (0.42, 1.32)
37.76
Former smokers only
Johansson 2010
0.58 (0.37, 0.91)
12.84
Fanidi 2018
0.66 (0.51, 0.85)
22.80
Subtotal (I-squared = 0.0%, p = 0.625)
0.64 (0.51, 0.80)
35.64
Never smokers only
Johansson 2010
0.84 (0.43, 1.65)
7.16
Fanidi 2018
0.86 (0.63, 1.17)
19.44
Subtotal (I-squared = 0.0%, p = 0.950)
0.86 (0.65, 1.13)
26.60
Overall (I-squared = 49.5%, p = 0.078)
0.75 (0.61, 0.91)
100.00
NOTE: Weights are from random effects analysis
.1
1
10
Lower risk with increased exposure
Higher risk with increased exposure

(**e**)

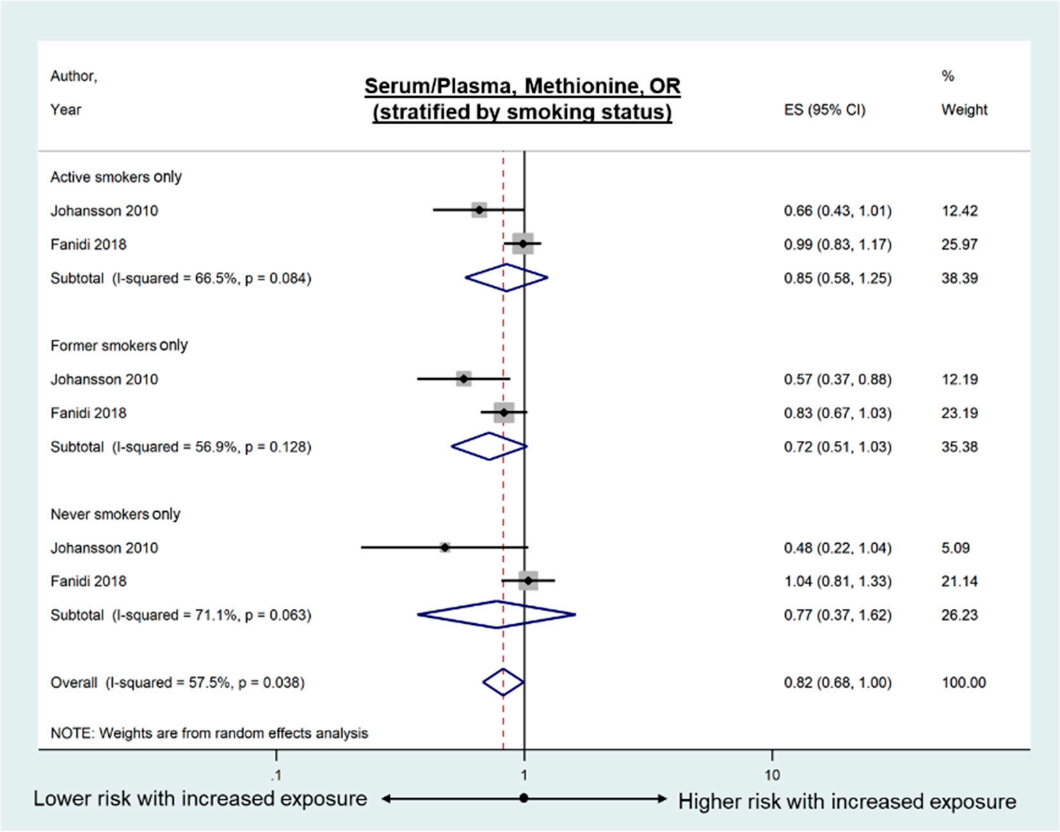
Author,
Year
Serum/Plasma, Methionine, OR
(stratified by smoking status)
ES (95% CI)
%
Weight
Active smokers only
Johansson 2010
0.66 (0.43, 1.01)
12.42
Fanidi 2018
0.99 (0.83, 1.17)
25.97
Subtotal (I-squared = 66.5%, p = 0.084)
0.85 (0.58, 1.25)
38.39
Former smokers only
Johansson 2010
0.57 (0.37, 0.88)
12.19
Fanidi 2018
0.83 (0.67, 1.03)
23.19
Subtotal (I-squared = 56.9%, p = 0.128)
0.72 (0.51, 1.03)
35.38
Never smokers only
Johansson 2010
0.48 (0.22, 1.04)
5.09
Fanidi 2018
1.04 (0.81, 1.33)
21.14
Subtotal (I-squared = 71.1%, p = 0.063)
0.77 (0.37, 1.62)
26.23
Overall (I-squared = 57.5%, p = 0.038)
0.82 (0.68, 1.00)
100.00
NOTE: Weights are from random effects analysis
.1
1
10
Lower risk with increased exposure
Higher risk with increased exposure

(**f**)

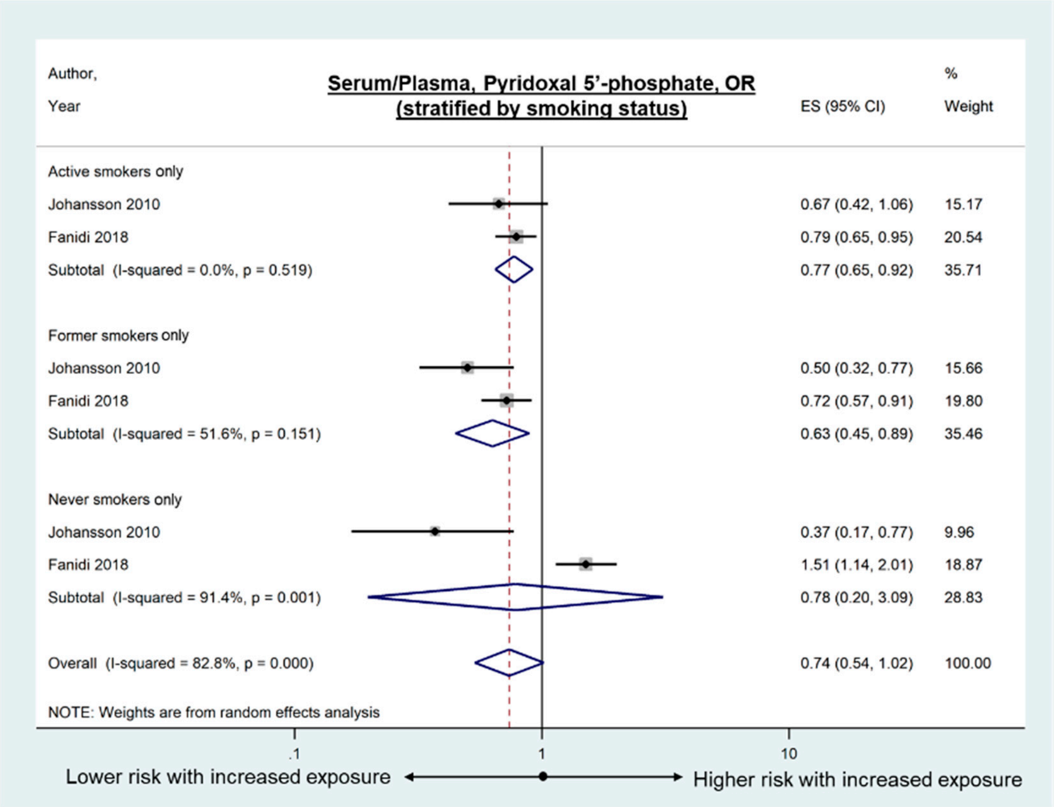

(g)

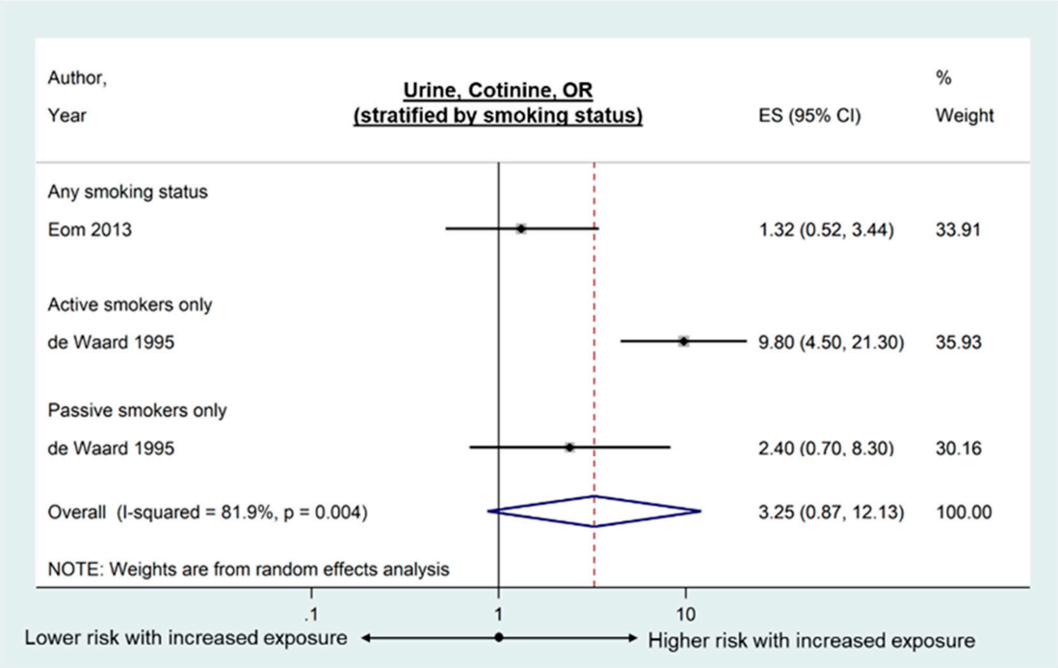

(h)

Figure S4. Effect size (ES; i.e. odds ratio (OR)) and 95% confidence interval (CI) of the association of lung cancer in relation to levels of metabolite, stratified by gender (for **a**-**c**) and smoking status (for **d**-**h**).

The metabolites are (**a**) serum/plasma cotinine, (**b**) serum/plasma pyridoxal 5'-phosphate and (**c**) urinary cotinine, stratified by gender; and (**d**) serum/plasma cotinine, (**e**) serum/plasma folate, (**f**) serum/plasma methionine, (**g**) serum/plasma pyridoxal 5'-phosphate and (**h**) urinary cotinine, stratified by smoking status.

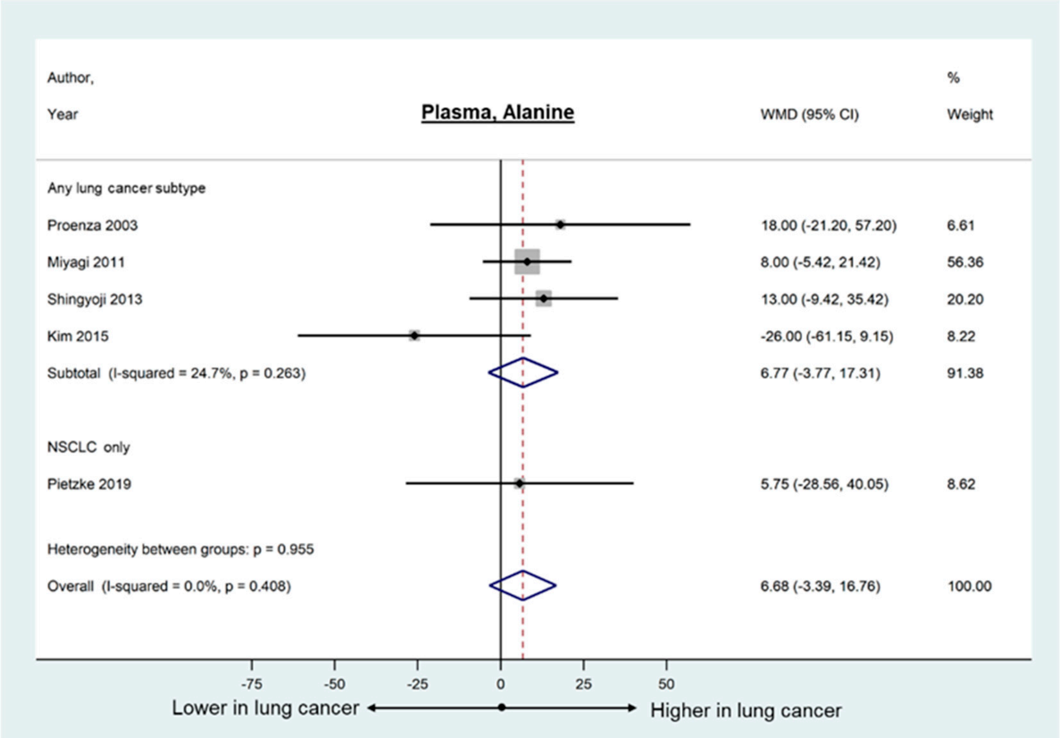
Author,
Year
Plasma, Alanine
WMD (95% CI)
%
Weight
Any lung cancer subtype
Proenza 2003 18.00 (-21.20, 57.20) 6.61
Miyagi 2011 8.00 (-5.42, 21.42) 56.36
Shingyoji 2013 13.00 (-9.42, 35.42) 20.20
Kim 2015 -26.00 (-61.15, 9.15) 8.22
Subtotal (I-squared = 24.7%, p = 0.263) 6.77 (-3.77, 17.31) 91.38
NSCLC only
Pietzke 2019 5.75 (-28.56, 40.05) 8.62
Heterogeneity between groups: p = 0.955
Overall (I-squared = 0.0%, p = 0.408) 6.68 (-3.39, 16.76) 100.00
-75 -50 -25 0 25 50
Lower in lung cancer
Higher in lung cancer

(**i**)(**a**)

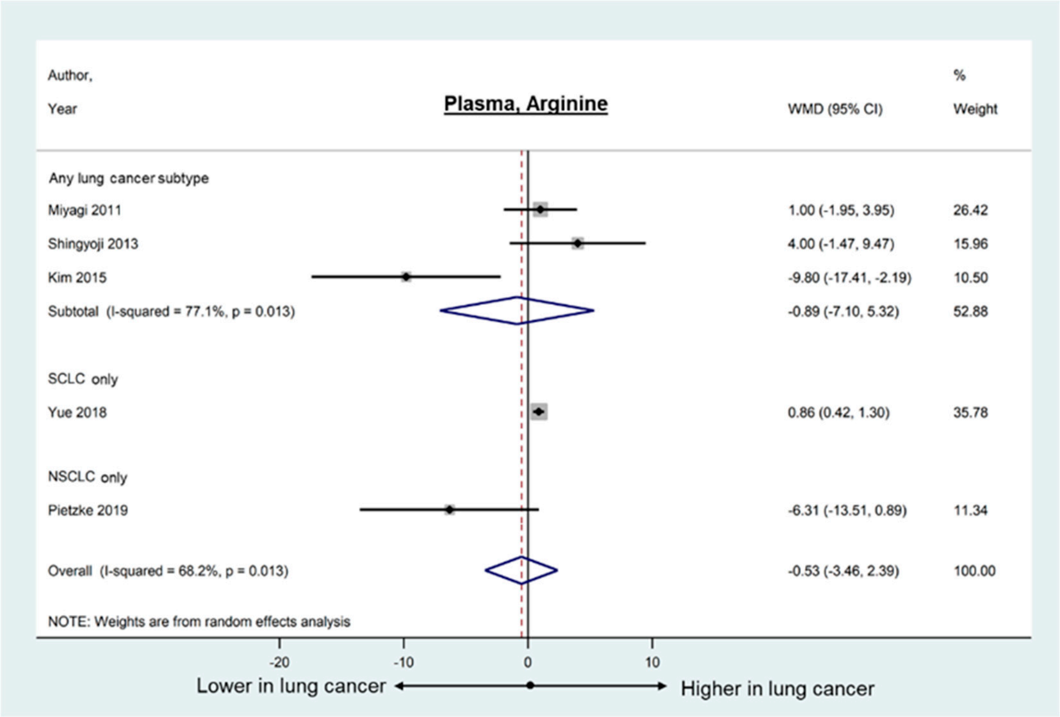
Author,
Year
Plasma, Arginine
WMD (95% CI)
%
Weight
Any lung cancer subtype
Miyagi 2011 1.00 (-1.95, 3.95) 26.42
Shingyoji 2013 4.00 (-1.47, 9.47) 15.96
Kim 2015 -9.80 (-17.41, -2.19) 10.50
Subtotal (I-squared = 77.1%, p = 0.013) -0.89 (-7.10, 5.32) 52.88
SCLC only
Yue 2018 0.86 (0.42, 1.30) 35.78
NSCLC only
Pietzke 2019 -6.31 (-13.51, 0.89) 11.34
Overall (I-squared = 68.2%, p = 0.013) -0.53 (-3.46, 2.39) 100.00
NOTE: Weights are from random effects analysis
-20 -10 0 10
Lower in lung cancer
Higher in lung cancer

(**i**)(**b**)

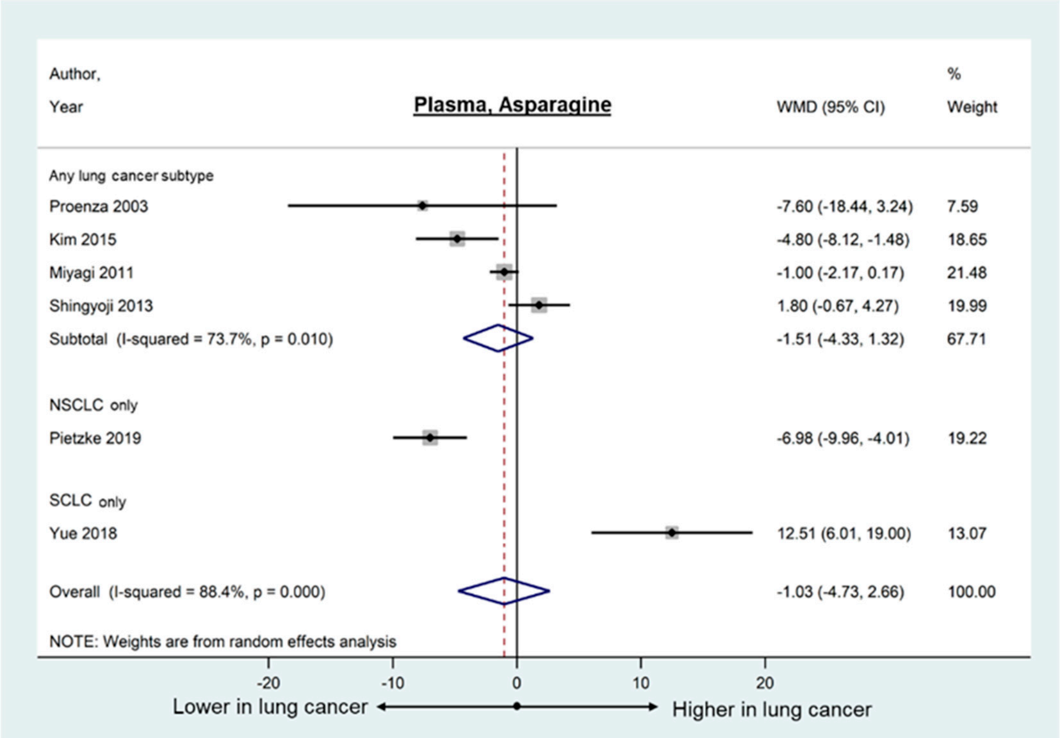
Author,
Year
Plasma, Asparagine
WMD (95% CI)
%
Weight
Any lung cancer subtype
Proenza 2003
-7.60 (-18.44, 3.24)
7.59
Kim 2015
-4.80 (-8.12, -1.48)
18.65
Miyagi 2011
-1.00 (-2.17, 0.17)
21.48
Shingyoji 2013
1.80 (-0.67, 4.27)
19.99
Subtotal (I-squared = 73.7%, p = 0.010)
-1.51 (-4.33, 1.32)
67.71
NSCLC only
Pietzke 2019
-6.98 (-9.96, -4.01)
19.22
SCLC only
Yue 2018
12.51 (6.01, 19.00)
13.07
Overall (I-squared = 88.4%, p = 0.000)
-1.03 (-4.73, 2.66)
100.00
NOTE: Weights are from random effects analysis
-20
-10
0
10
20
Lower in lung cancer
Higher in lung cancer

(**i**)(**c**)

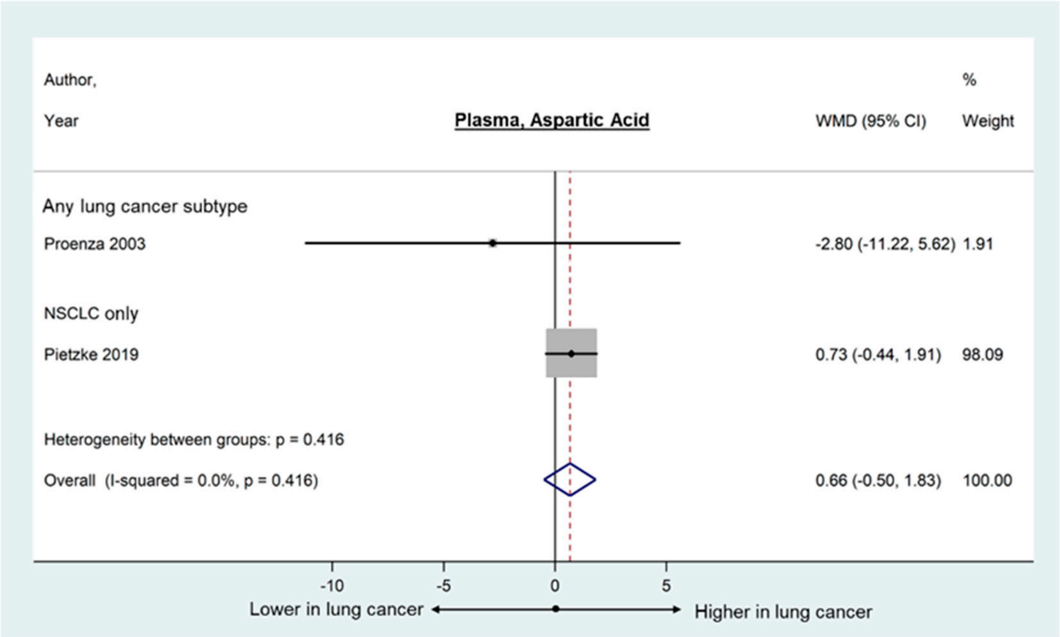
Author,
Year
Plasma, Aspartic Acid
WMD (95% CI)
%
Weight
Any lung cancer subtype
Proenza 2003
-2.80 (-11.22, 5.62)
1.91
NSCLC only
Pietzke 2019
0.73 (-0.44, 1.91)
98.09
Heterogeneity between groups: p = 0.416
Overall (I-squared = 0.0%, p = 0.416)
0.66 (-0.50, 1.83)
100.00
-10
-5
0
5
Lower in lung cancer
Higher in lung cancer

(**i**)(**d**)

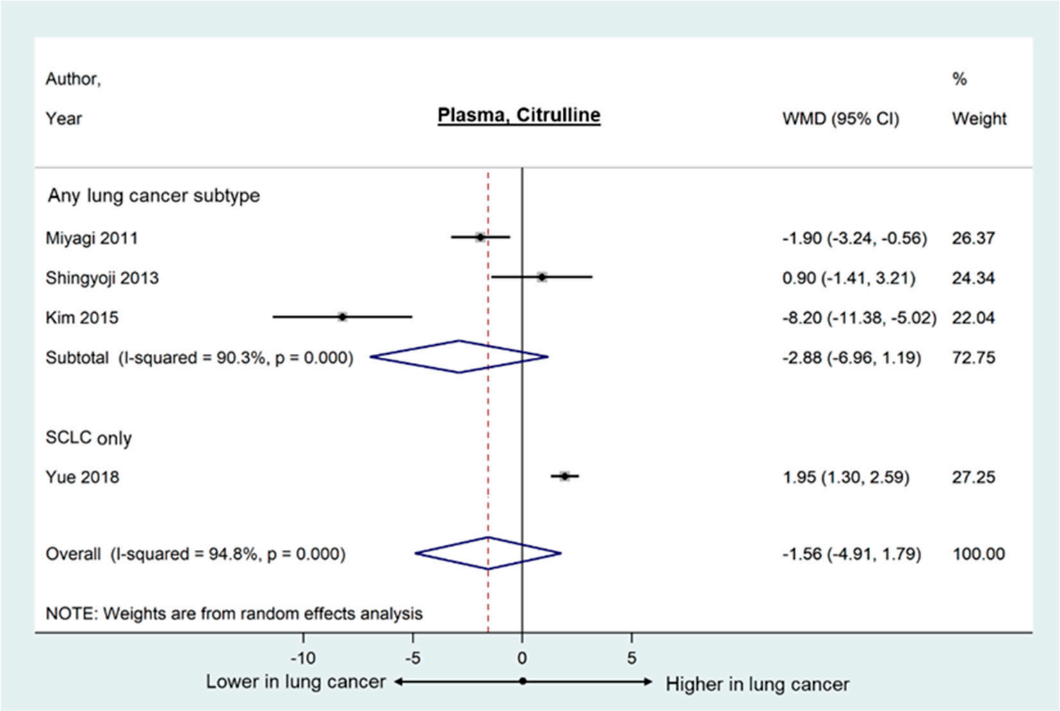
Author,
Year
Plasma, Citrulline
WMD (95% CI)
%
Weight
Any lung cancer subtype
Miyagi 2011 -1.90 (-3.24, -0.56) 26.37
Shingyoji 2013 0.90 (-1.41, 3.21) 24.34
Kim 2015 -8.20 (-11.38, -5.02) 22.04
Subtotal (I-squared = 90.3%, p = 0.000) -2.88 (-6.96, 1.19) 72.75
SCLC only
Yue 2018 1.95 (1.30, 2.59) 27.25
Overall (I-squared = 94.8%, p = 0.000) -1.56 (-4.91, 1.79) 100.00
NOTE: Weights are from random effects analysis
-10
-5
0
5
Lower in lung cancer
Higher in lung cancer

(**i**)(**e**)

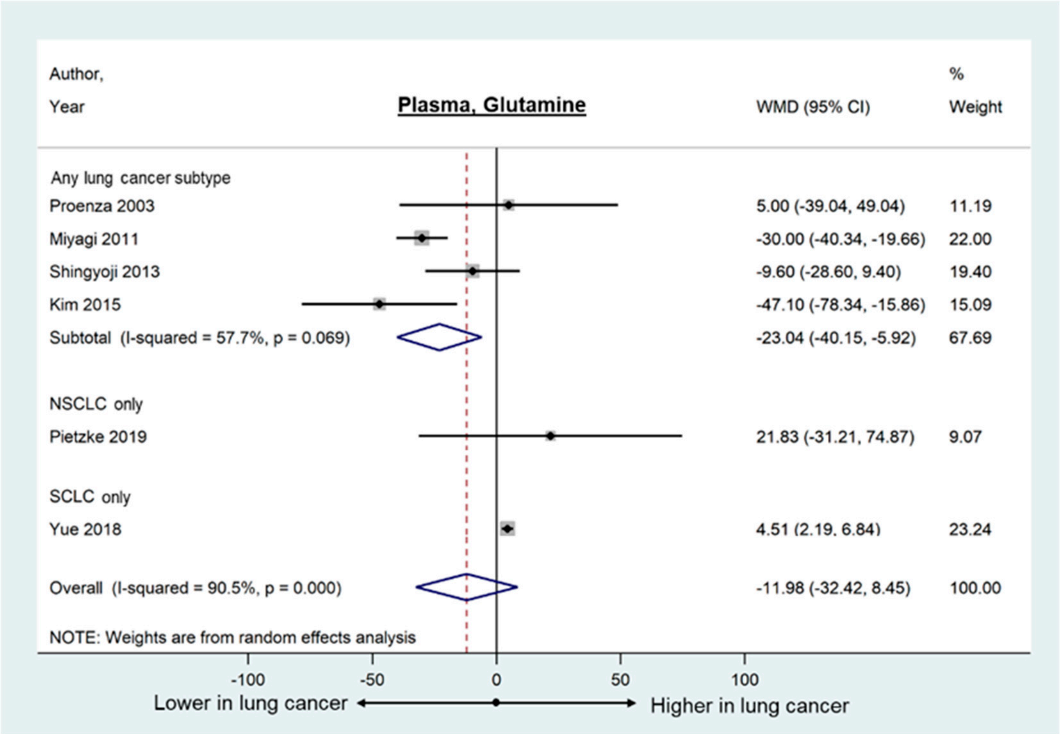
Author,
Year
Plasma, Glutamine
WMD (95% CI)
%
Weight
Any lung cancer subtype
Proenza 2003 5.00 (-39.04, 49.04) 11.19
Miyagi 2011 -30.00 (-40.34, -19.66) 22.00
Shingyoji 2013 -9.60 (-28.60, 9.40) 19.40
Kim 2015 -47.10 (-78.34, -15.86) 15.09
Subtotal (I-squared = 57.7%, p = 0.069) -23.04 (-40.15, -5.92) 67.69
NSCLC only
Pietzke 2019 21.83 (-31.21, 74.87) 9.07
SCLC only
Yue 2018 4.51 (2.19, 6.84) 23.24
Overall (I-squared = 90.5%, p = 0.000) -11.98 (-32.42, 8.45) 100.00
NOTE: Weights are from random effects analysis
-100
-50
0
50
100
Lower in lung cancer
Higher in lung cancer

(**i**)(**f**)

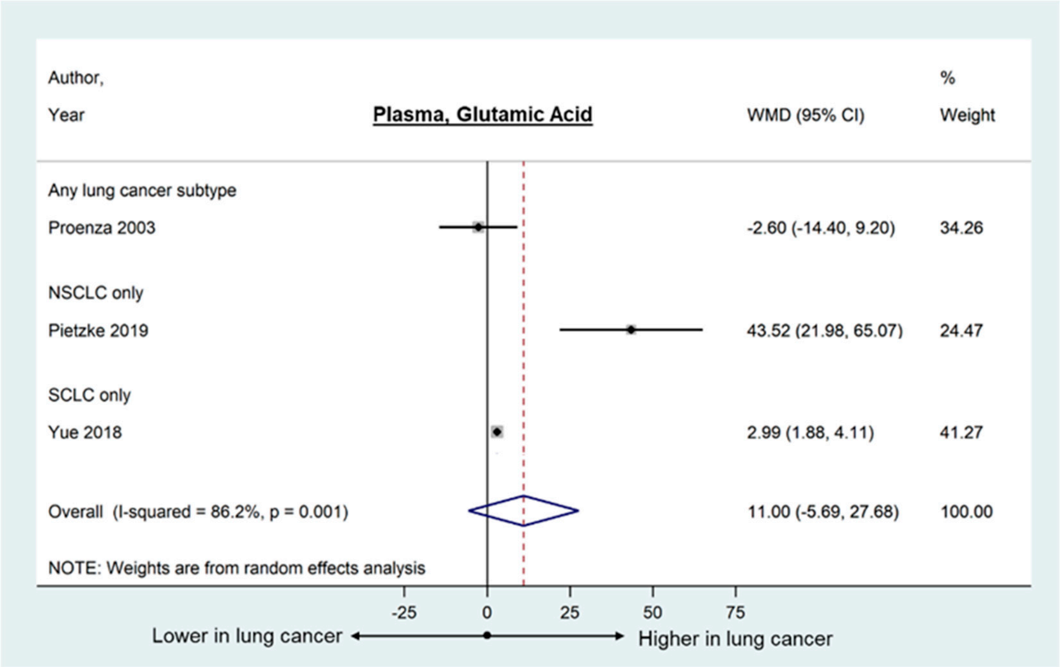
Author,
Year
Plasma, Glutamic Acid
WMD (95% CI)
%
Weight
Any lung cancer subtype
Proenza 2003
-2.60 (-14.40, 9.20)
34.26
NSCLC only
Pietzke 2019
43.52 (21.98, 65.07)
24.47
SCLC only
Yue 2018
2.99 (1.88, 4.11)
41.27
Overall (I-squared = 86.2%, p = 0.001)
11.00 (-5.69, 27.68)
100.00
NOTE: Weights are from random effects analysis
-25
0
25
50
75
Lower in lung cancer
Higher in lung cancer

(i)(g)

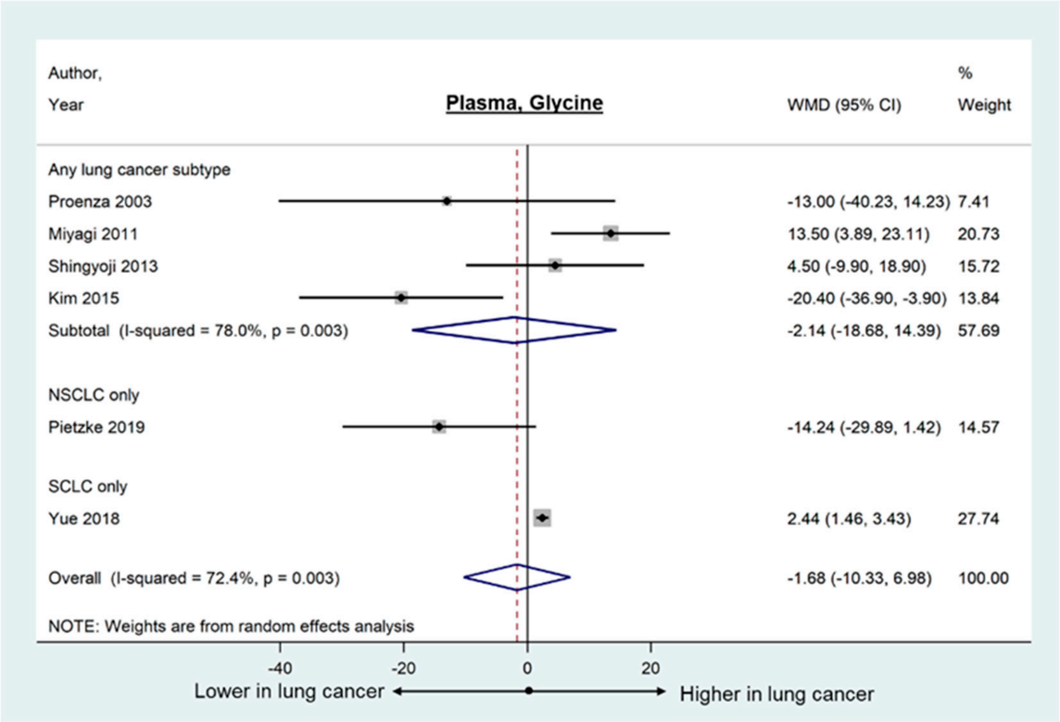
Author,
Year
Plasma, Glycine
WMD (95% CI)
%
Weight
Any lung cancer subtype
Proenza 2003
-13.00 (-40.23, 14.23) 7.41
Miyagi 2011
13.50 (3.89, 23.11)
20.73
Shingyoji 2013
4.50 (-9.90, 18.90)
15.72
Kim 2015
-20.40 (-36.90, -3.90)
13.84
Subtotal (I-squared = 78.0%, p = 0.003)
-2.14 (-18.68, 14.39)
57.69
NSCLC only
Pietzke 2019
-14.24 (-29.89, 1.42)
14.57
SCLC only
Yue 2018
2.44 (1.46, 3.43)
27.74
Overall (I-squared = 72.4%, p = 0.003)
-1.68 (-10.33, 6.98)
100.00
NOTE: Weights are from random effects analysis
-40
-20
0
20
Lower in lung cancer
Higher in lung cancer

(i)(h)

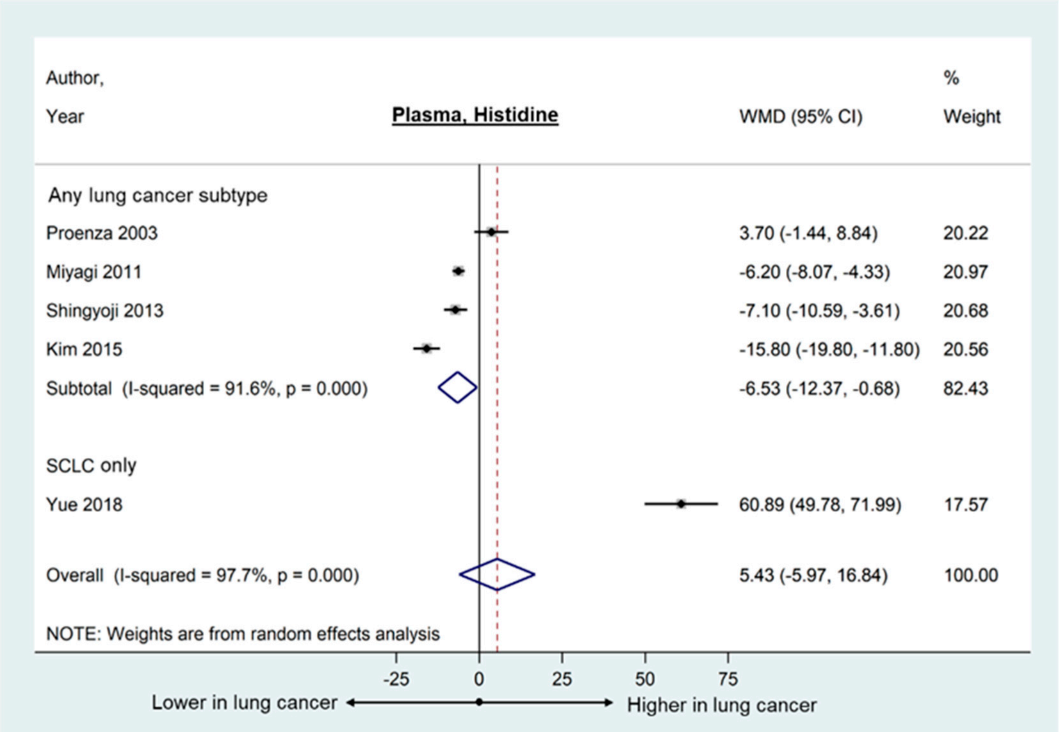
Author,
Year
%
Weight
Plasma, Histidine
WMD (95% CI)
Any lung cancer subtype
Proenza 2003
3.70 (-1.44, 8.84)
20.22
Miyagi 2011
-6.20 (-8.07, -4.33)
20.97
Shingyoji 2013
-7.10 (-10.59, -3.61)
20.68
Kim 2015
-15.80 (-19.80, -11.80)
20.56
Subtotal (I-squared = 91.6%, p = 0.000)
-6.53 (-12.37, -0.68)
82.43
SCLC only
Yue 2018
60.89 (49.78, 71.99)
17.57
Overall (I-squared = 97.7%, p = 0.000)
5.43 (-5.97, 16.84)
100.00
NOTE: Weights are from random effects analysis
-25
0
25
50
75
Lower in lung cancer
Higher in lung cancer

(i)(i)

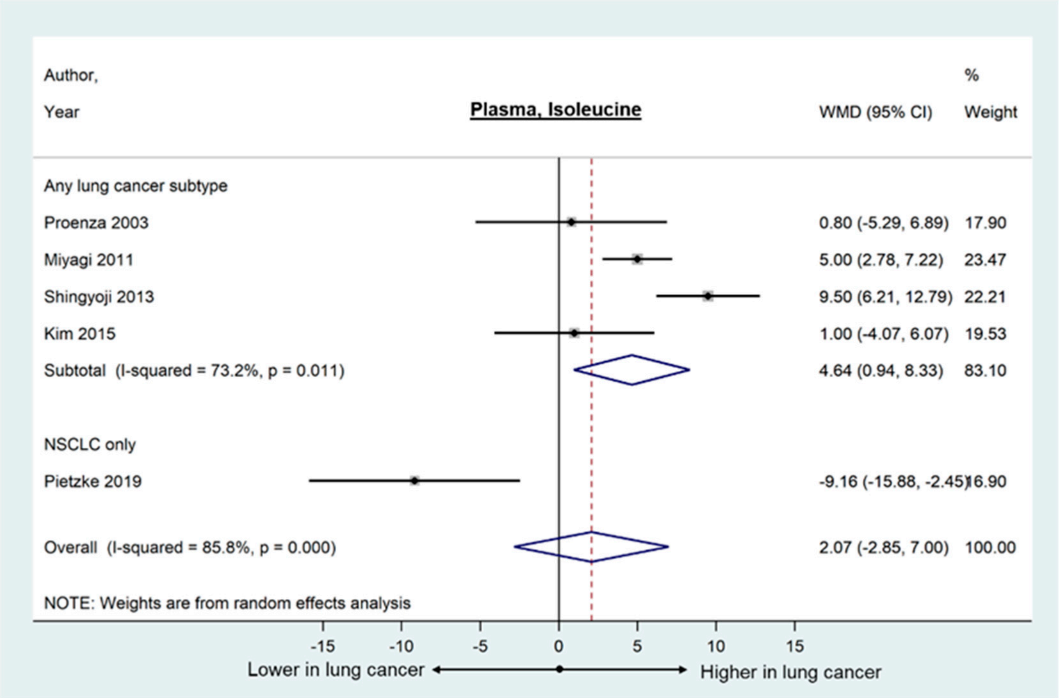
Author,
Year
%
Weight
Plasma, Isoleucine
WMD (95% CI)
Any lung cancer subtype
Proenza 2003
0.80 (-5.29, 6.89)
17.90
Miyagi 2011
5.00 (2.78, 7.22)
23.47
Shingyoji 2013
9.50 (6.21, 12.79)
22.21
Kim 2015
1.00 (-4.07, 6.07)
19.53
Subtotal (I-squared = 73.2%, p = 0.011)
4.64 (0.94, 8.33)
83.10
NSCLC only
Pietzke 2019
-9.16 (-15.88, -2.45)16.90
Overall (I-squared = 85.8%, p = 0.000)
2.07 (-2.85, 7.00)
100.00
NOTE: Weights are from random effects analysis
-15
-10
-5
0
5
10
15
Lower in lung cancer
Higher in lung cancer

(i)(j)

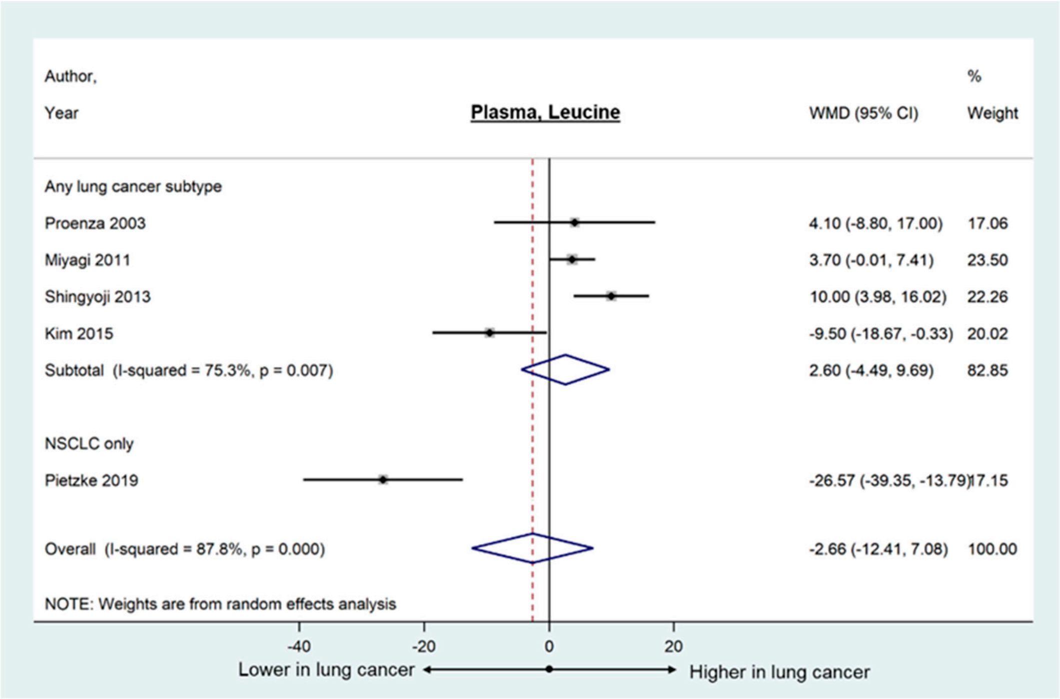
Author,
Year
Plasma, Leucine
WMD (95% CI)
%
Weight
Any lung cancer subtype
Proenza 2003
4.10 (-8.80, 17.00)
17.06
Miyagi 2011
3.70 (-0.01, 7.41)
23.50
Shingyoji 2013
10.00 (3.98, 16.02)
22.26
Kim 2015
-9.50 (-18.67, -0.33)
20.02
Subtotal (I-squared = 75.3%, p = 0.007)
2.60 (-4.49, 9.69)
82.85
NSCLC only
Pietzke 2019
-26.57 (-39.35, -13.79)
17.15
Overall (I-squared = 87.8%, p = 0.000)
-2.66 (-12.41, 7.08)
100.00
NOTE: Weights are from random effects analysis
-40
-20
0
20
Lower in lung cancer
Higher in lung cancer

(**i**)(**k**)

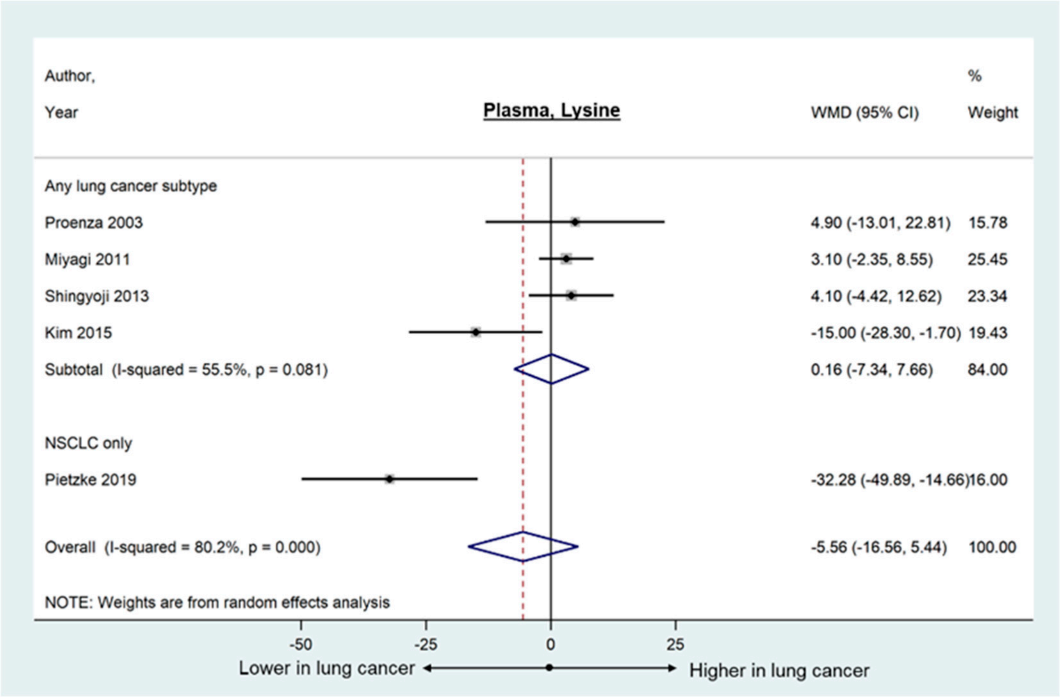
Author,
Year
Plasma, Lysine
WMD (95% CI)
%
Weight
Any lung cancer subtype
Proenza 2003
4.90 (-13.01, 22.81)
15.78
Miyagi 2011
3.10 (-2.35, 8.55)
25.45
Shingyoji 2013
4.10 (-4.42, 12.62)
23.34
Kim 2015
-15.00 (-28.30, -1.70)
19.43
Subtotal (I-squared = 55.5%, p = 0.081)
0.16 (-7.34, 7.66)
84.00
NSCLC only
Pietzke 2019
-32.28 (-49.89, -14.66)
16.00
Overall (I-squared = 80.2%, p = 0.000)
-5.56 (-16.56, 5.44)
100.00
NOTE: Weights are from random effects analysis
-50
-25
0
25
Lower in lung cancer
Higher in lung cancer

(**i**)(**l**)

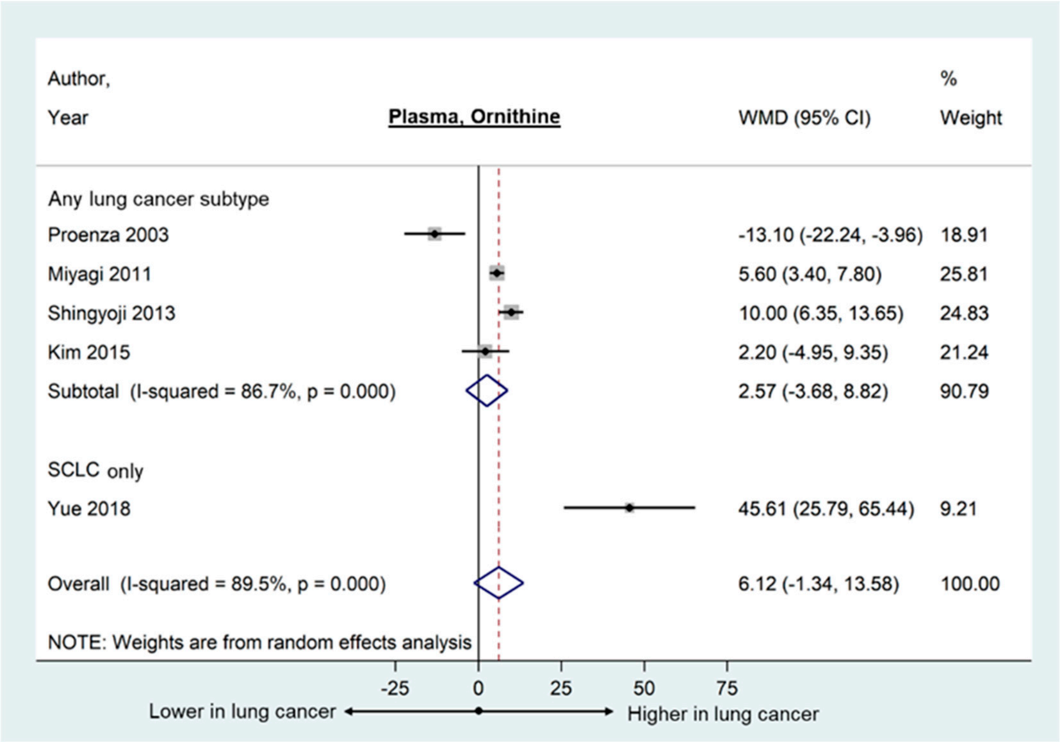
Author,
Year
Plasma, Ornithine
WMD (95% CI)
%
Weight
Any lung cancer subtype
Proenza 2003
-13.10 (-22.24, -3.96)
18.91
Miyagi 2011
5.60 (3.40, 7.80)
25.81
Shingyoji 2013
10.00 (6.35, 13.65)
24.83
Kim 2015
2.20 (-4.95, 9.35)
21.24
Subtotal (I-squared = 86.7%, p = 0.000)
2.57 (-3.68, 8.82)
90.79
SCLC only
Yue 2018
45.61 (25.79, 65.44)
9.21
Overall (I-squared = 89.5%, p = 0.000)
6.12 (-1.34, 13.58)
100.00
NOTE: Weights are from random effects analysis
-25
0
25
50
75
Lower in lung cancer
Higher in lung cancer

(i)(m)

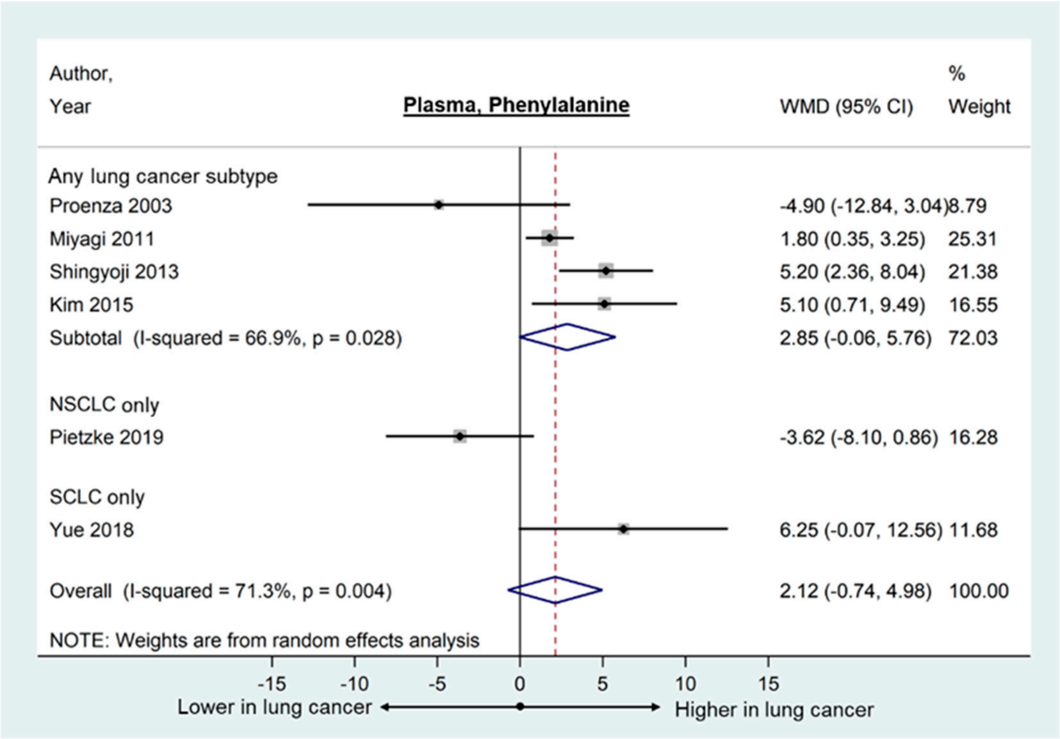
Author,
Year
Plasma, Phenylalanine
WMD (95% CI)
%
Weight
Any lung cancer subtype
Proenza 2003
-4.90 (-12.84, 3.04)8.79
Miyagi 2011
1.80 (0.35, 3.25)
25.31
Shingyoji 2013
5.20 (2.36, 8.04)
21.38
Kim 2015
5.10 (0.71, 9.49)
16.55
Subtotal (I-squared = 66.9%, p = 0.028)
2.85 (-0.06, 5.76)
72.03
NSCLC only
Pietzke 2019
-3.62 (-8.10, 0.86)
16.28
SCLC only
Yue 2018
6.25 (-0.07, 12.56)
11.68
Overall (I-squared = 71.3%, p = 0.004)
2.12 (-0.74, 4.98)
100.00
NOTE: Weights are from random effects analysis
-15
-10
-5
0
5
10
15
Lower in lung cancer
Higher in lung cancer

(i)(n)

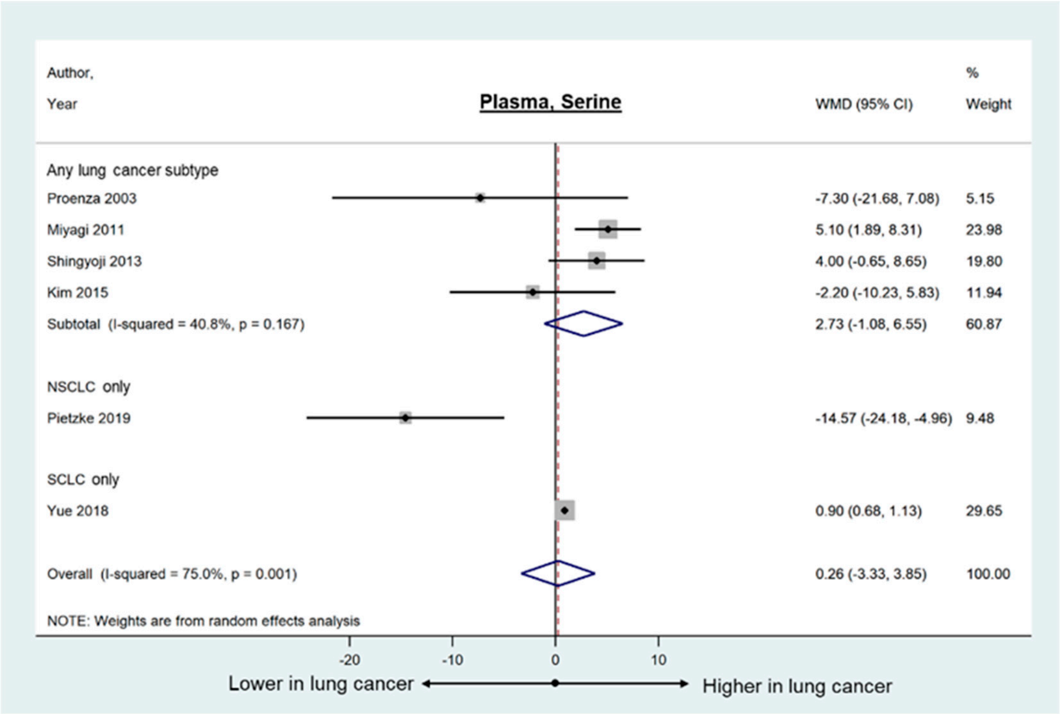
Author,
Year
Plasma, Serine
WMD (95% CI)
%
Weight
Any lung cancer subtype
Proenza 2003 -7.30 (-21.68, 7.08) 5.15
Miyagi 2011 5.10 (1.89, 8.31) 23.98
Shingyoji 2013 4.00 (-0.65, 8.65) 19.80
Kim 2015 -2.20 (-10.23, 5.83) 11.94
Subtotal (I-squared = 40.8%, p = 0.167) 2.73 (-1.08, 6.55) 60.87
NSCLC only
Pietzke 2019 -14.57 (-24.18, -4.96) 9.48
SCLC only
Yue 2018 0.90 (0.68, 1.13) 29.65
Overall (I-squared = 75.0%, p = 0.001) 0.26 (-3.33, 3.85) 100.00
NOTE: Weights are from random effects analysis
-20
-10
0
10
Lower in lung cancer
Higher in lung cancer

(**i**)(**o**)

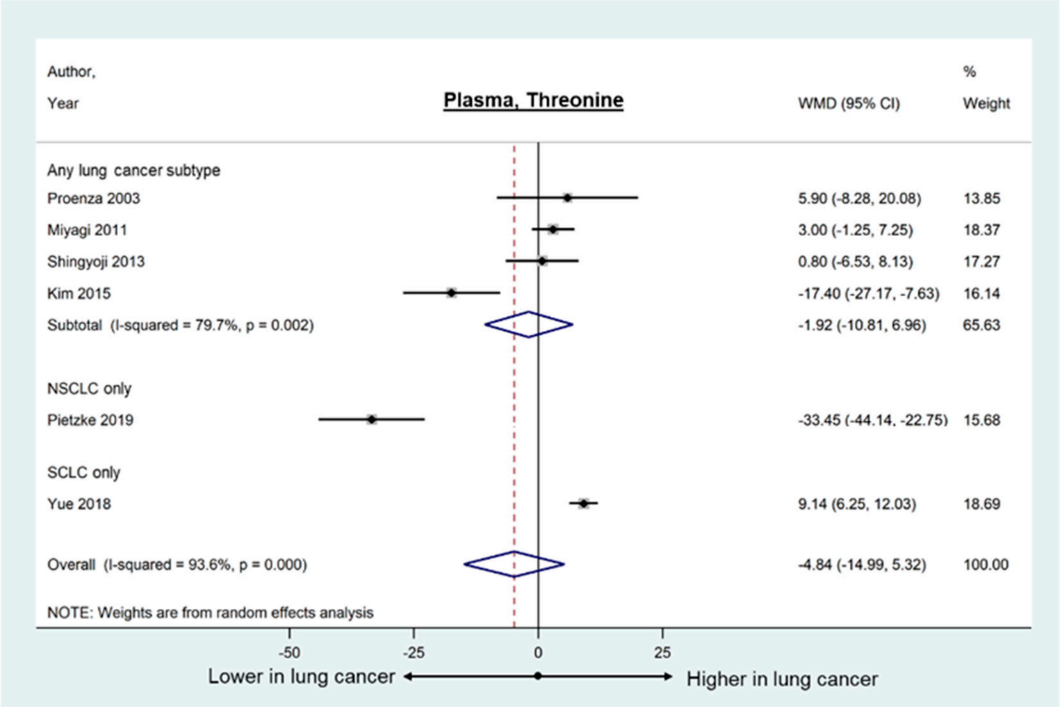
Author,
Year
Plasma, Threonine
WMD (95% CI)
%
Weight
Any lung cancer subtype
Proenza 2003 5.90 (-8.28, 20.08) 13.85
Miyagi 2011 3.00 (-1.25, 7.25) 18.37
Shingyoji 2013 0.80 (-6.53, 8.13) 17.27
Kim 2015 -17.40 (-27.17, -7.63) 16.14
Subtotal (I-squared = 79.7%, p = 0.002) -1.92 (-10.81, 6.96) 65.63
NSCLC only
Pietzke 2019 -33.45 (-44.14, -22.75) 15.68
SCLC only
Yue 2018 9.14 (6.25, 12.03) 18.69
Overall (I-squared = 93.6%, p = 0.000) -4.84 (-14.99, 5.32) 100.00
NOTE: Weights are from random effects analysis
-50
-25
0
25
Lower in lung cancer
Higher in lung cancer

(**i**)(**p**)

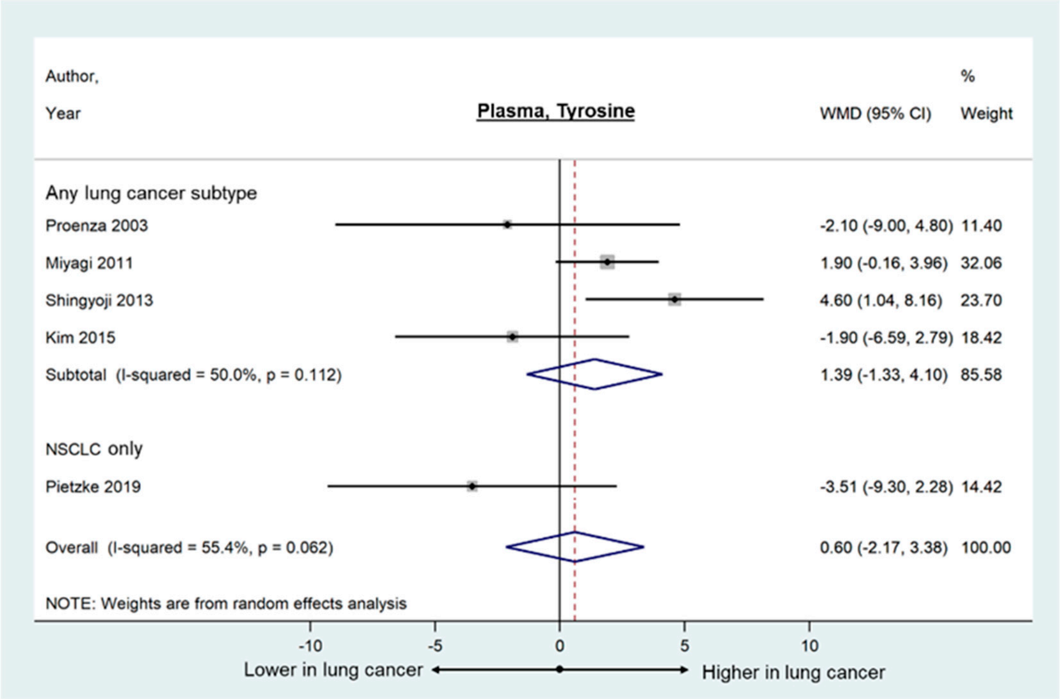
Author,
Year
Plasma, Tyrosine
WMD (95% CI)
%
Weight
Any lung cancer subtype
Proenza 2003 -2.10 (-9.00, 4.80) 11.40
Miyagi 2011 1.90 (-0.16, 3.96) 32.06
Shingyoji 2013 4.60 (1.04, 8.16) 23.70
Kim 2015 -1.90 (-6.59, 2.79) 18.42
Subtotal (I-squared = 50.0%, p = 0.112) 1.39 (-1.33, 4.10) 85.58
NSCLC only
Pietzke 2019 -3.51 (-9.30, 2.28) 14.42
Overall (I-squared = 55.4%, p = 0.062) 0.60 (-2.17, 3.38) 100.00
NOTE: Weights are from random effects analysis
-10
-5
0
5
10
Lower in lung cancer
Higher in lung cancer

(**i**)(**q**)

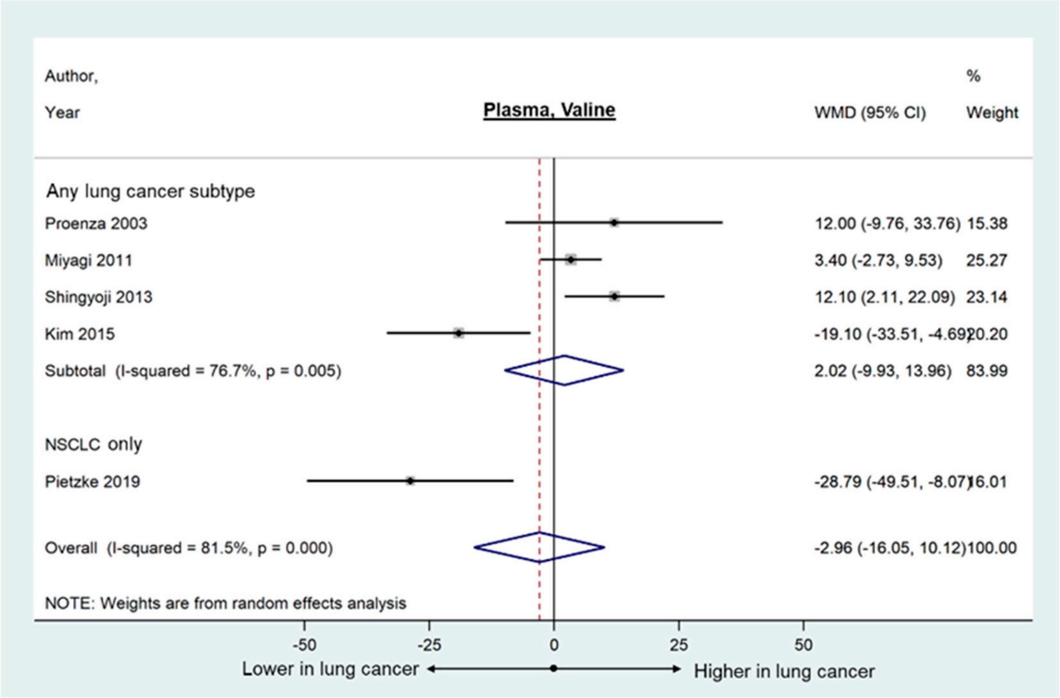
Author,
Year
Plasma, Valine
WMD (95% CI)
%
Weight
Any lung cancer subtype
Proenza 2003 12.00 (-9.76, 33.76) 15.38
Miyagi 2011 3.40 (-2.73, 9.53) 25.27
Shingyoji 2013 12.10 (2.11, 22.09) 23.14
Kim 2015 -19.10 (-33.51, -4.69) 20.20
Subtotal (I-squared = 76.7%, p = 0.005) 2.02 (-9.93, 13.96) 83.99
NSCLC only
Pietzke 2019 -28.79 (-49.51, -8.07) 16.01
Overall (I-squared = 81.5%, p = 0.000) -2.96 (-16.05, 10.12) 100.00
NOTE: Weights are from random effects analysis
-50
-25
0
25
50
Lower in lung cancer
Higher in lung cancer

(**i**)(**r**)

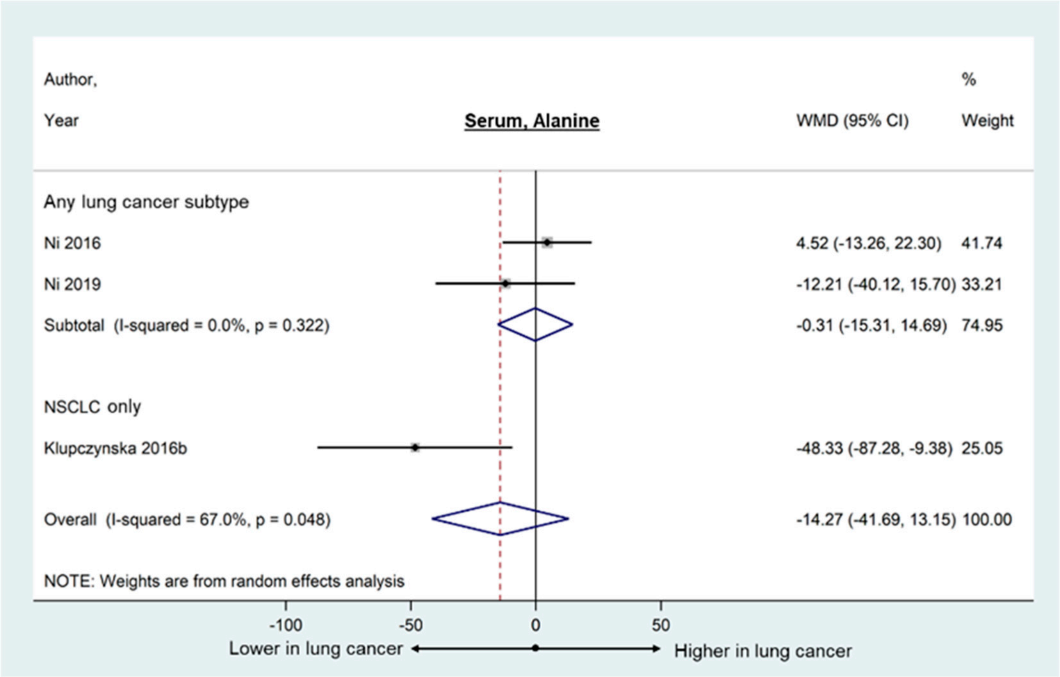
Author,
Year
Serum, Alanine
WMD (95% CI)
%
Weight
Any lung cancer subtype
Ni 2016
4.52 (-13.26, 22.30) 41.74
Ni 2019
-12.21 (-40.12, 15.70) 33.21
Subtotal (I-squared = 0.0%, p = 0.322)
-0.31 (-15.31, 14.69) 74.95
NSCLC only
Klupczynska 2016b
-48.33 (-87.28, -9.38) 25.05
Overall (I-squared = 67.0%, p = 0.048)
-14.27 (-41.69, 13.15) 100.00
NOTE: Weights are from random effects analysis
-100
-50
0
50
Lower in lung cancer
Higher in lung cancer

(ii)(a)

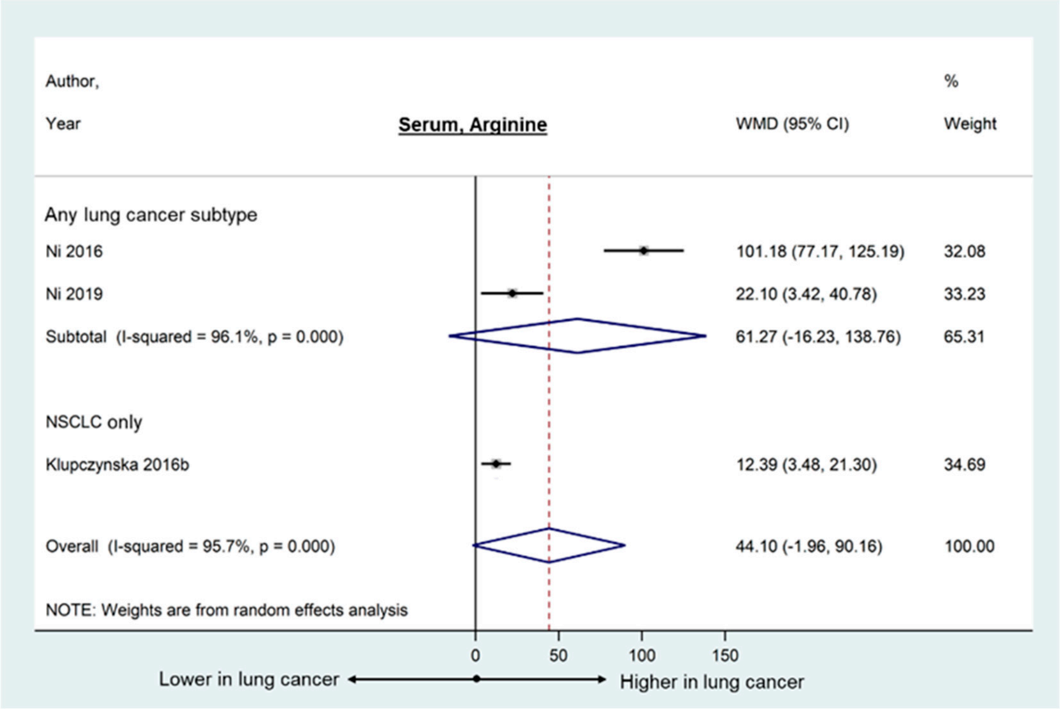
Author,
Year
Serum, Arginine
WMD (95% CI)
%
Weight
Any lung cancer subtype
Ni 2016
101.18 (77.17, 125.19) 32.08
Ni 2019
22.10 (3.42, 40.78) 33.23
Subtotal (I-squared = 96.1%, p = 0.000)
61.27 (-16.23, 138.76) 65.31
NSCLC only
Klupczynska 2016b
12.39 (3.48, 21.30) 34.69
Overall (I-squared = 95.7%, p = 0.000)
44.10 (-1.96, 90.16) 100.00
NOTE: Weights are from random effects analysis
0
50
100
150
Lower in lung cancer
Higher in lung cancer

(ii)(b)

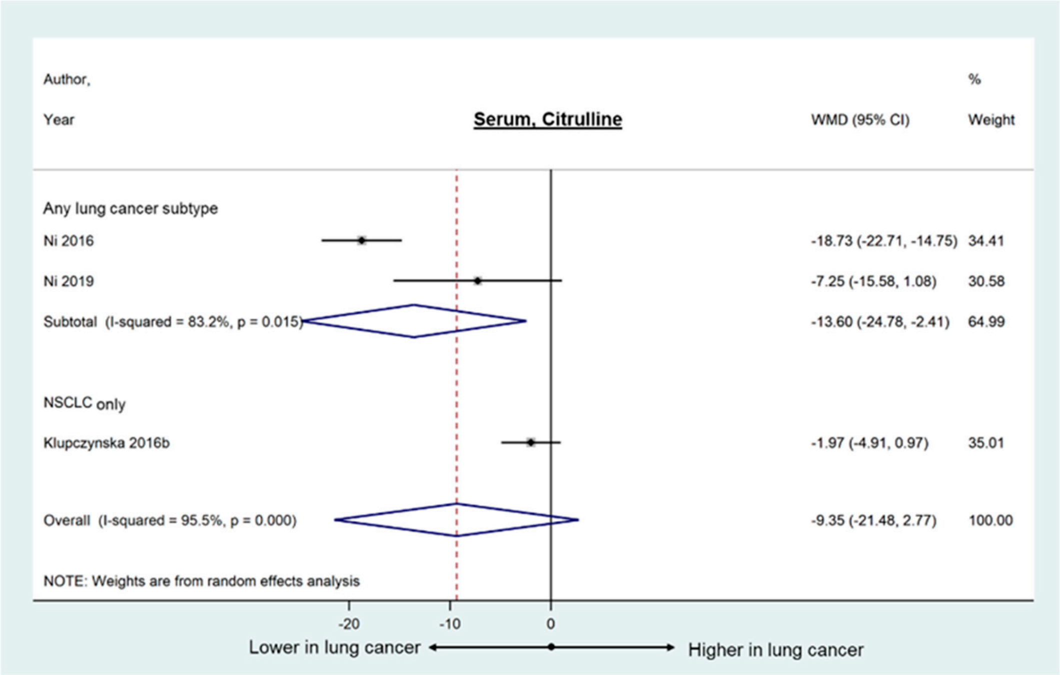
Author,
Year
Serum, Citrulline
WMD (95% CI)
%
Weight
Any lung cancer subtype
Ni 2016
-18.73 (-22.71, -14.75)
34.41
Ni 2019
-7.25 (-15.58, 1.08)
30.58
Subtotal (I-squared = 83.2%, p = 0.015)
-13.60 (-24.78, -2.41)
64.99
NSCLC only
Klupczynska 2016b
-1.97 (-4.91, 0.97)
35.01
Overall (I-squared = 95.5%, p = 0.000)
-9.35 (-21.48, 2.77)
100.00
NOTE: Weights are from random effects analysis
-20
-10
0
Lower in lung cancer
Higher in lung cancer

(ii)(c)

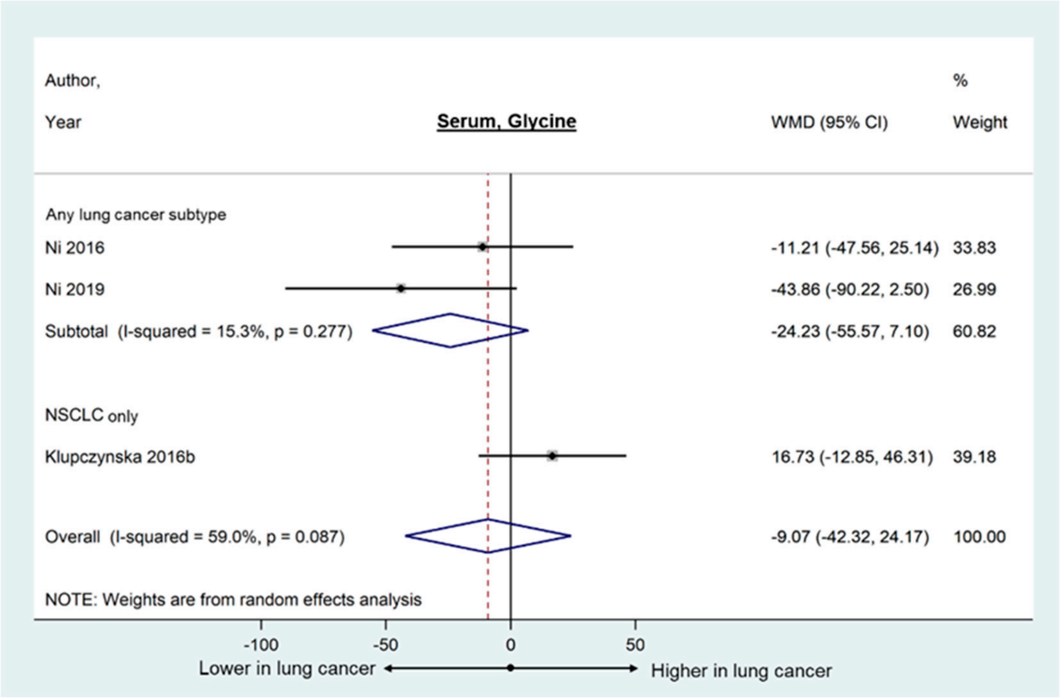
Author,
Year
Serum, Glycine
WMD (95% CI)
%
Weight
Any lung cancer subtype
Ni 2016
-11.21 (-47.56, 25.14)
33.83
Ni 2019
-43.86 (-90.22, 2.50)
26.99
Subtotal (I-squared = 15.3%, p = 0.277)
-24.23 (-55.57, 7.10)
60.82
NSCLC only
Klupczynska 2016b
16.73 (-12.85, 46.31)
39.18
Overall (I-squared = 59.0%, p = 0.087)
-9.07 (-42.32, 24.17)
100.00
NOTE: Weights are from random effects analysis
-100
-50
0
50
Lower in lung cancer
Higher in lung cancer

(ii)(d)

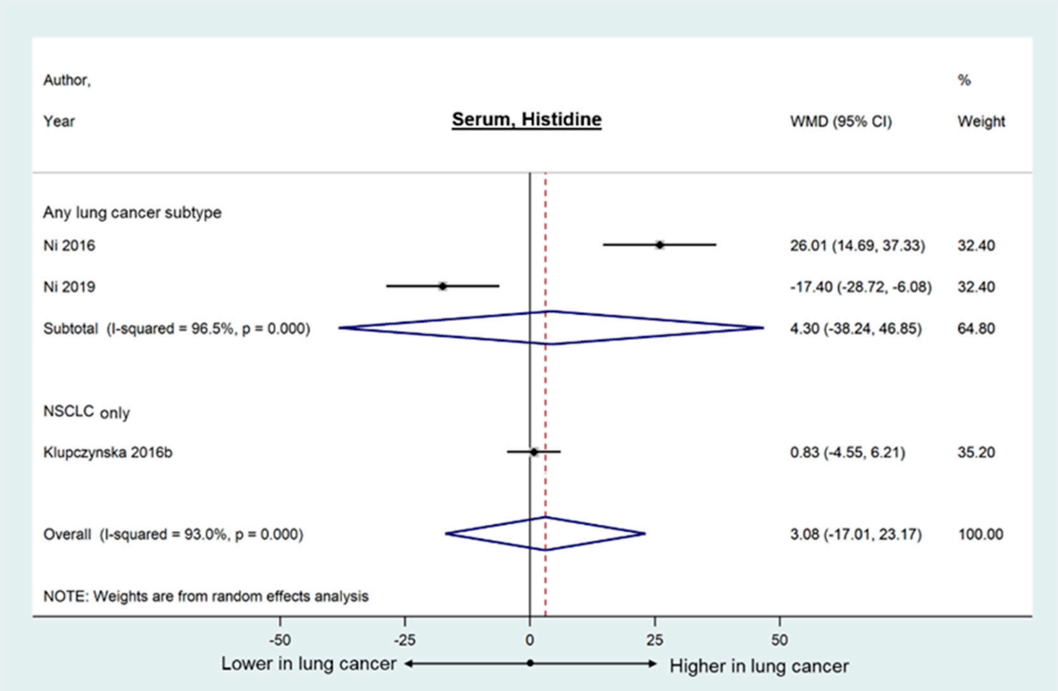
Author,
Year
Serum, Histidine
WMD (95% CI)
% Weight
Any lung cancer subtype
Ni 2016
26.01 (14.69, 37.33)
32.40
Ni 2019
-17.40 (-28.72, -6.08)
32.40
Subtotal (I-squared = 96.5%, p = 0.000)
4.30 (-38.24, 46.85)
64.80
NSCLC only
Klupczynska 2016b
0.83 (-4.55, 6.21)
35.20
Overall (I-squared = 93.0%, p = 0.000)
3.08 (-17.01, 23.17)
100.00
NOTE: Weights are from random effects analysis
-50
-25
0
25
50
Lower in lung cancer
Higher in lung cancer

(ii)(e)

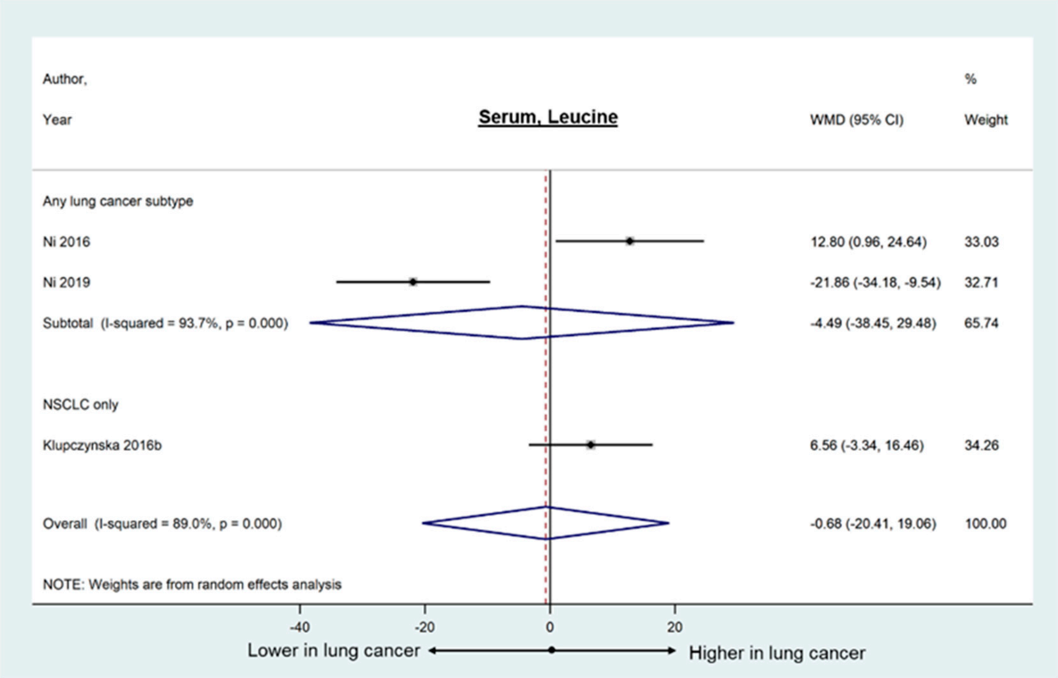
Author,
Year
Serum, Leucine
WMD (95% CI)
% Weight
Any lung cancer subtype
Ni 2016
12.80 (0.96, 24.64)
33.03
Ni 2019
-21.86 (-34.18, -9.54)
32.71
Subtotal (I-squared = 93.7%, p = 0.000)
-4.49 (-38.45, 29.48)
65.74
NSCLC only
Klupczynska 2016b
6.56 (-3.34, 16.46)
34.26
Overall (I-squared = 89.0%, p = 0.000)
-0.68 (-20.41, 19.06)
100.00
NOTE: Weights are from random effects analysis
-40
-20
0
20
Lower in lung cancer
Higher in lung cancer

(ii)(f)

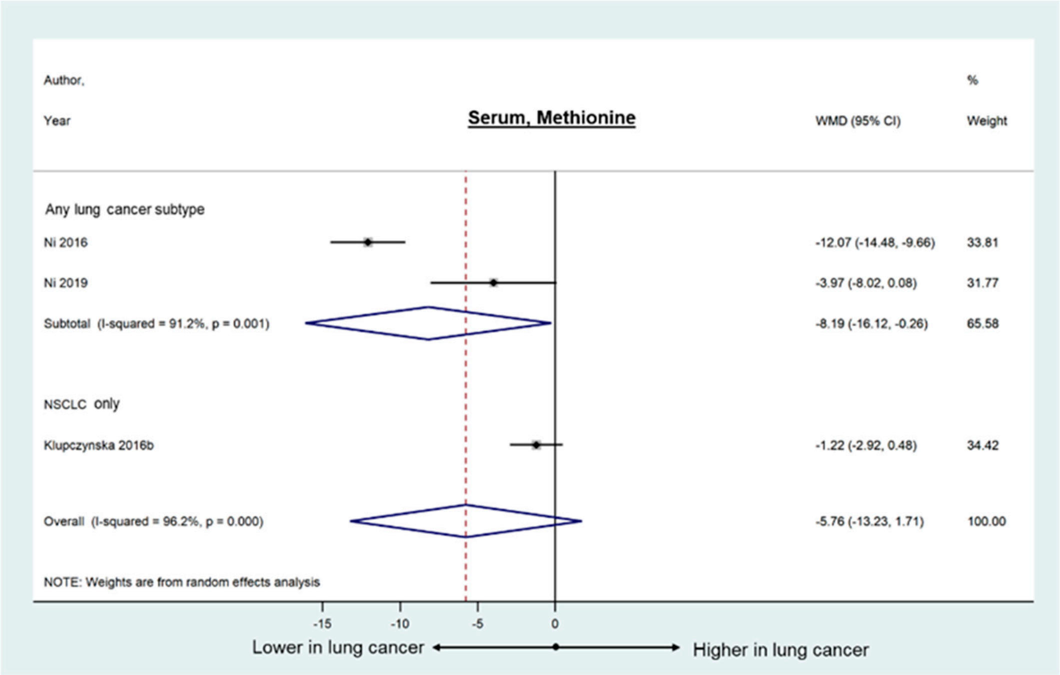
Author,
Year
Serum, Methionine
%
WMD (95% CI)
Weight
Any lung cancer subtype
Ni 2016
-12.07 (-14.48, -9.66)
33.81
Ni 2019
-3.97 (-8.02, 0.08)
31.77
Subtotal (I-squared = 91.2%, p = 0.001)
-8.19 (-16.12, -0.26)
65.58
NSCLC only
Klupczynska 2016b
-1.22 (-2.92, 0.48)
34.42
Overall (I-squared = 96.2%, p = 0.000)
-5.76 (-13.23, 1.71)
100.00
NOTE: Weights are from random effects analysis
-15
-10
-5
0
Lower in lung cancer
Higher in lung cancer

(ii)(g)

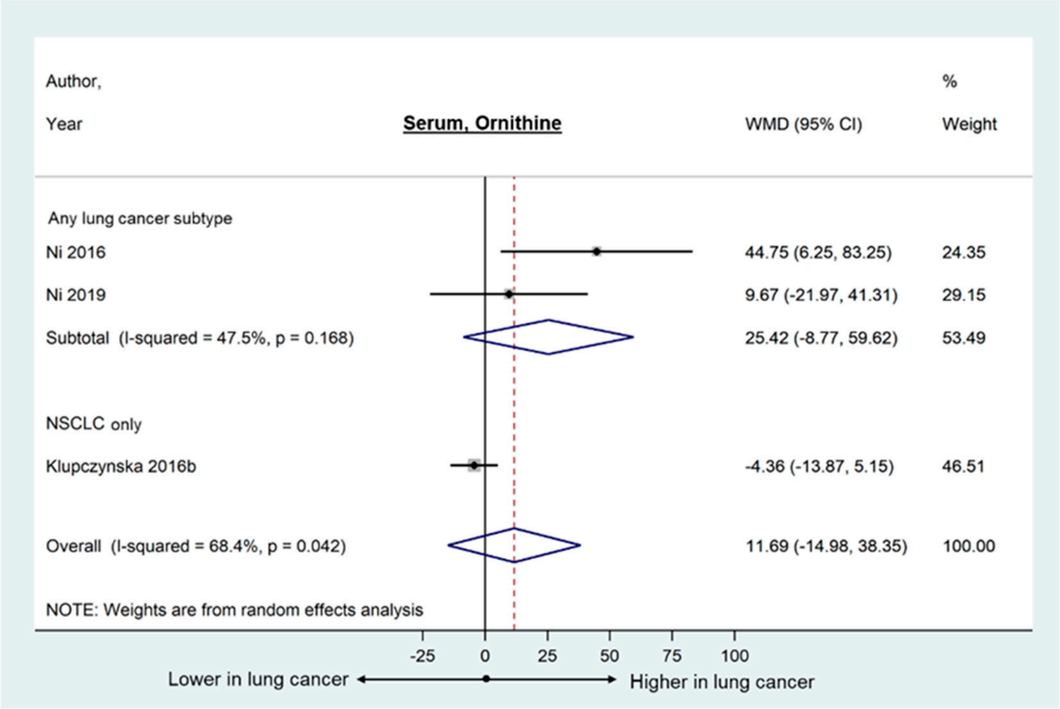
Author,
Year
Serum, Ornithine
%
WMD (95% CI)
Weight
Any lung cancer subtype
Ni 2016
44.75 (6.25, 83.25)
24.35
Ni 2019
9.67 (-21.97, 41.31)
29.15
Subtotal (I-squared = 47.5%, p = 0.168)
25.42 (-8.77, 59.62)
53.49
NSCLC only
Klupczynska 2016b
-4.36 (-13.87, 5.15)
46.51
Overall (I-squared = 68.4%, p = 0.042)
11.69 (-14.98, 38.35)
100.00
NOTE: Weights are from random effects analysis
-25
0
25
50
75
100
Lower in lung cancer
Higher in lung cancer

(ii)(h)

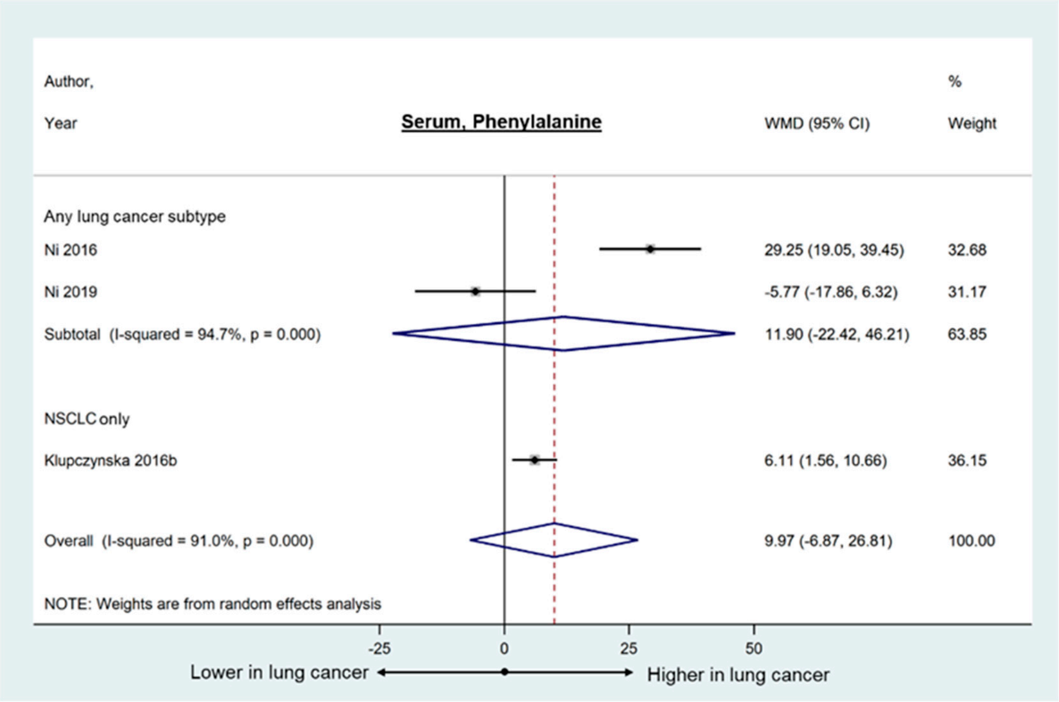
Author,
Year
Serum, Phenylalanine
WMD (95% CI)
%
Weight
Any lung cancer subtype
Ni 2016
29.25 (19.05, 39.45)
32.68
Ni 2019
-5.77 (-17.86, 6.32)
31.17
Subtotal (I-squared = 94.7%, p = 0.000)
11.90 (-22.42, 46.21)
63.85
NSCLC only
Klupczynska 2016b
6.11 (1.56, 10.66)
36.15
Overall (I-squared = 91.0%, p = 0.000)
9.97 (-6.87, 26.81)
100.00
NOTE: Weights are from random effects analysis
-25
0
25
50
Lower in lung cancer
Higher in lung cancer

(ii)(i)

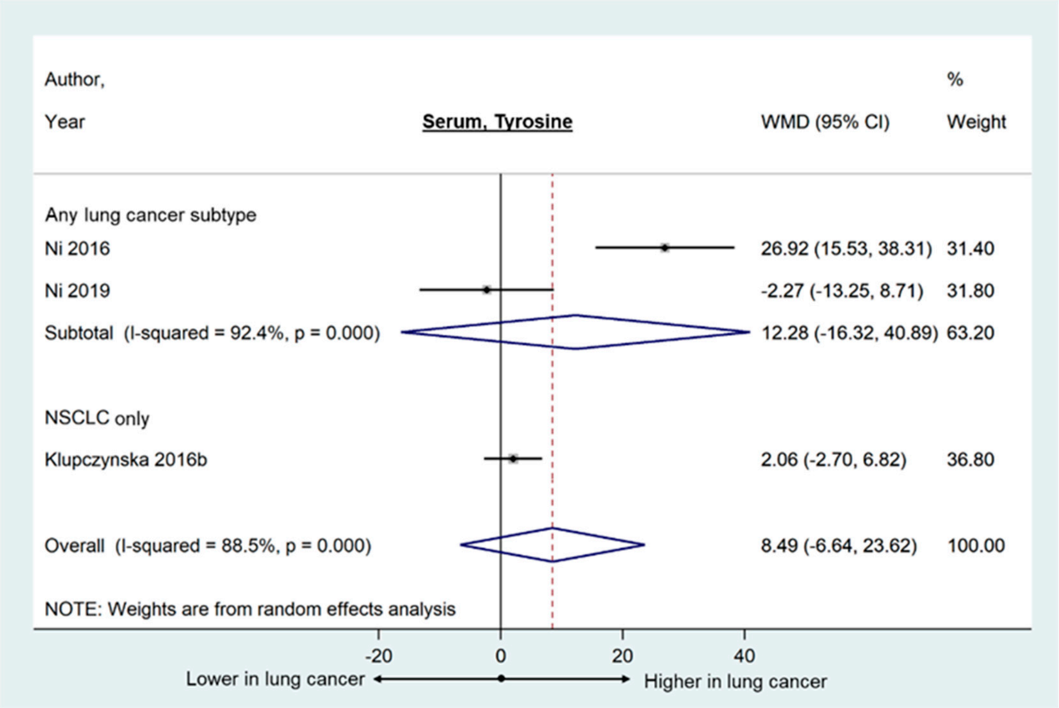
Author,
Year
Serum, Tyrosine
WMD (95% CI)
%
Weight
Any lung cancer subtype
Ni 2016
26.92 (15.53, 38.31)
31.40
Ni 2019
-2.27 (-13.25, 8.71)
31.80
Subtotal (I-squared = 92.4%, p = 0.000)
12.28 (-16.32, 40.89)
63.20
NSCLC only
Klupczynska 2016b
2.06 (-2.70, 6.82)
36.80
Overall (I-squared = 88.5%, p = 0.000)
8.49 (-6.64, 23.62)
100.00
NOTE: Weights are from random effects analysis
-20
0
20
40
Lower in lung cancer
Higher in lung cancer

(ii)(j)

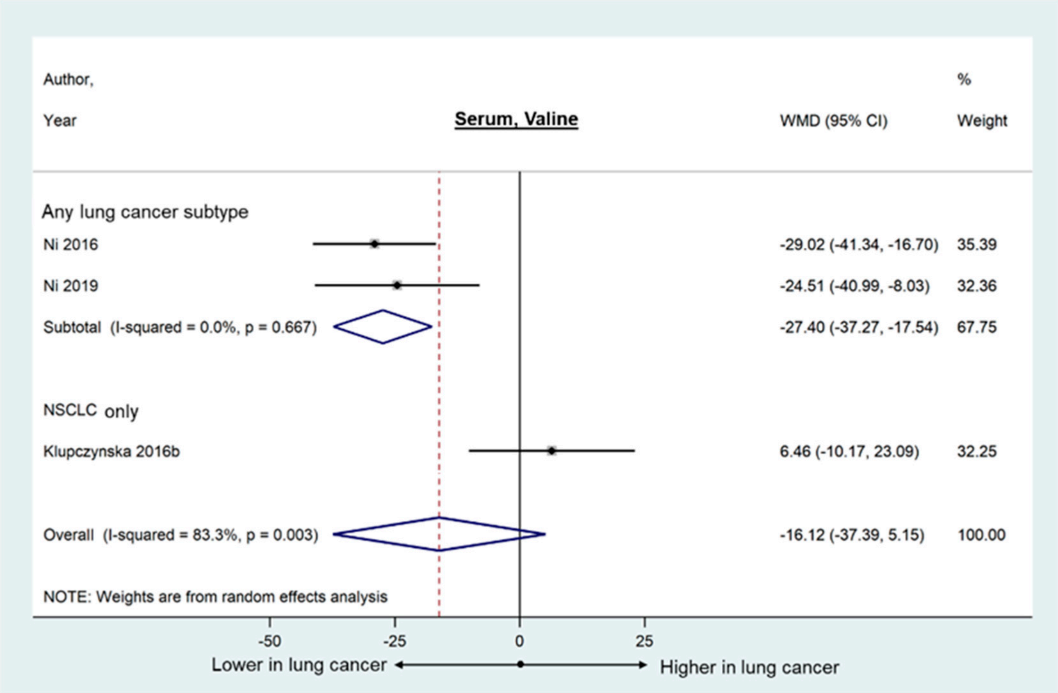
Author,
Year
Serum, Valine
WMD (95% CI)
%
Weight
Any lung cancer subtype
Ni 2016
-29.02 (-41.34, -16.70)
35.39
Ni 2019
-24.51 (-40.99, -8.03)
32.36
Subtotal (I-squared = 0.0%, p = 0.667)
-27.40 (-37.27, -17.54)
67.75
NSCLC only
Klupczynska 2016b
6.46 (-10.17, 23.09)
32.25
Overall (I-squared = 83.3%, p = 0.003)
-16.12 (-37.39, 5.15)
100.00
NOTE: Weights are from random effects analysis
-50
-25
0
25
Lower in lung cancer
Higher in lung cancer

(ii)(**k**)

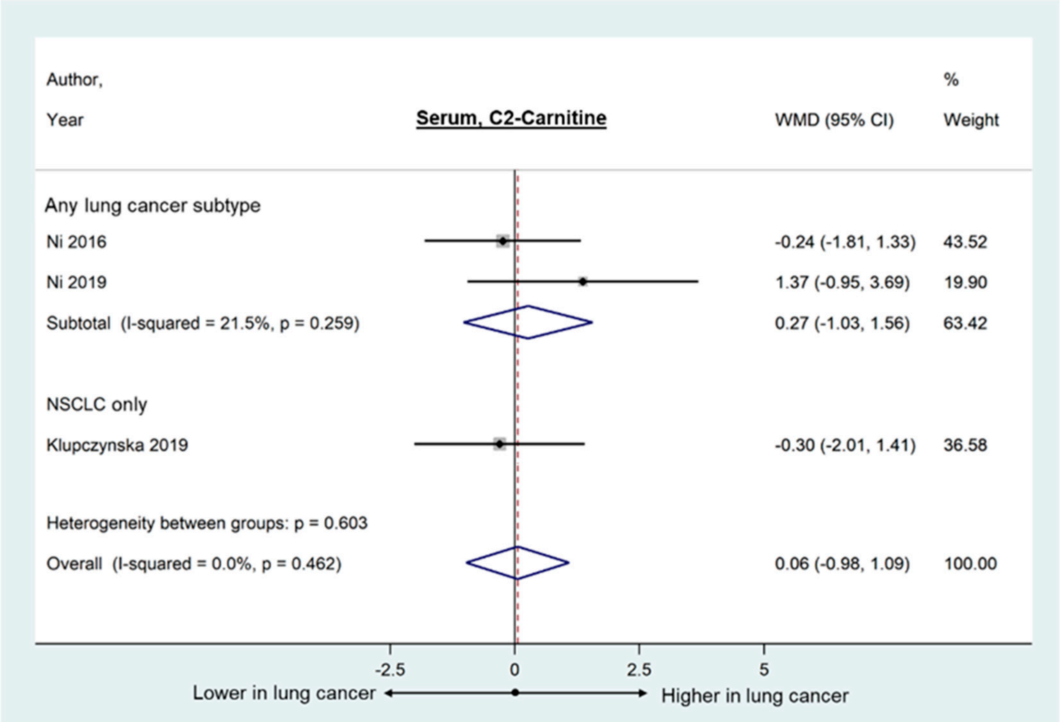
Author,
Year
Serum, C2-Carnitine
WMD (95% CI)
%
Weight
Any lung cancer subtype
Ni 2016
-0.24 (-1.81, 1.33)
43.52
Ni 2019
1.37 (-0.95, 3.69)
19.90
Subtotal (I-squared = 21.5%, p = 0.259)
0.27 (-1.03, 1.56)
63.42
NSCLC only
Klupczynska 2019
-0.30 (-2.01, 1.41)
36.58
Heterogeneity between groups: p = 0.603
Overall (I-squared = 0.0%, p = 0.462)
0.06 (-0.98, 1.09)
100.00
-2.5
0
2.5
5
Lower in lung cancer
Higher in lung cancer

(iii)(**a**)

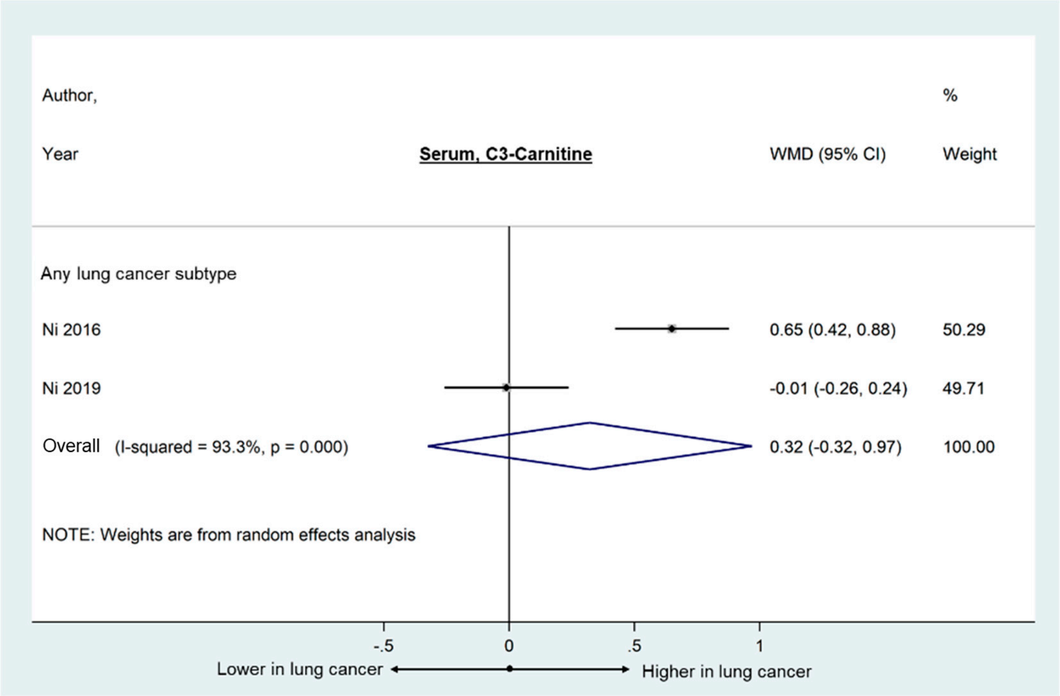
Author,
Year
Serum, C3-Carnitine
WMD (95% CI)
%
Weight
Any lung cancer subtype
Ni 2016
0.65 (0.42, 0.88)
50.29
Ni 2019
-0.01 (-0.26, 0.24)
49.71
Overall (I-squared = 93.3%, p = 0.000)
0.32 (-0.32, 0.97)
100.00
NOTE: Weights are from random effects analysis
-.5
0
.5
1
Lower in lung cancer
Higher in lung cancer

(iii)(**b**)

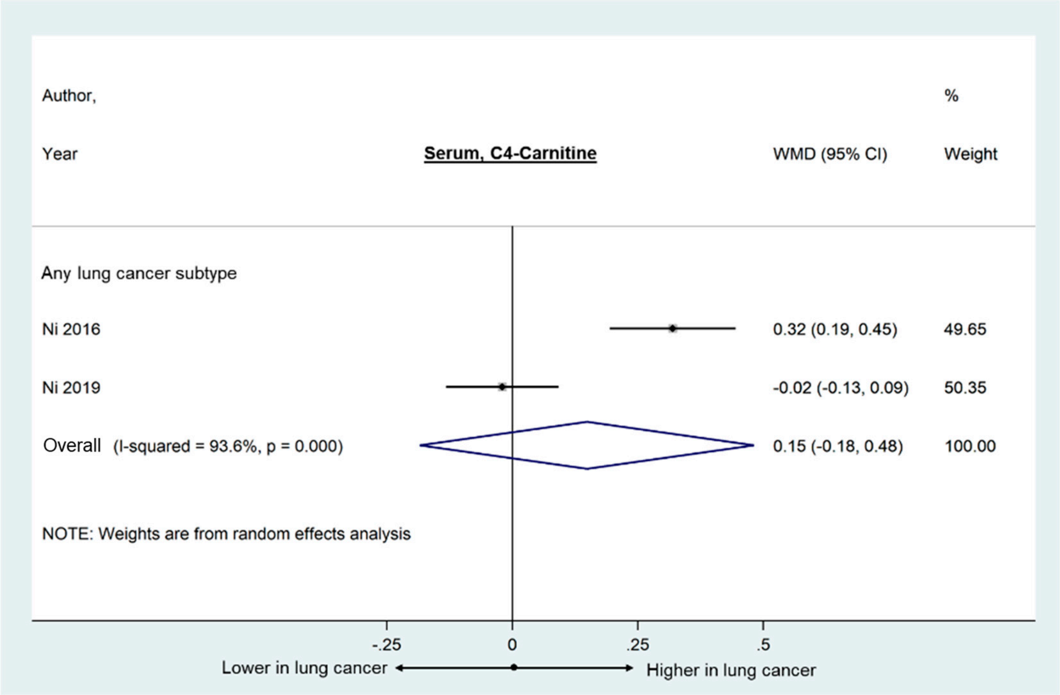

Author,
Year
Serum, C4-Carnitine
WMD (95% CI)
%
Weight
Any lung cancer subtype
Ni 2016
0.32 (0.19, 0.45)
49.65
Ni 2019
-0.02 (-0.13, 0.09)
50.35
Overall (I-squared = 93.6%, p = 0.000)
0.15 (-0.18, 0.48)
100.00
NOTE: Weights are from random effects analysis
-.25
0
.25
.5
Lower in lung cancer
Higher in lung cancer

(iii)(c)

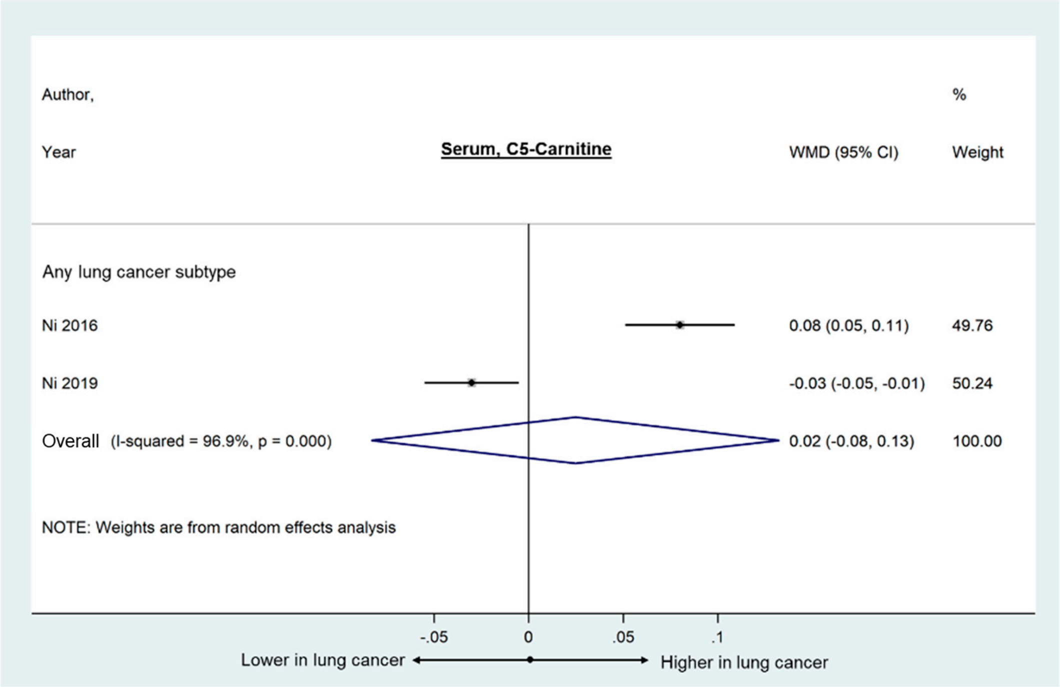

Author,
Year
Serum, C5-Carnitine
WMD (95% CI)
%
Weight
Any lung cancer subtype
Ni 2016
0.08 (0.05, 0.11)
49.76
Ni 2019
-0.03 (-0.05, -0.01)
50.24
Overall (I-squared = 96.9%, p = 0.000)
0.02 (-0.08, 0.13)
100.00
NOTE: Weights are from random effects analysis
-.05
0
.05
.1
Lower in lung cancer
Higher in lung cancer

(iii)(d)

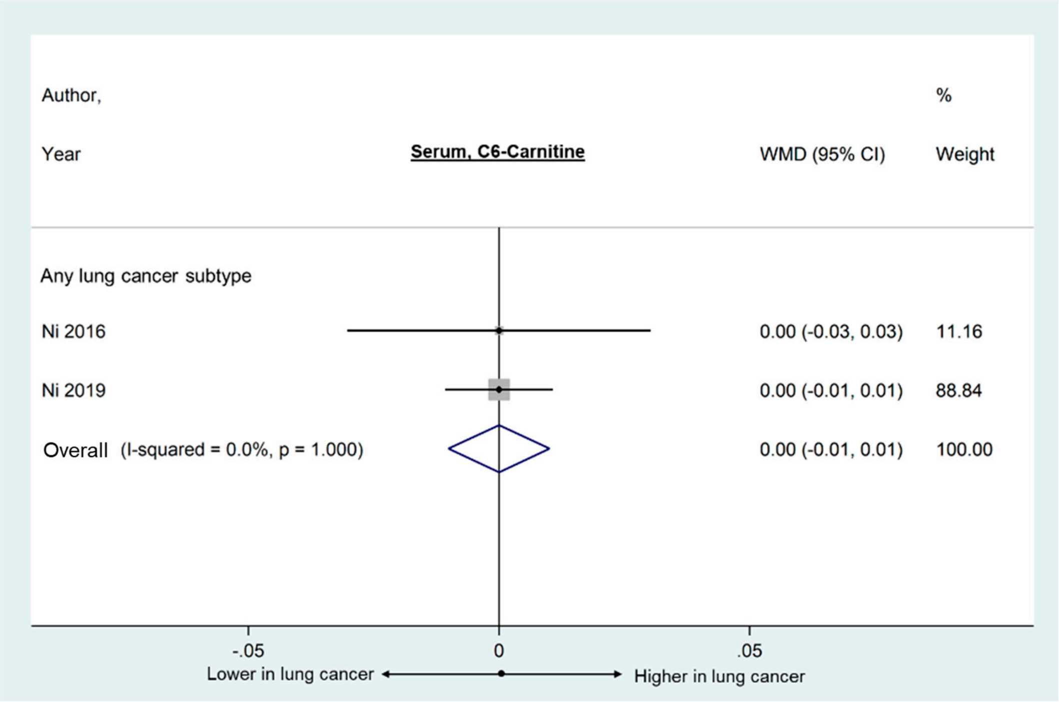
Author,
Year
%
Weight
Serum, C6-Carnitine
WMD (95% CI)
Any lung cancer subtype
Ni 2016
0.00 (-0.03, 0.03)
11.16
Ni 2019
0.00 (-0.01, 0.01)
88.84
Overall (I-squared = 0.0%, p = 1.000)
0.00 (-0.01, 0.01)
100.00
-.05
0
.05
Lower in lung cancer
Higher in lung cancer

(iii)(e)

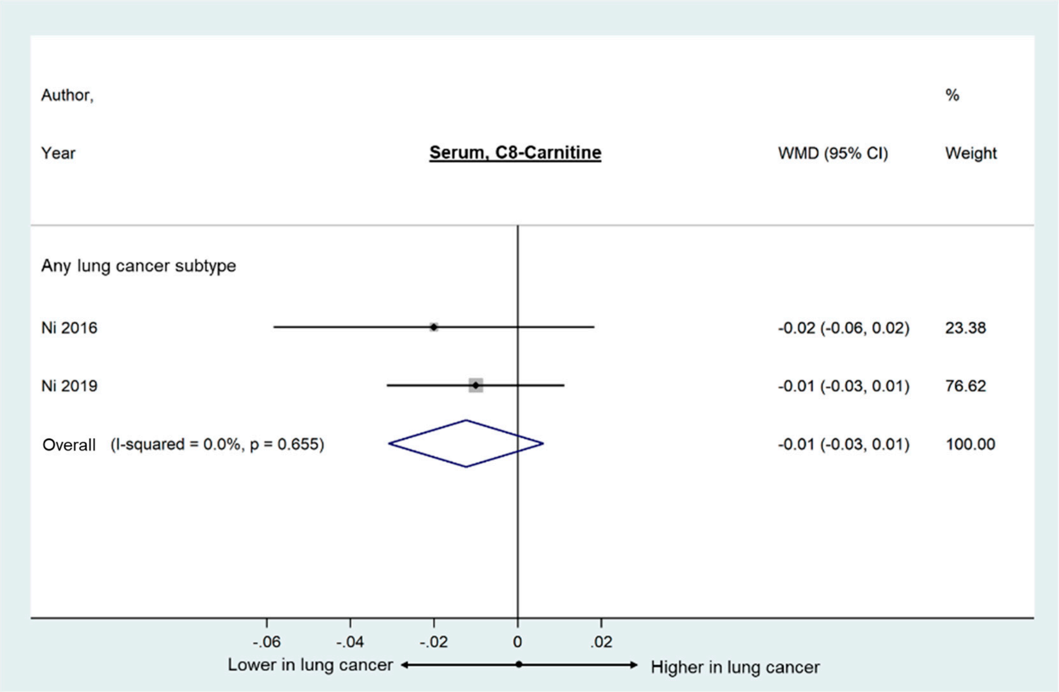
Author,
Year
%
Weight
Serum, C8-Carnitine
WMD (95% CI)
Any lung cancer subtype
Ni 2016
-0.02 (-0.06, 0.02)
23.38
Ni 2019
-0.01 (-0.03, 0.01)
76.62
Overall (I-squared = 0.0%, p = 0.655)
-0.01 (-0.03, 0.01)
100.00
-.06
-.04
-.02
0
.02
Lower in lung cancer
Higher in lung cancer

(iii)(f)

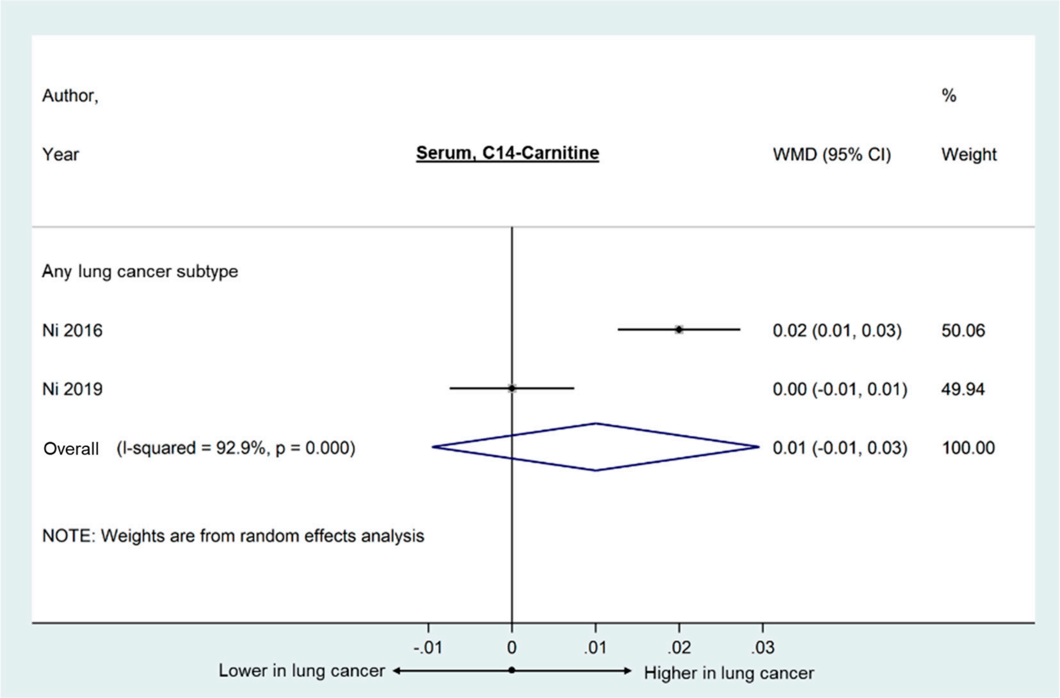

(iii)(g)

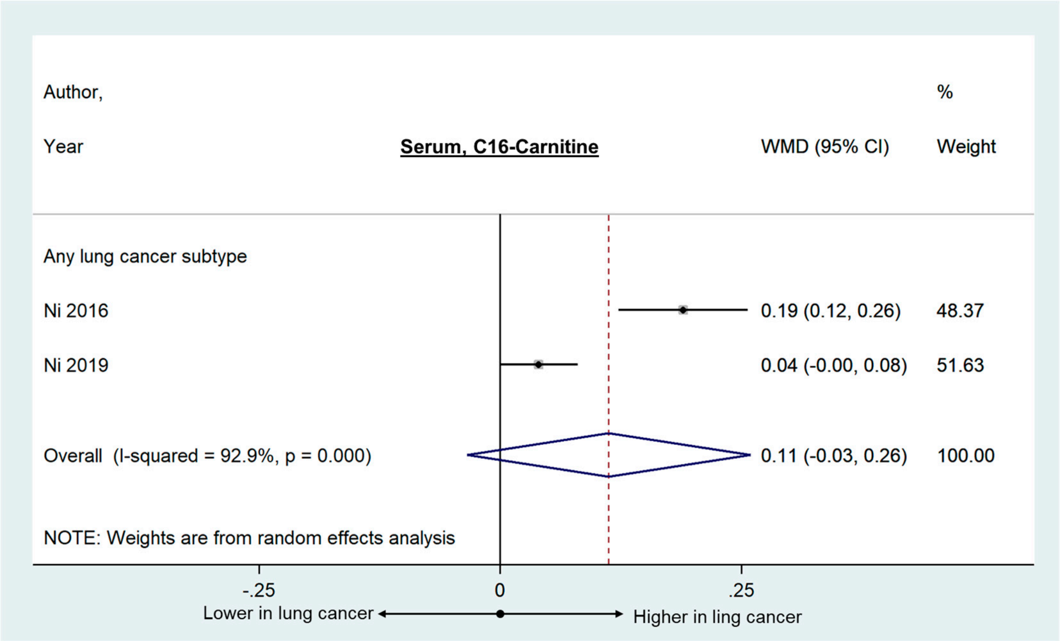

(iii)(h)

Figure S5. Weighted mean difference (WMD) and 95% confidence interval (CI) of the metabolite concentration between lung cancer patients and healthy controls for metabolites that did not achieve statistical significance.

Data are presented as μmol/L.

The metabolites are categorized as (i) plasma amino acids, (ii) serum amino acids and (iii) serum carnitines.

The (i) plasma amino acids are (a) alanine, (b) arginine, (c) asparagine, (d) aspartic acid, (e) citrulline, (f) glutamine, (g) glutamic acid, (h) glycine, (i) histidine, (j) isoleucine, (k) leucine, (l) lysine, (m) ornithine, (n) phenylalanine, (o) serine, (p) threonine, (q) tyrosine and (r) valine.

The (ii) serum amino acids are (a) alanine, (b) arginine, (c) citrulline, (d) glycine, (e) histidine, (f) leucine, (g) methionine, (h) ornithine, (i) phenylalanine, (j) tyrosine and (k) valine.

The (iii) serum carnitines are (a) C2, (b) C3, (c) C4, (d) C5, (e) C6, (f) C8, (g) C14 and (h) C16.

References

1. Liberati, A.; Altman, D.G.; Tetzlaff, J.; Mulrow, C.; Gøtzsche, P.C.; Ioannidis, J.P.A.; Clarke, M.; Devereaux, P.J.; Kleijnen, J.; Moher, D. The PRISMA statement for reporting systematic reviews and meta-analyses of studies that evaluate healthcare interventions: explanation and elaboration. *BMJ : British medical journal* **2009**, *339*.
2. Higgins, J.; Thomas, J.; Chandler, J.; Cumpston, M.; Li, T.; Page, M.; Welch, V. *Cochrane Handbook for Systematic Reviews of Interventions. 2nd Edition.*; John Wiley & Sons: Chichester (UK), 2019.
3. Chuang, S.C.; Fanidi, A.; Ueland, P.M.; Relton, C.; Midttun, O.; Vollset, S.E.; Gunter, M.J.; Seckl, M.J.; Travis, R.C.; Wareham, N., et al. Circulating biomarkers of tryptophan and the kynurenine pathway and lung cancer risk. *Cancer Epidemiol Biomarkers Prev* **2014**, *23*, 461-468, doi:10.1158/1055-9965.Epi-13-0770.
4. Afzal, S.; Bojesen, S.E.; Nordestgaard, B.G. Low Plasma 25-Hydroxyvitamin D and Risk of Tobacco-Related Cancer. *Clinical Chemistry* **2013**, *59*, 771-780, doi:10.1373/clinchem.2012.201939.
5. Wang, X.; Cui, J.; Gu, J.; He, H.; Li, B.; Li, W. Plasma 25-hydroxyvitamin D deficiency is associated with the risk of non-small cell lung cancer in a Chinese population. *Cancer Biomarkers* **2015**, *15*, 663-668, doi:10.3233/CBM-150506.
6. Haznadar, M.; Krausz, K.W.; Margono, E.; Diehl, C.M.; Bowman, E.D.; Manna, S.K.; Robles, A.I.; Ryan, B.M.; Gonzalez, F.J.; Harris, C.C. Inverse association of vitamin D3 levels with lung cancer mediated by genetic variation. *Cancer Med* **2018**, *7*, 2764-2775, doi:10.1002/cam4.1444.
7. Kilkkinen, A.; Knekt, P.; Heliovaara, M.; Rissanen, H.; Marniemi, J.; Hakulinen, T.; Aromaa, A. Vitamin D status and the risk of lung cancer: a cohort study in Finland. *Cancer Epidemiol Biomarkers Prev* **2008**, *17*, 3274-3278, doi:10.1158/1055-9965.Epi-08-0199.
8. Weinstein, S.J.; Yu, K.; Horst, R.L.; Parisi, D.; Virtamo, J.; Albanes, D. Serum 25-hydroxyvitamin D and risk of lung cancer in male smokers: a nested case-control study. *PLoS One* **2011**, *6*, e20796, doi:10.1371/journal.pone.0020796.
9. Skaaby, T.; Husemoen, L.L.; Thuesen, B.H.; Pisinger, C.; Jorgensen, T.; Roswall, N.; Larsen, S.C.; Linneberg, A. Prospective population-based study of the association between serum 25-hydroxyvitamin-D levels and the incidence of specific types of cancer. *Cancer Epidemiol Biomarkers Prev* **2014**, *23*, 1220-1229, doi:10.1158/1055-9965.Epi-14-0007.
10. Ordóñez-Mena, J.M.; Schöttker, B.; Fedirko, V.; Jenab, M.; Olsen, A.; Halkjær, J.; Kampman, E.; de Groot, L.; Jansen, E.; Bueno-de-Mesquita, H.B., et al. Pre-diagnostic vitamin D concentrations and cancer risks in older individuals: an analysis of cohorts participating in the CHANCES consortium. *European Journal of Epidemiology* **2016**, *31*, 311-323, doi:10.1007/s10654-015-0040-7.
11. Johansson, M.; Relton, C.; Ueland, P.M.; Vollset, S.E.; Midttun, O.; Nygard, O.; Slimani, N.; Boffetta, P.; Jenab, M.; Clavel-Chapelon, F., et al. Serum B vitamin levels and risk of lung cancer. *Jama* **2010**, *303*, 2377-2385, doi:10.1001/jama.2010.808.
12. Boffetta, P.; Clark, S.; Shen, M.; Gislefoss, R.; Peto, R.; Andersen, A. Serum cotinine level as predictor of lung cancer risk. *Cancer Epidemiol Biomarkers Prev* **2006**, *15*, 1184-1188, doi:10.1158/1055-9965.Epi-06-0032.
13. Church, T.R.; Anderson, K.E.; Caporaso, N.E.; Geisser, M.S.; Le, C.T.; Zhang, Y.; Benoit, A.R.; Carmella, S.G.; Hecht, S.S. A prospectively measured serum biomarker for a tobacco-specific carcinogen and lung cancer in smokers. *Cancer Epidemiol Biomarkers Prev* **2009**, *18*, 260-266, doi:10.1158/1055-9965.Epi-08-0718.
14. Gao, X.; Wilsgaard, T.; Jansen, E.H.; Holleczek, B.; Zhang, Y.; Xuan, Y.; Anusruti, A.; Brenner, H.; Schottker, B. Pre-diagnostic derivatives of reactive oxygen metabolites and the occurrence of lung, colorectal, breast and prostate cancer: An individual participant data meta-analysis of two large population-based studies. *Int J Cancer* **2019**, *145*, 49-57, doi:10.1002/ijc.32073.
15. Larose, T.L.; Guida, F.; Fanidi, A.; Langhammer, A.; Kveem, K.; Stevens, V.L.; Jacobs, E.J.; Smith-Warner, S.A.; Giovannucci, E.; Albanes, D., et al. Circulating cotinine concentrations and lung cancer risk in the Lung Cancer Cohort Consortium (LC3). *Int J Epidemiol* **2018**, *47*, 1760-1771, doi:10.1093/ije/dyy100.
16. Timofeeva, M.N.; McKay, J.D.; Smith, G.D.; Johansson, M.; Byrnes, G.B.; Chabrier, A.; Relton, C.; Ueland, P.M.; Vollset, S.E.; Midttun, Ø., et al. Genetic polymorphisms in 15q25 and 19q13 loci, cotinine levels, and risk of lung cancer in EPIC. *Cancer epidemiology, biomarkers & prevention : a publication of the American Association for Cancer Research, cosponsored by the American Society of Preventive Oncology* **2011**, *20*, 2250-2261, doi:10.1158/1055-9965.EPI-11-0496.
17. Fanidi, A.; Muller, D.C.; Yuan, J.-M.; Stevens, V.L.; Weinstein, S.J.; Albanes, D.; Prentice, R.; Thomsen, C.A.; Pettinger, M.; Cai, Q., et al. Circulating Folate, Vitamin B6, and Methionine in Relation to Lung Cancer Risk

in the Lung Cancer Cohort Consortium (LC3). *Journal of the National Cancer Institute* **2018**, *110*, 57-67, doi:10.1093/jnci/djx119.

18. Eom, S.Y.; Yim, D.H.; Moon, S.I.; Youn, J.W.; Kwon, H.J.; Oh, H.C.; Yang, J.J.; Park, S.K.; Yoo, K.Y.; Kim, H.S., et al. Polycyclic aromatic hydrocarbon-induced oxidative stress, antioxidant capacity, and the risk of lung cancer: a pilot nested case-control study. *Anticancer Res* **2013**, *33*, 3089-3097.
19. Yuan, J.M.; Butler, L.M.; Gao, Y.T.; Murphy, S.E.; Carmella, S.G.; Wang, R.; Nelson, H.H.; Hecht, S.S. Urinary metabolites of a polycyclic aromatic hydrocarbon and volatile organic compounds in relation to lung cancer development in lifelong never smokers in the Shanghai Cohort Study. *Carcinogenesis* **2014**, *35*, 339-345, doi:10.1093/carcin/bgt352.
20. Seow, W.J.; Shu, X.O.; Nicholson, J.K.; Holmes, E.; Walker, D.I.; Hu, W.; Cai, Q.; Gao, Y.T.; Xiang, Y.B.; Moore, S.C., et al. Association of Untargeted Urinary Metabolomics and Lung Cancer Risk Among Never-Smoking Women in China. *JAMA Netw Open* **2019**, *2*, e1911970, doi:10.1001/jamanetworkopen.2019.11970.
21. Loft, S.; Svoboda, P.; Kasai, H.; Tjonneland, A.; Moller, P.; Sorensen, M.; Overvad, K.; Autrup, H.; Raaschou-Nielsen, O. Prospective study of urinary excretion of 7-methylguanine and the risk of lung cancer: Effect modification by mu class glutathione-S-transferases. *Int J Cancer* **2007**, *121*, 1579-1584, doi:10.1002/ijc.22863.
22. Mathe, E.A.; Patterson, A.D.; Haznadar, M.; Manna, S.K.; Krausz, K.W.; Bowman, E.D.; Shields, P.G.; Idle, J.R.; Smith, P.B.; Anami, K., et al. Noninvasive urinary metabolomic profiling identifies diagnostic and prognostic markers in lung cancer. *Cancer Res* **2014**, *74*, 3259-3270, doi:10.1158/0008-5472.Can-14-0109.
23. Haznadar, M.; Cai, Q.; Krausz, K.W.; Bowman, E.D.; Margono, E.; Noro, R.; Thompson, M.D.; Mathe, E.A.; Munro, H.M.; Steinwandel, M.D., et al. Urinary Metabolite Risk Biomarkers of Lung Cancer: A Prospective Cohort Study. *Cancer Epidemiol Biomarkers Prev* **2016**, *25*, 978-986, doi:10.1158/1055-9965.Epi-15-1191.
24. de Waard, F.; Kemmeren, J.M.; van Ginkel, L.A.; Stolker, A.A. Urinary cotinine and lung cancer risk in a female cohort. *British journal of cancer* **1995**, *72*, 784-787, doi:10.1038/bjc.1995.411.
25. Yuan, J.M.; Nelson, H.H.; Carmella, S.G.; Wang, R.; Kuriger-Laber, J.; Jin, A.; Adams-Haduch, J.; Hecht, S.S.; Koh, W.P.; Murphy, S.E. CYP2A6 genetic polymorphisms and biomarkers of tobacco smoke constituents in relation to risk of lung cancer in the Singapore Chinese Health Study. *Carcinogenesis* **2017**, *38*, 411-418, doi:10.1093/carcin/bgx012.
26. Yuan, J.M.; Gao, Y.T.; Wang, R.; Chen, M.; Carmella, S.G.; Hecht, S.S. Urinary levels of volatile organic carcinogen and toxicant biomarkers in relation to lung cancer development in smokers. *Carcinogenesis* **2012**, *33*, 804-809, doi:10.1093/carcin/bgs026.
27. London, S.J.; Yuan, J.-M.; Chung, F.-L.; Gao, Y.-T.; Coetzee, G.A.; Ross, R.K.; Yu, M.C. Isothiocyanates, glutathione S-transferase M1 and T1 polymorphisms, and lung-cancer risk: a prospective study of men in Shanghai, China. *The Lancet* **2000**, *356*, 724-729, doi:10.1016/S0140-6736(00)02631-3.
28. Gao, X.; Xuan, Y.; Benner, A.; Anusruti, A.; Brenner, H.; Schottker, B. Nitric Oxide Metabolites and Lung Cancer Incidence: A Matched Case-Control Study Nested in the ESTHER Cohort. *Oxid Med Cell Longev* **2019**, *2019*, 6470950, doi:10.1155/2019/6470950.
29. Yuan, J.M.; Gao, Y.T.; Murphy, S.E.; Carmella, S.G.; Wang, R.; Zhong, Y.; Moy, K.A.; Davis, A.B.; Tao, L.; Chen, M., et al. Urinary levels of cigarette smoke constituent metabolites are prospectively associated with lung cancer development in smokers. *Cancer Res* **2011**, *71*, 6749-6757, doi:10.1158/0008-5472.Can-11-0209.
30. Yuan, J.M.; Nelson, H.H.; Butler, L.M.; Carmella, S.G.; Wang, R.; Kuriger-Laber, J.K.; Adams-Haduch, J.; Hecht, S.S.; Gao, Y.T.; Murphy, S.E. Genetic determinants of cytochrome P450 2A6 activity and biomarkers of tobacco smoke exposure in relation to risk of lung cancer development in the Shanghai cohort study. *Int J Cancer* **2016**, *138*, 2161-2171, doi:10.1002/ijc.29963.
31. Ellard, G.A.; de Waard, F.; Kemmeren, J.M. Urinary nicotine metabolite excretion and lung cancer risk in a female cohort. *Br J Cancer* **1995**, *72*, 788-791, doi:10.1038/bjc.1995.412.
32. Preti, G.; Labows, J.N.; Kostelc, J.G.; Aldinger, S.; Daniele, R. Analysis of lung air from patients with bronchogenic carcinoma and controls using gas chromatography-mass spectrometry. *J Chromatogr* **1988**, *432*, 1-11, doi:10.1016/s0378-4347(00)80627-1.
33. Masri, F.A.; Comhair, S.A.; Koeck, T.; Xu, W.; Janocha, A.; Ghosh, S.; Dweik, R.A.; Golish, J.; Kinter, M.; Stuehr, D.J., et al. Abnormalities in nitric oxide and its derivatives in lung cancer. *Am J Respir Crit Care Med* **2005**, *172*, 597-605, doi:10.1164/rccm.200411-1523OC.
34. Proenza, A.M.; Oliver, J.; Palou, A.; Roca, P. Breast and lung cancer are associated with a decrease in blood cell amino acid content. *The Journal of Nutritional Biochemistry* **2003**, *14*, 133-138, doi:10.1016/S0955-2863(02)00225-5.

35. Hendrick, A.M.; Mitchell, M.D.; Harris, A.L. Plasma prostaglandins in lung cancer. *Eur J Cancer Clin Oncol* **1988**, *24*, 1069-1071, doi:10.1016/0277-5379(88)90161-7.
36. Yue, X.; He, J.; Zhang, R.; Xu, J.; Zhou, Z.; Zhang, R.; Bi, N.; Wang, Z.; Sun, C.; Wang, L., et al. Biotransformation-based metabolomics profiling method for determining and quantitating cancer-related metabolites. *J Chromatogr A* **2018**, *1580*, 80-89, doi:10.1016/j.chroma.2018.10.034.
37. Miyagi, Y.; Higashiyama, M.; Gochi, A.; Akaike, M.; Ishikawa, T.; Miura, T.; Saruki, N.; Bando, E.; Kimura, H.; Imamura, F., et al. Plasma free amino acid profiling of five types of cancer patients and its application for early detection. *PLoS One* **2011**, *6*, e24143, doi:10.1371/journal.pone.0024143.
38. Shingyoji, M.; Iizasa, T.; Higashiyama, M.; Imamura, F.; Saruki, N.; Imaizumi, A.; Yamamoto, H.; Daimon, T.; Tochikubo, O.; Mitsushima, T., et al. The significance and robustness of a plasma free amino acid (PFAA) profile-based multiplex function for detecting lung cancer. *BMC cancer* **2013**, *13*, 77-77, doi:10.1186/1471-2407-13-77.
39. Kim, H.J.; Jang, S.H.; Ryu, J.-S.; Lee, J.E.; Kim, Y.C.; Lee, M.K.; Jang, T.W.; Lee, S.-Y.; Nakamura, H.; Nishikata, N., et al. The performance of a novel amino acid multivariate index for detecting lung cancer: A case control study in Korea. *Lung Cancer* **2015**, *90*, 522-527, doi:10.1016/j.lungcan.2015.10.006.
40. Pietzke, M.; Arroyo, S.F.; Sumpton, D.; Mackay, G.M.; Martin-Castillo, B.; Camps, J.; Joven, J.; Menendez, J.A.; Vazquez, A. Stratification of cancer and diabetes based on circulating levels of formate and glucose. *Cancer Metab* **2019**, *7*, 3, doi:10.1186/s40170-019-0195-x.
41. Zhang, Q.; Xu, H.; Liu, R.; Gao, P.; Yang, X.; Jin, W.; Zhang, Y.; Bi, K.; Li, Q. A Novel Strategy for Targeted Lipidomics Based on LC-Tandem-MS Parameters Prediction, Quantification, and Multiple Statistical Data Mining: Evaluation of Lysophosphatidylcholines as Potential Cancer Biomarkers. *Anal Chem* **2019**, *91*, 3389-3396, doi:10.1021/acs.analchem.8b04715.
42. Hu, S.; Shen, G.; Yin, S.; Xu, W.; Hu, B. Melatonin and tryptophan circadian profiles in patients with advanced non-small cell lung cancer. *Adv Ther* **2009**, *26*, 886-892, doi:10.1007/s12325-009-0068-8.
43. Klupczynska, A.; Derezinski, P.; Dyszkiewicz, W.; Pawlak, K.; Kasprzyk, M.; Kokot, Z.J. Evaluation of serum amino acid profiles' utility in non-small cell lung cancer detection in Polish population. *Lung Cancer* **2016**, *100*, 71-76, doi:10.1016/j.lungcan.2016.04.008.
44. Klupczynska, A.; Plewa, S.; Dyszkiewicz, W.; Kasprzyk, M.; Sytek, N.; Kokot, Z.J. Determination of low-molecular-weight organic acids in non-small cell lung cancer with a new liquid chromatography-tandem mass spectrometry method. *J Pharm Biomed Anal* **2016**, *129*, 299-309, doi:10.1016/j.jpba.2016.07.028.
45. Klupczynska, A.; Plewa, S.; Kasprzyk, M.; Dyszkiewicz, W.; Kokot, Z.J.; Matysiak, J. Serum lipidome screening in patients with stage I non-small cell lung cancer. *Clin Exp Med* **2019**, *19*, 505-513, doi:10.1007/s10238-019-00566-7.
46. Ni, J.; Xu, L.; Li, W.; Wu, L. Simultaneous determination of thirteen kinds of amino acid and eight kinds of acylcarnitine in human serum by LC-MS/MS and its application to measure the serum concentration of lung cancer patients. *Biomed Chromatogr* **2016**, *30*, 1796-1806, doi:10.1002/bmc.3755.
47. Ni, J.; Xu, L.; Li, W.; Zheng, C.; Wu, L. Targeted metabolomics for serum amino acids and acylcarnitines in patients with lung cancer. *Exp Ther Med* **2019**, *18*, 188-198, doi:10.3892/etm.2019.7533.
48. Esme, H.; Cemek, M.; Sezer, M.; Saglam, H.; Demir, A.; Melek, H.; Unlu, M. High levels of oxidative stress in patients with advanced lung cancer. *Respirology* **2008**, *13*, 112-116, doi:10.1111/j.1440-1843.2007.01212.x.
49. Gencer, M.; Ceylan, E.; Aksoy, N.; Uzun, K. Association of serum reactive oxygen metabolite levels with different histopathological types of lung cancer. *Respiration* **2006**, *73*, 520-524, doi:10.1159/000088895.
50. Kami, K.; Fujimori, T.; Sato, H.; Sato, M.; Yamamoto, H.; Ohashi, Y.; Sugiyama, N.; Ishihama, Y.; Onozuka, H.; Ochiai, A., et al. Metabolomic profiling of lung and prostate tumor tissues by capillary electrophoresis time-of-flight mass spectrometry. *Metabolomics* **2013**, *9*, 444-453, doi:10.1007/s11306-012-0452-2.
51. Kukreja, S.C.; Shemerdiak, W.P.; York, P.A.J.; Lad, T.E.; Abramson, E.C.; Thomas, P.A.; Mir, J. Presence of prostaglandin E in lung tumors from normocalcemic patients. *The American Journal of Medicine* **1982**, *72*, 737-742, doi:10.1016/0002-9343(82)90538-1.
52. Okur, H.K.; Yuksel, M.; Lacin, T.; Baysungur, V.; Okur, E. Detection of reactive oxygen metabolites in malignant and adjacent normal tissues of patients with lung cancer. *World J Surg Oncol* **2013**, *11*, 9, doi:10.1186/1477-7819-11-9.
53. Zhang, T.; Lai, B.; Duan, L.; Wang, X. [Detection of metabolites of tobacco-specific nitrosamine 4-(methylnitrosamino)-1-(3-pyridyl)-1-butanone in lung cancer smokers' urine]. *Zhongguo Fei Ai Za Zhi* **2006**, *9*, 231-235, doi:10.3779/j.issn.1009-3419.2006.03.04.

54. Hwang, S.H.; Ryu, H.J.; Kang, S.J.; Yun, E.H.; Lim, M.K.; Kim, H.T.; Lee, J.S.; Lee, D.H. Levels of tobacco-specific metabolites among non-smoking lung cancer cases at diagnosis: case-control findings. *Asian Pac J Cancer Prev* **2014**, *14*, 6591-6593, doi:10.7314/apjcp.2013.14.11.6591.
55. Kawamoto, H.; Hara, H.; Araya, J.; Ichikawa, A.; Fujita, Y.; Utsumi, H.; Hashimoto, M.; Wakui, H.; Minagawa, S.; Numata, T., et al. Prostaglandin E-Major Urinary Metabolite (PGE-MUM) as a Tumor Marker for Lung Adenocarcinoma. *Cancers (Basel)* **2019**, *11*, doi:10.3390/cancers11060768.

© 2020 by the authors. Licensee MDPI, Basel, Switzerland. This article is an open access article distributed under the terms and conditions of the Creative Commons Attribution (CC BY) license (http://creativecommons.org/licenses/by/4.0/).
